# Supplementary material for: Hippocampal ripples initiate cortical dimensionality expansion for memory retrieval
Source: Nat Commun. 2026 Jul 20;17:6677. doi: 10.1038/s41467-026-75345-6 (PMC13385388; doi:10.1038/s41467-026-75345-6)
Supplement: Supplementary file 1 — Supplementary Information [file 41467_2026_75345_MOESM1_ESM.pdf]

Supplementary Information

Hippocampal ripples initiate cortical dimensionality expansion for memory retrieval

Casper Kerrén[1*], Sebastian Michelmann[2] & Christian F Doeller[1,3]

Affiliations:

[1] *Max Planck Institute for Human Cognitive and Brain Sciences; Leipzig, Germany*

[2] *Department of Psychology, New York University; New York City, New York*

[3] *Kavli Institute for Systems Neuroscience, Centre for Neural Computation, Egil and Pauline Braathen and Fred Kavli Centre for Cortical Microcircuits, Jebsen Centre for Alzheimer's Disease, NTNU Norwegian University of Science and Technology; Trondheim, Norway*

*Corresponding author. Email: kerren@cbs.mpg.de

Summary

How are past experiences reconstructed from memory? Learning is thought to compress external inputs into low-dimensional hippocampal representations, later expanded into high-dimensional cortical activity during recall. Hippocampal ripples, brief high-frequency bursts linked to retrieval, may initiate this expansion. Analysing intracranial EEG data from patients with pharmaco-resistant epilepsy during an episodic memory task, we found that cortical dimensionality increased following ripple events during correct, but not incorrect, retrieval. This expansion correlated with faster reaction times and reinstatement of the target association. Crucially, hippocampal theta and cortical gamma phase-amplitude coupling emerged after ripples but before cortical expansion, suggesting a mechanism for ripple-driven communication. Ripple events also marked the separation of task-relevant variables in cortical state space, revealing how hippocampal output reshapes the geometry of memory representations to support successful recall.

Keywords

Episodic memory, intracranial EEG, neural transformation, hippocampal ripples, theta-gamma coupling.

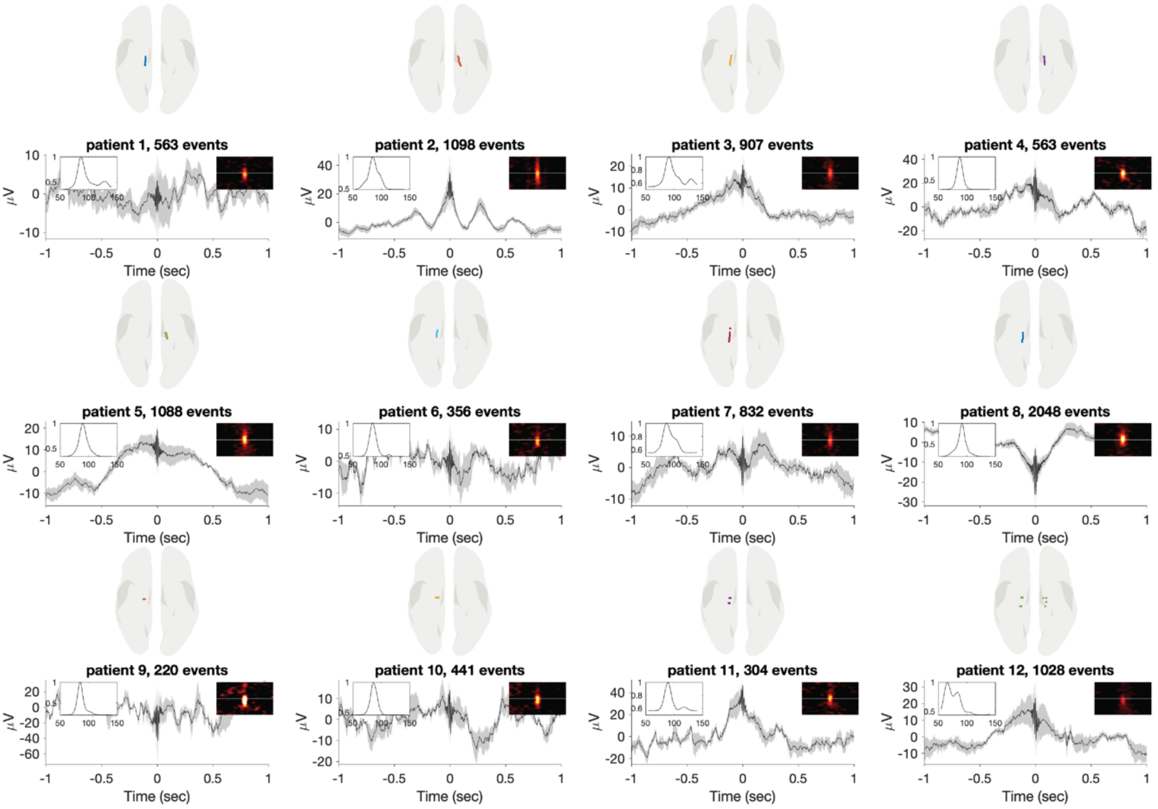

Supplementary Figure 1 | Electrode implantation, ripple counts, and spectral characteristics per participant. Each panel displays data from a single participant. The top row illustrates electrode implantation sites within the hippocampus. The middle row shows the number of detected ripple events. The bottom row presents the average ripple waveform alongside the corresponding time-frequency and spectral power profile.

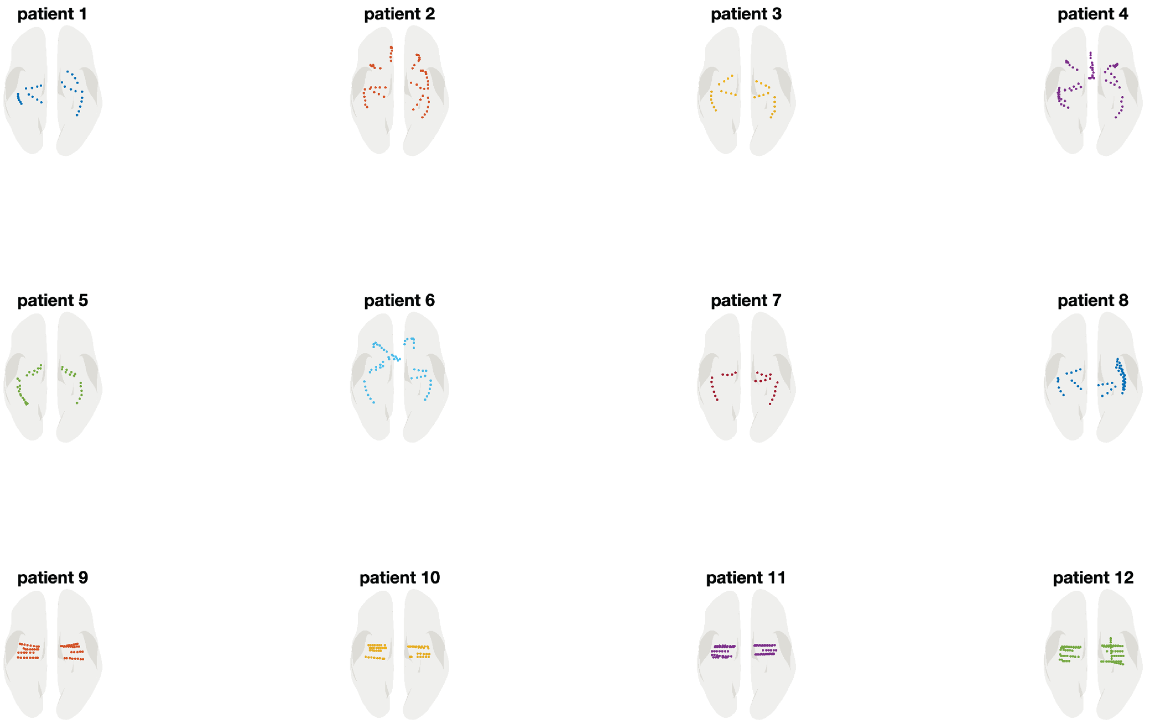

Supplementary Figure 2 | Electrode implantation extra-hippocampal regions. Each panel displays extra-hippocampal electrodes from a single participant.

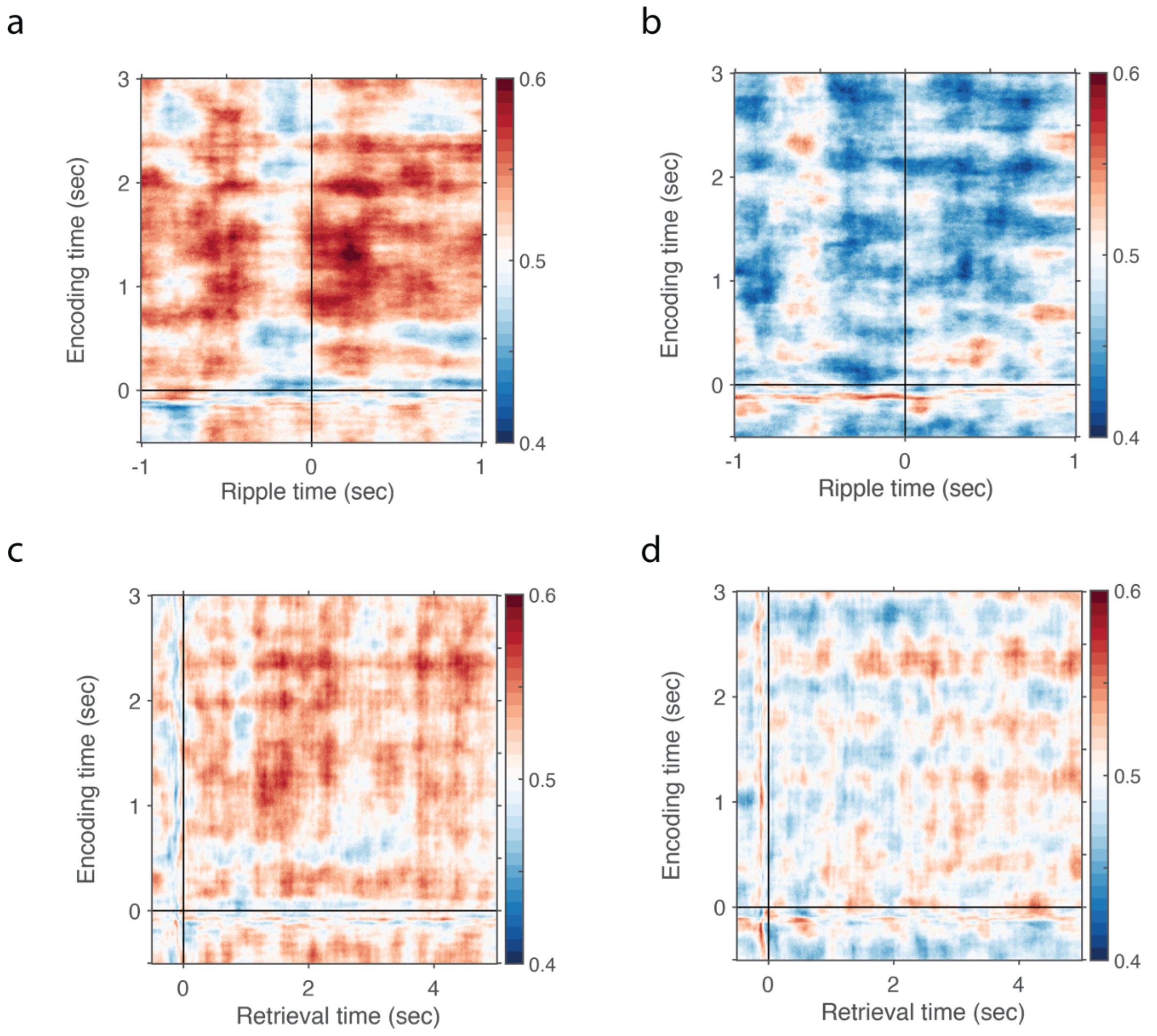

Supplementary Figure 3 | Reinstatement ripple-aligned and cue-aligned for AM+ and AM-, separately. **a** AM+ trials showed highest decoding accuracy following ripple events. **b** Chance-level decoding around ripple events for AM- trials. **c** When aligning the data to cue-onset at retrieval, we found the highest decoding accuracy around 1.5 to 2.5 seconds during encoding and retrieval. **d** Chance-level decoding during the entire retrieval period for AM- trials.

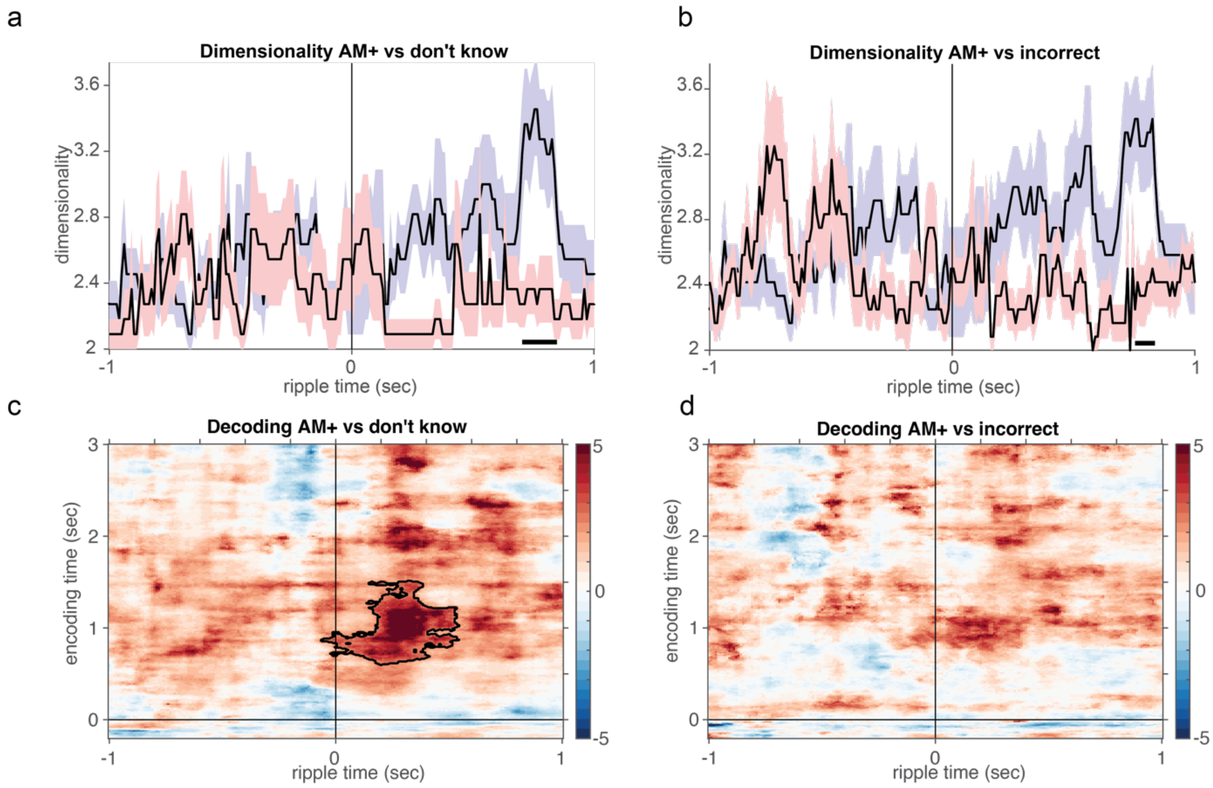

Supplementary Figure 4 | Dimensionality and reinstatement analyses dividing AM- into incorrect and don't know responses. **a** AM+ trials showed significant higher dimensionality as compared to don't know responses in a similar time window as main analysis in Fig. 3b. **b** AM+ trials showed significant higher dimensionality as compared to incorrect responses in a similar time window as main analysis in Fig. 3b. **c** AM+ trials showed significant higher decoding accuracy as compared to don't know responses in a similar time window as main analysis in Fig. 3a. **d** AM+ trials showed numerically higher decoding accuracy as compared to incorrect responses in a similar time window as main analysis in Fig. 3a. For all tests we used two-sided non-parametric cluster-based permutation test; $p < .05$, corrected for multiple comparisons.

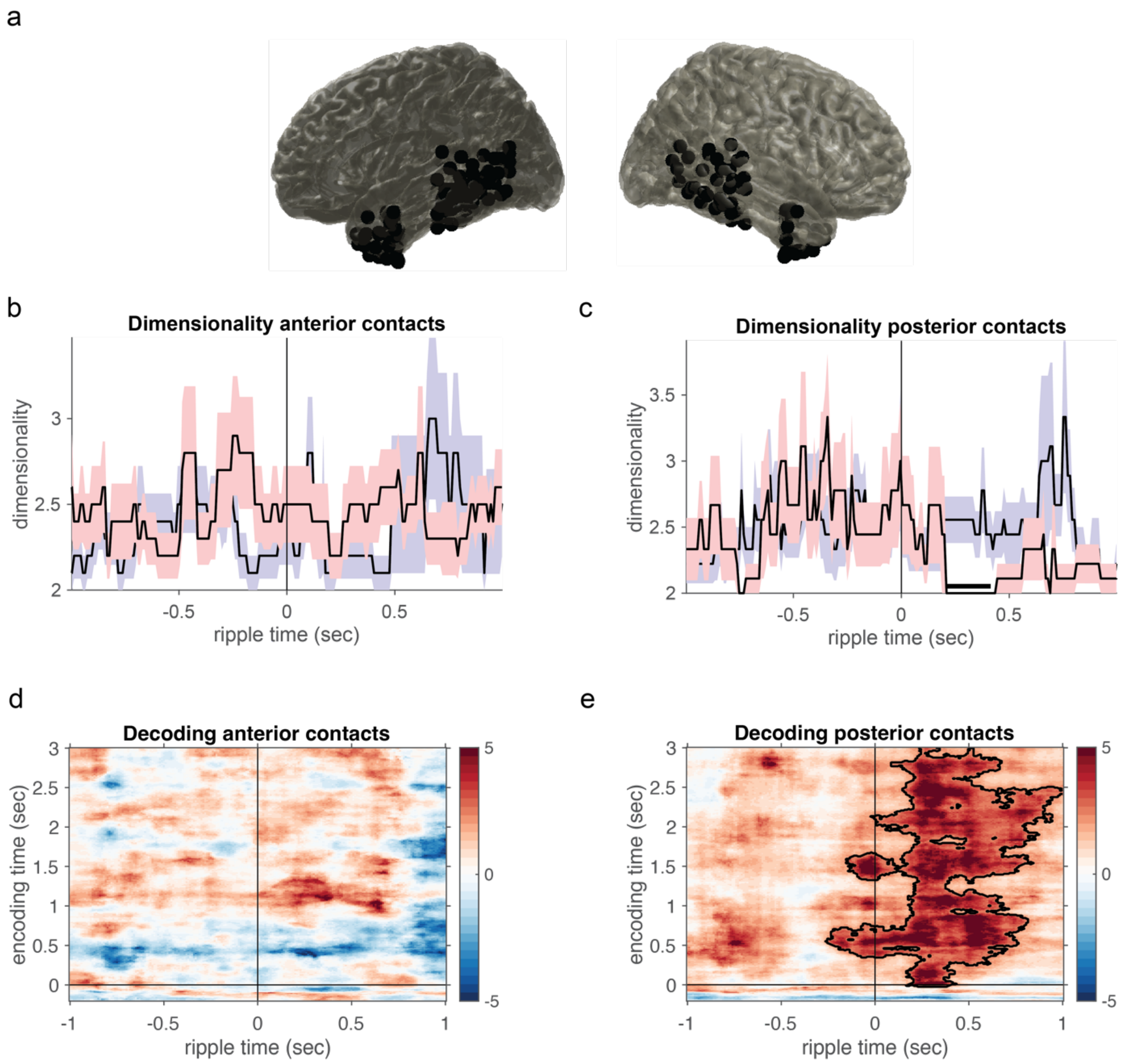

Supplementary Figure 5 | *Dimensionality and decoding analyses using anterior and posterior cortical contacts. (A)* Included anterior and posterior contacts. **(B)** No significant difference between AM+ and AM- for anterior contacts, but with the same increase around 600-800ms as in Fig 3a. **(C)** Significant difference between AM+ and AM- in a similar time window as in Fig. 3a. **(D)** No significant decoding difference between AM+ and AM- for anterior contacts, but with largest difference in the same post-ripple time window as in Fig. 3a. **(E)** Significant difference post ripple between AM+ and AM- for posterior contacts. For all tests we used two-sided non-parametric cluster-based permutation test; $p < .05$, corrected for multiple comparisons.

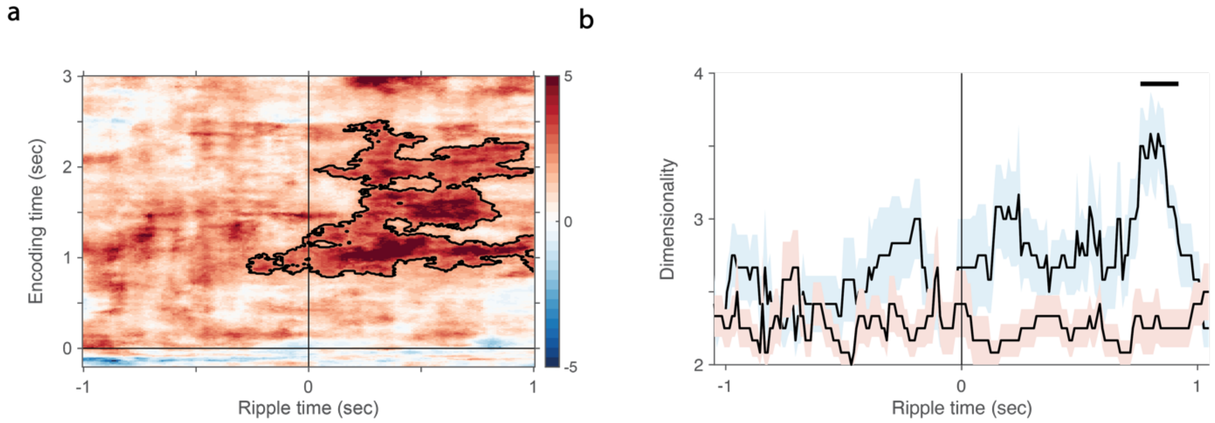

Supplementary Figure 6 | Spike-corrected reinstatement and dimensionality. a Reinstatement analysis excluding ripples occurring within ±500 ms of spikes in hippocampal channels revealed a similar significant cluster as in Fig. 3a. **b** The spike-corrected time-resolved dimensionality analysis showed a comparable significant cluster around 400–800 ms post-ripple, consistent with Fig. 3b. For all tests we used two-sided non-parametric cluster-based permutation test; $p < .05$, corrected for multiple comparisons.

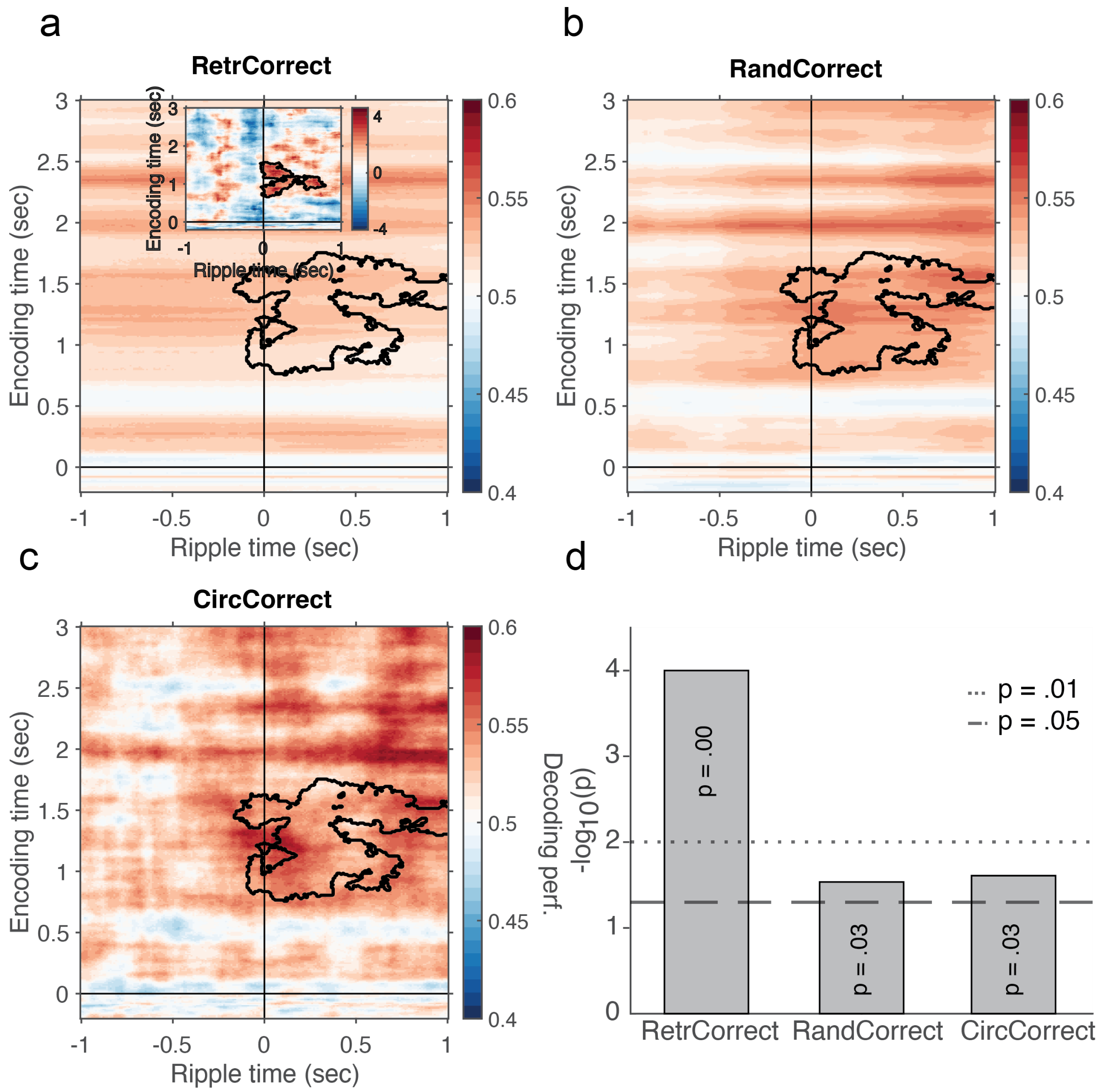

Supplementary Figure 7 | Control analyses confirming ripple-triggered reinstatement specificity. **(A)** From 1-5 seconds after cue onset, we picked 60 time points and treated them as ripple events and performed the same analysis as in 3a. The time generalisation matrix shows the decoding accuracy for these events with the significant cluster identified in Figure 3a highlighted in black. Inset shows a cluster-based permutation between the permuted baseline and empirical data, with a significant cluster in the same time window as in Figure 3a. **(B)** Decoding accuracy when AM+ trials were shuffled 1000 times. **(C)** A similar analysis was performed using a stricter control in which ripple times were swapped with those of adjacent trials (i.e., neighbouring trial shuffling). This revealed significant higher decoding in the empirical AM+ condition compared to the adjacent-trial control. **(D)** P-values when comparing empirical data with the different controls. For all tests we used one-sided non-parametric cluster-based permutation test; $p < .05$, corrected for multiple comparisons.

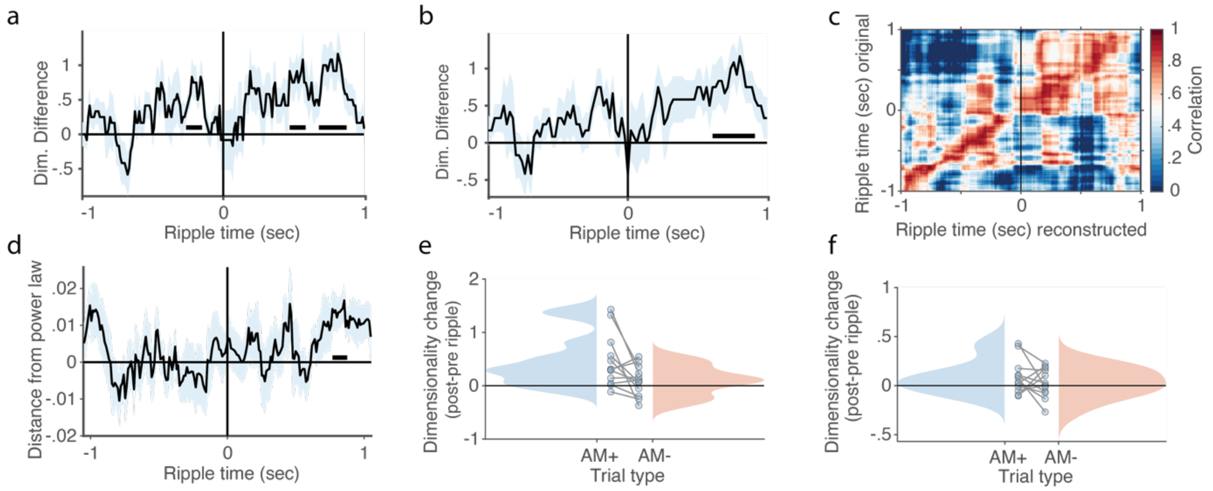

Supplementary Figure 8 | Robustness of dimensionality estimate, reconstruction analysis and alternative methods. a Using a 100 ms sliding window for dimensionality estimation produced results highly similar to those obtained with a 60 ms window, demonstrating the robustness of the approach. **b** same as **(a)** but with a 200 ms sliding window. **c** Correlation between original and reconstructed data, using the same number of components as in the PCA analysis, revealed a strong diagonal pattern, indicating a high-fidelity reconstruction of the original data structure. **d** Calculating the distance to a power-law distribution provided similar results as in 3b. **e** Estimating dimensionality using effective dimensionality provided similar results as in 3c. **f** Estimating dimensionality using Marčenko-Pastur threshold provided similar results as in 3c. For tests in (a, b, and d) we used two-sided non-parametric cluster-based permutation test; *p* < .05, corrected for multiple comparisons.

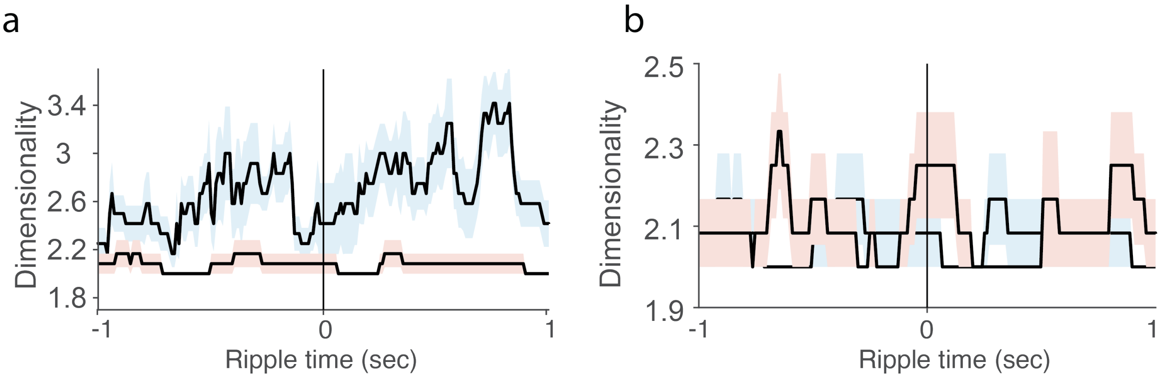

Supplementary Figure 9 | Dimensionality hippocampal vs. extra-hippocampal channels. **a** Extra-hippocampal channels (blue) had higher dimensionality as compared to hippocampal channels (red) for AM+ trials. **b** No difference between AM+ (blue) and AM- (red) trials in hippocampal channels. Note that, PCA on few channels in hippocampus will naturally not be very informative. For all tests we used two-sided non-parametric cluster-based permutation test; *p* < .05, corrected for multiple comparisons.

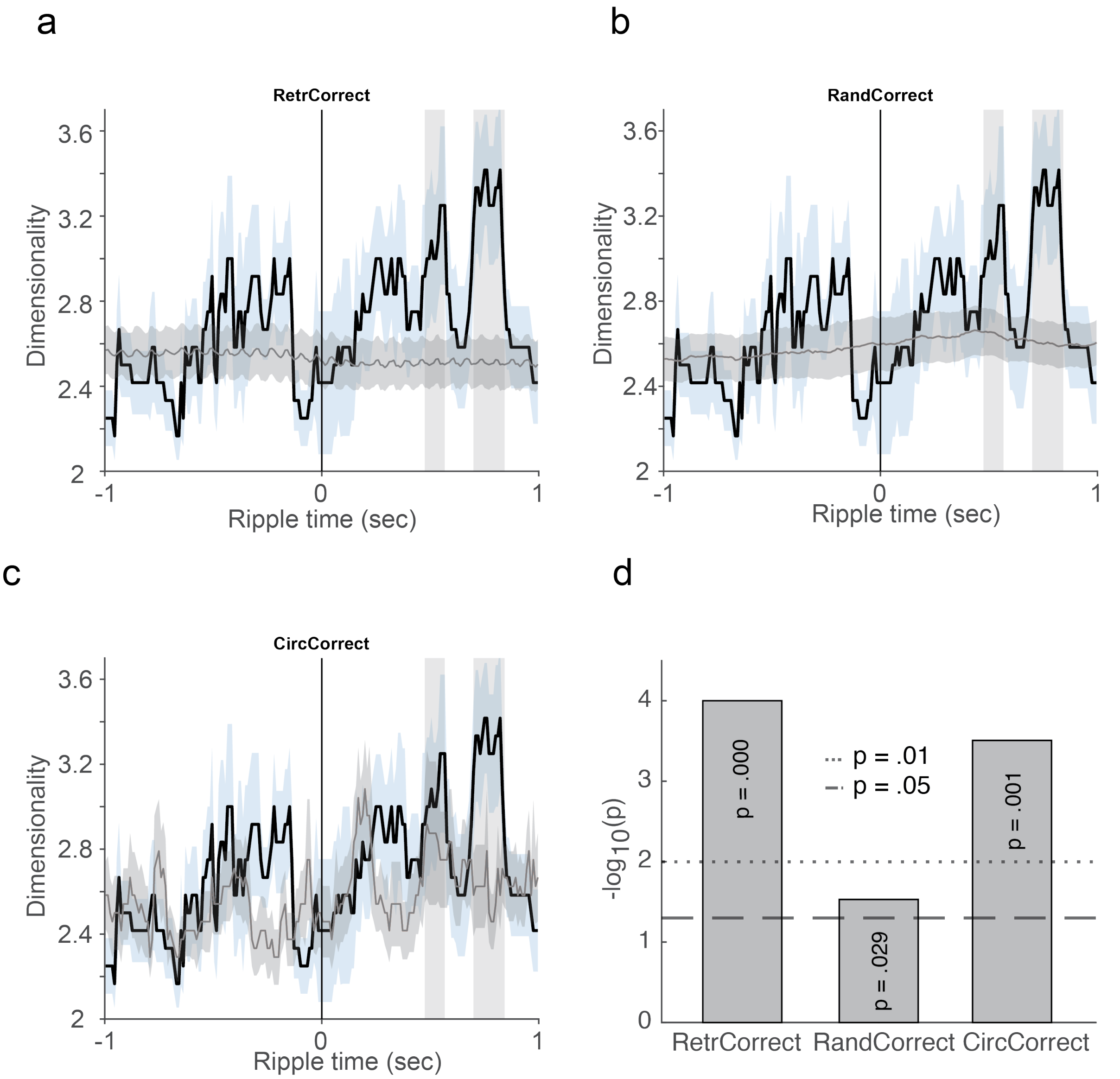

Supplementary Figure 10 | Control analyses confirming specificity of dimensionality expansion. a From 1-5 seconds after cue onset, we picked 60 time points for each participant and treated them as ripple events and performed the same analysis as in 3b. Grey is permuted baseline, blue is empirical data. **b** Shuffling AM+ trials 1000 times. Grey is permuted baseline; blue is empirical data. **c** Empirical AM+ (blue) and adjacent trials (i.e. temporally shifted controls, grey). **d** P-values when comparing empirical data with the different controls. For all tests we used one-sided non-parametric cluster-based permutation test; $p < .05$, corrected for multiple comparisons.

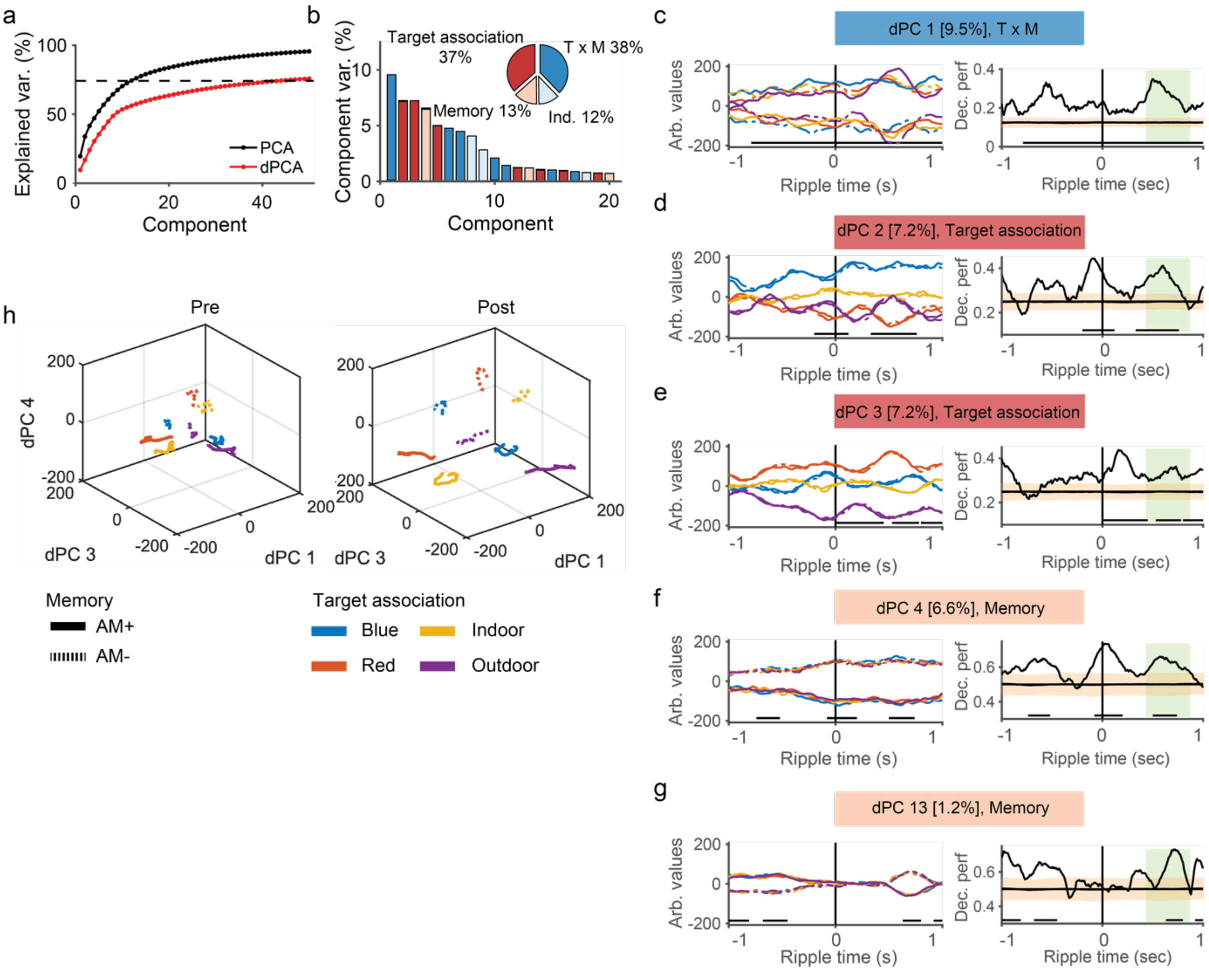

Supplementary Figure 11 | Demixed principal component analysis (dPCA). a Cumulative variance explained by the first 50 components exceeded the estimated signal variance (dashed line). Standard PCA (black) accounted for more total variance than dPCA. **b** The first four dPCs primarily reflected a mixture of "target association" and "memory" (dark blue), "target association only" (red), and "memory only" (beige). **c-g** Decoding accuracy for individual components, shown only for those with significant decoding between 400-800 ms post-ripple peak (highlighted by green bars). Left panels display component activation; right panels show decoding performance. Significant decoding intervals are marked with black horizontal lines. Orange shaded areas represent the shuffled distributions, with their mean indicated in black. **h** Neural state space plots of dPCs 1, 3, and 4 show that pre-ripple representations were more overlapping (left), whereas post-ripple activity exhibited greater separation between experimental conditions (right), indicating increased representational differentiation.

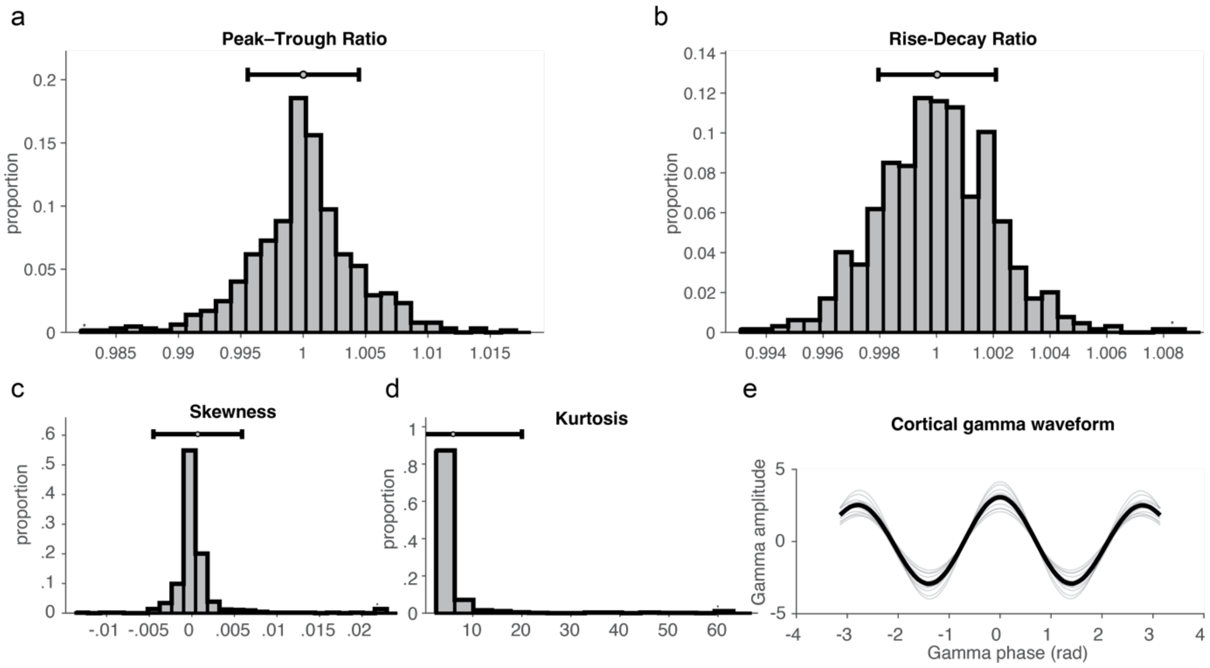

Supplementary Figure 12 | Gamma waveform-shape metrics. a Peak-trough sharpness ratio: ratio of mean peak sharpness to mean trough sharpness, reflecting symmetry of extrema. Values tightly clustered around 1 indicate near-perfect symmetry. **b** Rise-decay ratio: ratio of mean rise time (trough to peak) to mean decay time (peak to trough). Ratios near 1 indicate minimally asymmetric cycles. **c** Skewness of the gamma-band waveform, indexing asymmetry of the signal's amplitude distribution (values centred at zero indicate symmetric waveforms). **d** Kurtosis, reflecting the heaviness of the tails of the amplitude distribution. The distribution is consistent with narrowband-filtered gamma activity, and no systematic deviations were observed. **e** The average cortical gamma waveform.

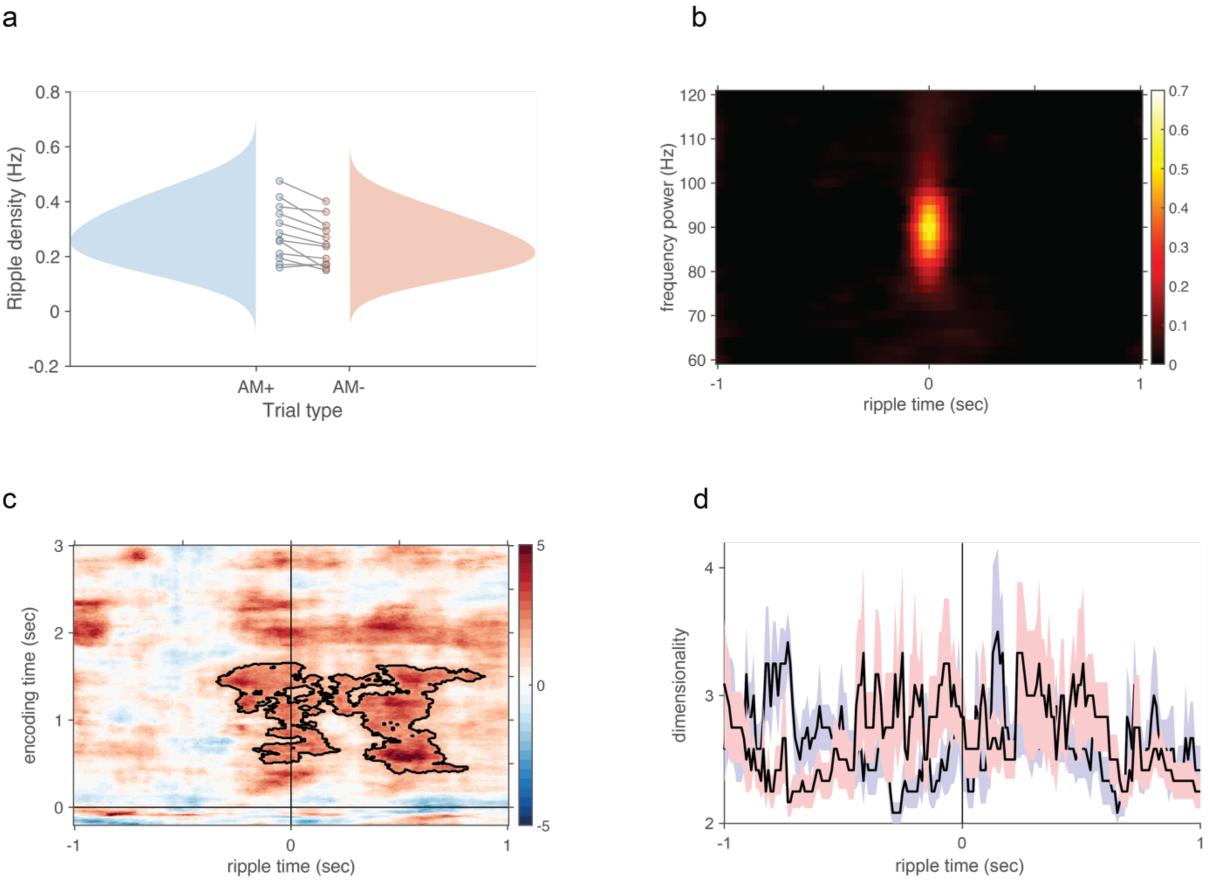

Supplementary Figure 13 | Ripple density, Spectral peak of ripples, Reinstatement and dimensionality for bipolar referencing. a Ripple density was higher for AM+ vs. AM- trials (paired-samples t-test). **b** Peak of ripples at 90Hz. **c** Cortical reinstatement analysis shows similar significant cluster as in Figure 3a (two-sided non-parametric cluster-based permutation test; $p < .05$, corrected for multiple comparisons)**. d** Time-resolved dimensionality analysis did not show the same significant cluster around 400-800ms post ripple, which is to be expected under bipolar reference scheme (two-sided non-parametric cluster-based permutation test; $p < .05$, corrected for multiple comparisons).

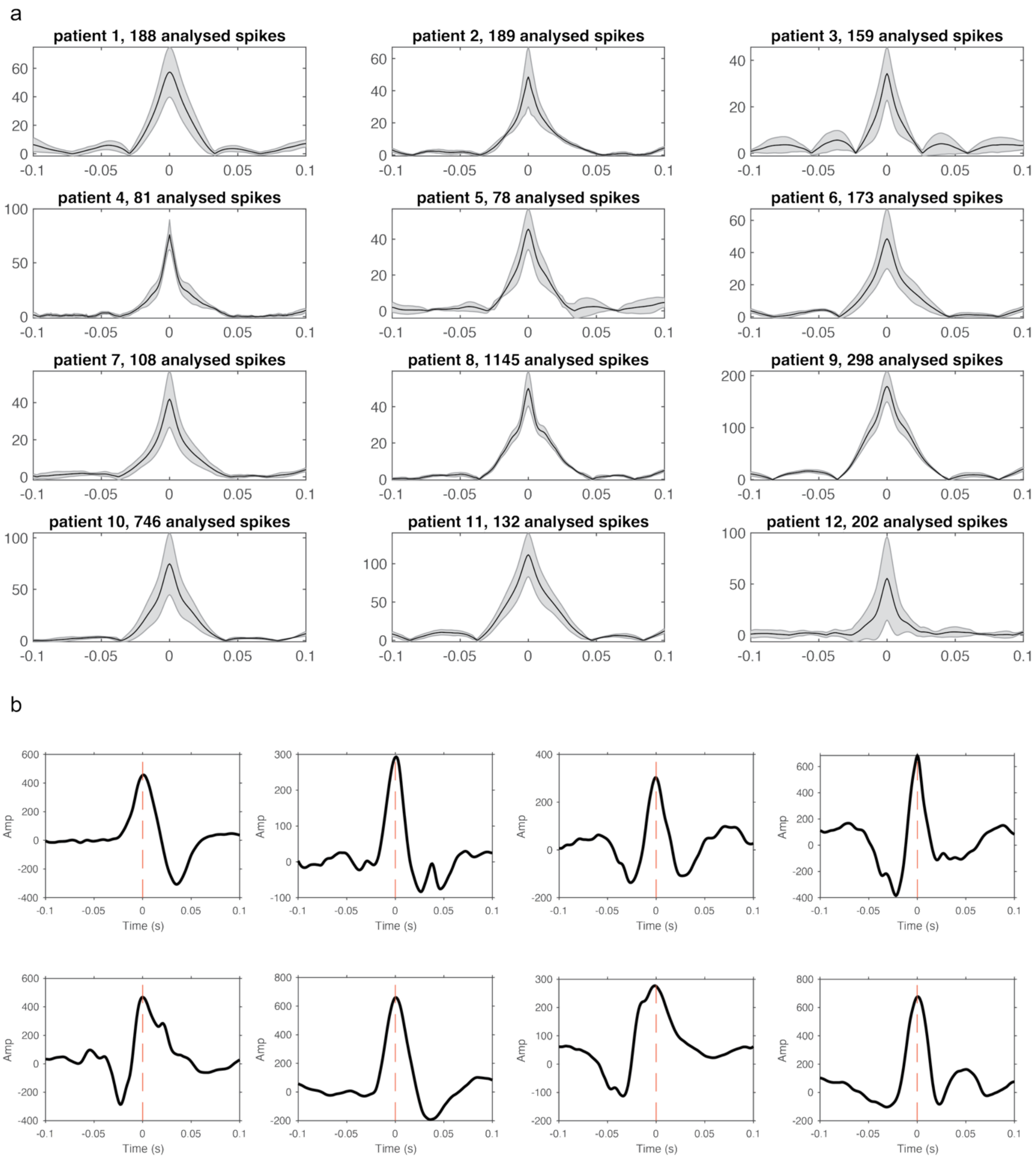

Supplementary Figure 14 | Spike-triggered averages. ***a****.* For each participant, we extracted and averaged spikes, with the number of spikes above each spike-triggered average. **b.** Example of extracted spikes.

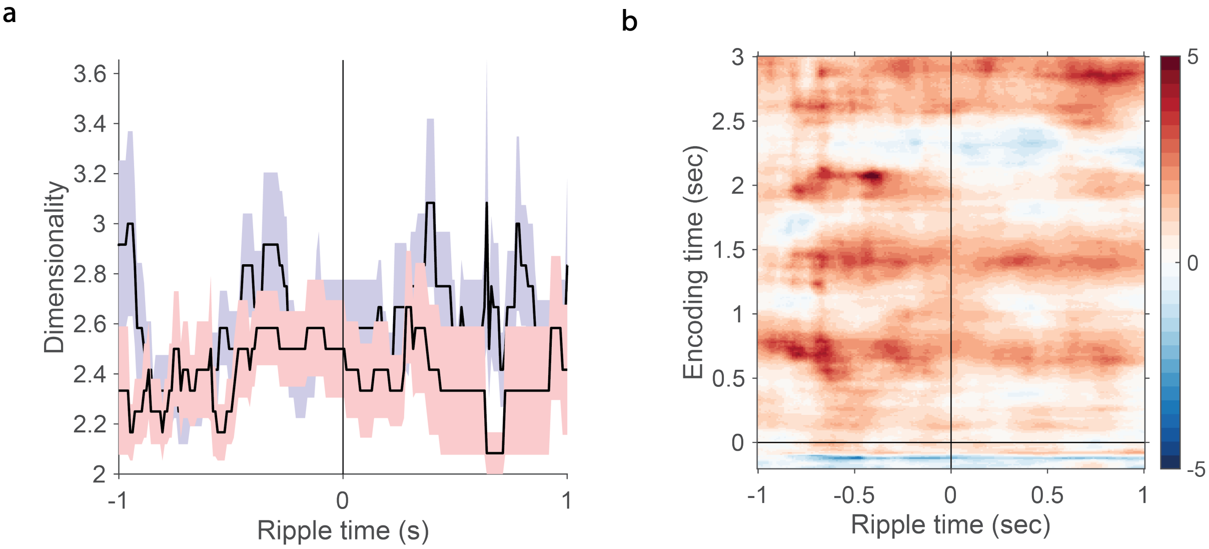

Supplementary Figure 15 | Dimensionality and decoding analyses based aligned on spikes. a Dimensionality analysis as in Fig. 3b showed no difference between AM+ (blue) and AM- trials (red). **b** Decoding analysis as in Fig. 3b showed no difference between AM+ and AM- trials. For all tests we used two-sided non-parametric cluster-based permutation test; $p < .05$, corrected for multiple comparisons.

Supplementary Table 1 | Characteristics per patient.

Number of analysed trials is for one ripple per trial.

Patient	Implantation scheme	Number of analysed HC contacts	Number of analysed extra-HC contacts	Number of ripples	Number of analysed AM+ trials	Number of analysed AM- trials
1	Longitudinal	8	28	563	51	63
2	Longitudinal	7	59	1098	34	89
3	Longitudinal	7	28	907	48	109
4	Longitudinal	6	70	563	81	27
5	Longitudinal	6	37	1088	144	45
6	Longitudinal	6	53	356	48	46
7	Longitudinal	8	24	832	35	41
8	Longitudinal	7	54	2048	44	115
9	Lateral	2	61	220	31	29
10	Lateral	3	59	441	59	63
11	Lateral	4	81	304	42	19
12	Lateral	10	93	1028	87	38

Supplementary Table 2 | Linear mixed-effects model of dimensionality transformation.

Name	Estimate	SE	t	df	p	95% CI (lower)	95% CI (upper)
Intercept	2.28	.07	32.47	19,988	<.001	2.14	2.42
Trial Type	.09	.03	2.82	19,988	.005	.03	.16
Half	.14	.03	4.39	19,988	<.001	.08	.21
Trial Type x Half	-.09	.02	-4.16	19,988	<.001	-.13	-.05
