# Supplementary material for: Hippocampal ripples initiate cortical dimensionality expansion for memory retrieval
Source: Nat Commun. 2026 Jul 20;17:6677. doi: 10.1038/s41467-026-75345-6 (PMC13385388; doi:10.1038/s41467-026-75345-6)
Supplement: Supplementary file 2 — Peer Review file [file 41467_2026_75345_MOESM2_ESM.pdf]

nature portfolio

Peer Review File

Hippocampal ripples initiate cortical dimensionality expansion for memory retrieval

Corresponding Author: Dr Casper Kerren

This file contains all reviewer reports in order by version, followed by all author rebuttals in order by version.

Version 0:

Reviewer comments:

Reviewer #1

(Remarks to the Author)
This manuscript explores an important question: do hippocampal ripples link pattern completion to cortical reinstatement? In this manuscript, Kerrén et al. analyze a previously collected dataset of 12 epilepsy patients performing an associative memory task in which participants encoded arbitrary word-image pairs, then were cued by the words to recall the associated images. As I understand it, the authors argue that during successful retrieval:
1. the prevalence of hippocampal ripples increases preceding memory responses
2. cortical reinstatement of stimulus identity uniquely follows the largest ripple
3. this cortical activity increases in dimensionality (n important PCs)
4. these dimensions reflect disentangled experimental variables
5. and this separation is enabled ripple-induced theta-gamma PAC in cortex
The authors take a unique approach in investigating the dynamics of reinstatement relative to ripple time. And I applaud the authors for this neat and intuitive story. But these are a lot of mechanistic assertions that ought to rely on careful and precise demonstration, and I find that almost every single step of this story requires significantly more support and validation. If the authors are able to do this, I think this work could contribute significantly to the field's understanding of ripples and memory in the human brain.

Major issues:
1. A few particularly finicky analyses are applied that require significantly more validation to support the author's conclusions:
a. Analysis of cortical reinstatement:
i. The basic method requires further validation and justification.
1. Grouping every cortical electrode suggests that the authors believe that every region of cortex sampled across their population is involved in reinstating the contents of a simple word-picture association in the same way, whereas much literature would suggest a slew of region-specific differences (e.g. PMID 39970226). The authors should justify this approach – perhaps through demonstrating that different cortical ROIs behave similarly. This is particularly important given the variance in cortical coverage across participants.
2. The relationship of cortical activity to ripples is
ii. There is insufficient detail to judge the reinstatement analysis.
1. I am not sure I understand the rationale for not performing cross-validation with a test in the encoding data. How do I know that the trained decoder is stable? A similar classifier-generalization analysis wa s used in a similar task by the authors' collaborators (from whom they received the data for this project), and cross-validation was performed in this case (PMID: 34880133).
2. Treating "don't know" responses as equivalent to incorrect responses assumes the neural signature of failed memory is the same for guesses and explicit failures. That assumption affects both decoding comparisons (AM+ vs AM–) and dimensionality calculations that later depend on trial labels. They did this for balancing, which I get, but they need to show the main effects survive excluding such trials.
b. PCA
i. The authors use a thresholded automatic artifact rejection process to get rid of putative interictal activity. Combined with shuffle-based controls, this might typically be sufficient for most analyses. But I am not convinced it's sufficient for PCA, where there might be a lot of sub-threshold interictal activity which can still explain a lot of variance in individual patients.
ii. On a related note, the clean correlation between reaction time and dimensionality is actually a little concerning to me.

Memory retrieval performance is often impaired due to interictal activity in contralateral to the seizure focus (which most of the sample hippocampi are; PMID 23685931). Can the authors verify that slow reaction times are not, in part, explained by prevalence of subthreshold IEDs, and that these, in turn, do not lead to poor ripple identification or systematically lower dimensionality estimates?
iii. Similarly, is dimensionality related to n electrodes? With n electrodes likely correlated with theorized epileptic spread, and thus potentially with RTs?
iv. How does the authors' unique referencing choice influence the PCA/dPCA results? A common average may lead to less local signal than, say, a bipolar reference. And I've never seen a trimmed common-average before; the implications are unclear for principal components analyses given that it removes electrodes which may contribute to large amounts of variance in a common average.
v. The use of time-resolved PCA is warrants further validation. With time windows that short (<=200 ms), are dimensionality estimates robust to noise?
c. PAC
i. Within-electrode PAC is already hard to identify; true long-distance PAC is even rarer and frankly I question the robustness of this finding. As the authors state, Figure 4 would represent the "the first evidence of hippocampal theta–cortical gamma phase–amplitude" and I think this warrants quite a bit more investigation to validated. So little has been done to make me trust this analysis that I find it a bit difficult to assess overall (it's not even clear how PAC was computed! Modulation index?)
1. I find the averaging across channel pairs across the entire cortical sampling here very obfuscating. For example, given that the authors align PAC to local peaks, what would it even mean for 5 Hz to coupled to 90 Hz in temporal cortex vs. 55 Hz in parietal cortex? Do the authors really think theta oscillations in the hippocampus propagate in the same way, and at the same time, across all of cortex? Even at the level of the hippocampus, theta power and frequency differs across the longitudinal axis– does it make sense to average across anterior and posterior hippocampal electrodes?
2. Does the trial-level shuffling (only 250 permutations) address concerns about gamma waveform shape contributions to PAC? Does gamma waveform shape differ in different regions or across different frequencies of gamma oscillation?
3. What is the relationship of ripple activity and theta power in the hippocampus? Theta power and memory?
ii. The authors cite two papers by Vaz et al., but ignores the most pertinent findings from that group involving cortical ripples coupling to MTL ripples. I find it hard to square their findings of ripple-induced cortical PAC with cortical gamma with the prevalence of cortical ripples and consider this a major gap in understanding the implications of this paper in relation to existing literature.
d. dPCA
i. I admittedly don't know too much about dPCA; can the authors provide citations for other works using dPCA with field potentials recorded from epileptic direct-brain recordings to further support their application here? If not, and given my qualms with the other methods here, I would request further empirical validation of the approach against potential pitfalls.
Across analyses:
2. The surrogate-based control analyses utilized here are unconvincing:
a. The statistical comparison between empirical and shuffled data seems very lenient. If I understand it correctly, the authors are conducting a paired t-test between the empirical decoding and the mean of the shuffles within each patient. Typically, one would be interested in whether the empirical data is systematically greater than 95% of the shuffled data within each patient.
a. The first shuffle method used (RandCorrect) also seems too liberal (it is easy to destroy classifier performance!), and yet the empirical data is still only barely better than the most liberal shuffle (RandCorrect, p=0.02), even given the lenient hypothesis testing mentioned above.
b. The second shuffle method used (CircCorrect) is more well-motivated but doesn't quite work (p=0.06). I think this could be because there seem to be only ~ 38 AM+ retrieval trials per person (if I am understanding the number of trials included correctly) – how many unique circular shuffles could there even be?
c. I would be more convinced by a control framework that really tests the central question more precisely. The central question is (as I understand): is the time following ripple onset special? For example, does the (largest) ripple's onset time t consistently predict reinstatement of feature-specific encoding activity better than alternative times during retrieval? So, a control analysis might test the decoding aligned to every other time in the trial, and then z-score ripple decoding compared to this population. Or, one could image performing the exact same analyses relative to IEDs rather than ripples!
3. Selecting the single largest ripple per trial does not seem that theoretically motivated, and potentially too arbitrary. Do the authors believe that reinstatement only occurs following the largest ripple? Do the authors believe that the chance of large ripples increases with ripple density? Does the size of the largest ripple systematically differ between AM+ and AM- trials, or with timepoint during retrieval (e.g do they tend to occur later)? How do all ripple qualities differ between AM+ and AM-, and how are these accounted for in comparing AM+ and AM- ripple-locked variables? For example, ripple length is the property associated with memory (PMID: 31197012).

Minor issues and clarifications:
1. Can the authors cite a source on their automatic artifact rejection approach?
2. How were false positive ripples identified for exclusion?
3. Why is ripple density a useful measure in the authors' analysis framework? They only end up using the largest ripple for subsequent analyses. Do they think cortical reinstatement is occurring relative to every ripple? Wouldn't that make it harder to resolve ripple-induced reinstatement in the authors' analysis pipeline when ripple density is higher?
4. It is not totally clear to me how many trials were performed by each participant.
5. Is it truly the case that encoding data is down-sampled and smoothed into low-frequency signal, but not the retrieval data? If so, is this meant to test compression of experience from encoding to retrieval? Can the authors clarify this point and better theoretically justify and/or empirically validate this analysis choice?
6. What happens to the scale of PAC in Figure 4C (bottom) and 4D? The caption also mislabels 4E as 4F.

(Remarks on code availability)

Reviewer #2

(Remarks to the Author)
In the manuscript, Kerren et al. present new data on the interplay between hippocampal and neocortical dynamics during an associative memory task. Using intracranial electroencephalography (iEEG) recordings from 12 human patients with pharmaco-resistant epilepsy, the authors report: (i) a higher occurrence of hippocampal ripples during successful compared to unsuccessful retrieval; (ii) at retrieval, a reinstatement of neocortical activity patterns that were present during encoding, time-locked to hippocampal ripples; (iii) greater effective dimensionality of neocortical representations following ripples during successful, but not unsuccessful, retrieval; and (iv) increased theta–gamma phase–amplitude coupling (TG-PAC) during retrieval, occurring after the ripple and preceding the dimensionality expansion. Taken together, the study proposes that neocortical pattern reinstatement is supported by hippocampal ripples, which trigger TG-PAC and subsequently expand “compressed” cortical representations to facilitate successful associative retrieval. While the results are potentially of interest, the manuscript requires further clarification and additional controls to warrant publication.

Throughout the paper, the “unsuccessful retrieval” condition (AM–) combines both incorrect responses and trials in which patients recognized the image but explicitly reported uncertainty about the associated item (“don’t know” responses, denoted “?”). Importantly, “don’t know” responses constitute 67.5% of all AM– trials, whereas only 32.5% correspond to truly incorrect responses. Thus, the study effectively compares successful associative retrieval against a heterogeneous group dominated by uncertainty, not purely by retrieval failure. This is critical because the authors’ main claim concerns processes specific to associative recognition memory rather than simple recognition, a particularly restrictive interpretation. Grouping “don’t know” trials with incorrect ones introduces potential confounds, as many of the reported effects (e.g., ripple rate, cortical dimensionality, TG-PAC) might reflect participants’ subjective certainty or uncertainty rather than accuracy per se. The authors attempt to justify this grouping (p.18, lines 25–26) by stating that “failure to recall is behaviourally equivalent to an incorrect memory judgement.” However, these two cases differ in perceived confidence: in the former, participants recognize the image but are uncertain of the association, while in the latter they are confidently wrong. A more detailed characterization of these subcategories is therefore necessary to strengthen the interpretation of the results.

The manuscript provides detailed information on hippocampal electrode localization but does not specify the placement of neocortical contacts. Are all extra-hippocampal electrodes located within the temporal lobe, or do they span multiple cortical regions? Moreover, are neocortical contact sites comparable across participants? Clarifying the degree of overlap in neocortical coverage, and whether these regions are areas known to support associative learning, would provide important context for interpreting the findings. It would also be informative to discuss whether the recorded neocortical sites are implicated in visual or semantic associative memory processes. Including a figure analogous to Fig. S1 to illustrate neocortical electrode locations would greatly enhance clarity.

The reported increase in ripple rate during AM+ trials may be confounded by differences in response times, as AM+ trials had faster responses on average (1.9 s vs. 2.1 s for AM–; p. 4, lines 7–8). Given that the mean ripple rate across patients during retrieval is reported to range from 0.1 to 0.4 Hz, a 2-second response window implies that some trials likely contained no ripples, many contained one, and only a few contained more than one. In cases with a single detected ripple, response time could therefore strongly bias the computed ripple rate within retrieval epochs. To control for this potential artifact, the authors could (i) repeat the analysis after subsampling AM+ and AM– trials to match response-time distributions, or (ii) report the average ripple count within response windows rather than the rate.

P7, L32-35: “higher post-ripple dimensionality during AM+ trials was associated with faster response times …. linking representational expansion to … behavioural … markers of successful retrieval.”. Is their a relationship between when the dimensionality expansion occurred in a trial, and the reaction time of that trial?

For the analysis in Fig 3A-C, how many retrieval trials contained >1 ripples? It is claimed in the methods that when this occurs, the ripple with maximum envelope is chosen. However, if all ripples instantiate cortical reinstatement, will the other ripple not contaminate the analysis? If there are few of these trials, can they be excluded?

While Figures S3-S5 greatly bolster the author’s argument, showing alternative measures of dimensionality would help
Page 7 lines 27-28: “Similar results were found using effective dimensionality”.
Page 19 lines 26-27: “Almost identical results were observed when calculating the distance to a fitted power-law distribution across time”.
These are stated but not shown. Please show this in a supplementary figure.

It should be further addressed why decoding performance from the components oscillates around ripple time (Fig 3D). While the manuscript primarily argues about the improved decoding 400-800ms after the ripple, there are periods of significance roughly every 0.5s in Figure 3D.

In addition, dPC1 displays symmetric decoding performance before and after the ripple (Figure S6). This oscillatory pattern suggests that the computed decoding performance may be modulated by a slow (~2 Hz) brain rhythm. If so, ripple timing could be systematically aligned to this ongoing rhythm, and the observed relationship between ripples and neocortical dimensionality might arise from this coupling rather than retrieval per se. Additional control analyses are therefore needed to

rule out this possibility.

In the same analysis, since the ripple tends to happen 1 second after the cue, and the dimensionality expansion 400-800ms after the ripple, does that not correspond to the time when the patient would have already or almost-already made their choice? Is the decodability of AM+ versus AM- in part due to the fact that the patient is very close to reacting at this point? Is choice time not factored into the analysis?

In demixed PCA, are components decomposed from different marginalizations guaranteed to be orthogonal in the original space, similar to components decomposed from the same marginalization (i.e the same matrix)? If not, the plot in 3E and S6H might be misleading.

In figure 3D, the dashed line shows the separability of clusters found via Kmeans, and the increase is suggestive of greater dimensionality. However, would it not be more appropriate to plot the separability (silhouette score) of clusters as defined and labelled by experimental variables, not Kmean clusters? Furthermore, it seems that some of the results of cluster separation are reported in the Methods (P22 L30-33), which is a bit odd, these should be in the result section and potentially shown on Figure 3.

Fig 4D would benefit from some clarification: is PAC after ripple time-averaged? Or is the maximum taken? Same for dimensionality; is only the 400-800ms range used for dimensionality, while all post-ripple time is used for PAC?

P16, lines10-12: Eight patients had electrodes along the longitudinal axis of the hippocampus, while four had lateral implants (via the temporal lobe), likely capturing more of the transverse axis. Where there any systematic differences between these groups (i.e. did the longitudinal implants detect more ripples? How did that affect reported ripple rate?). Do the results qualitatively hold if these groups are analyzed separately?

Minor:

In general, more participant-specific information, possibly in the form of supplementary tables, would be helpful. For examples:
- P16 lines 28-32: a total of 72 hippocampal contacts across patients, and 647 extra-hippocampal contacts also across patients. But how many per-patient? How uniform or otherwise is the distribution ?
- P3, line22 - page4, line8: AM+ and AM- numbers (and their breakdown) for different patients should be reported, as opposed to the average across patients.
- Figure S1: A comment on why the number of detected ripples in different patients is very variable, from ~400 events in some patients to >2,000 events in others. Possibly, knowing number of hippocampal contacts per patient would help.

P8, Ll33-36: Incorrect reference to Figure 4A

Labelling axes of Fig4B as theta and gamma would be helpful. Currently it must be inferred from the range of frequencies plotted.

Since the dPCA decoding is an important part of the analysis In Fig3 and S6, a little clarification on how dpca_classificationAccuracy works in the methods would be useful. It is unclear to readers not familiar with the package.

(Remarks on code availability)
no documentation and no code as far as I can tell, just data mat files.

Reviewer #3

(Remarks to the Author)
The authors of this manuscript showed that cortical dimensionality increases immediately (<1s) after hippocampal ripples during memory retrieval in human subjects. After hippocampal ripples, but just before cortical dimensionality expansion, phase–amplitude coupling between hippocampal theta and cortical gamma emerges. While their findings are potentially interesting, I have several issues that should be addressed before the manuscript can be adequately evaluated.

1) For PCA-based dimensionality estimation, the authors determine the dimensions by identifying the “elbow” point at which the explained variance sharply declined across subsequent components. Is this method valid and used or supported by the literature? A more standard and widely used method to extract a statistically meaningful number of components identified by PCA is to determine the number of eigenvalues that exceed the Marchenko–Pastur threshold (Peyrache et al., 2009, Nat Neurosci 12:919–926). Since dimensionality estimation analysis is the basis of this manuscript, I suggest using this method to determine whether it and your method lead to the same conclusion.

2) Hippocampal sharp-wave ripples occur during non-theta states and rarely occur concurrently with theta oscillations, at least in rodents (Buzsaki, 2015). The authors claimed that, immediately after ripples in the hippocampus, phase–amplitude coupling between hippocampal theta and cortical gamma emerges. Does this mean that ripples and theta oscillations occur concurrently in the human hippocampus? Or, does the brain state change immediately after ripples, entering theta-states? Alternatively, are the hippocampal ripples reported in this manuscript not bona fide sharp-wave ripples, but could they be

gamma oscillations nested in theta oscillations in the hippocampus?

3) Which cortical areas were the iEEGs obtained from for cortical dimensionality estimation and analysis of hippocampal theta and cortical gamma phase–amplitude coupling?

4) In summary, the authors claimed, “Ripple events also marked the separation of task-relevant variables in cortical state space,” however, I could not find the analysis and figures that support this claim. Please clarify.

5) Fig 3E, “Plotting dPCs 1, 2 and 4, showed overlapping representations of experimental variables before ripples (left), whereas their separability increased after ripples (right).” If dPCA was performed on the pre- and post-ripple events together and the results were shown separately using the same cPC1, 2, and 4 coordinates, then the results would be more interpretable. Otherwise, it is difficult to interpret the comparison between the pre- and post-ripple events.

6) How many contacts (recording sites) were used to estimate cortical dimensionality? The number of recording sites affects the number of estimated dimensions, which can make them difficult to interpret. This point should be discussed.

7) page 7, lines 27-28: “Similar effect were found using effective dimensionality”. Since estimating dimensionality is probably the most important aspect of this manuscript, I suggest presenting the results of effective dimensionality in supplemental figures.

8) page 19, lines 26-27 “Almost identical results were observed when calculating the distance to a fitted power-law distribution across time.” I could not understand this sentence or its relationship to the other sentences in the paragraph. Please clarify.

9) page 19, lines 36-37 “In a follow-up analysis, we correlated the significant time points of dimensionality estimates with (1) …” It is unclear to me how the significant time points were identified. Please clarify.

10) page 20 lines 14-36, page 21 line 1-3. This section is difficult to understand and would benefit from the addition of explanatory figures.

11) page 29, lines 4-6 “(B) The first four dPCs primarily reflected a mixture of “target association” and “memory” (dark blue), “target association only” (red), and “memory only” (beige).” It is unclear how the authors determined which dPCs associated with "target association," "memory," "target association only," and "memory only." Please clarify.

(Remarks on code availability)

Reviewer #4

(Remarks to the Author)
The authors present results from intracranial recordings suggesting that ripples play a crucial role in memory reactivation in humans. They analyse previously published data (Staresina et al., 2012, 2016) to show that following ripples: (i) memory decoding increases during successful retrieval, (ii) the dimensionality of cortical intracranial EEG rises during successful retrieval, and (iii) theta–gamma coupling increases, unrelated to memory.
The combination of dimensionality analyses with ripples is both innovative and intriguing. Unfortunately, in many instances, methodological details are insufficiently described or remain opaque, and several control analyses are unconvincing. The authors also tend to overstate some interpretations, employing causal language where it is not warranted.
I have outlined my principal concerns and other points below. The most pressing issue is that it remains unclear whether the detected ripples are physiological in nature or merely artefacts.

Major concerns:
A) Ripple detection is critical, yet it is uncertain whether the reported ripples reflect genuine physiological events, and whether the detection methods can be trusted.

i) If these are indeed physiological ripple oscillations, one would expect to see a narrow-band peak in the power spectrum. Rather than relying solely on time–frequency plots—which can be biased by the authors’ filtering approach—the authors should present non–time-resolved power spectra based on the retrieval data. Specifically, they should compute a simple Fourier transform over 0–3 s and display the group average in Figure 2, as well as individual patient spectra in Figure S1. Sometimes removing 1/f helps to find peaks in higher frequencies.

ii) Merely citing literature instead of describing ripple detection methods in detail is inadequate for such a delicate and central issue. Moreover, two of the three referenced papers are not even concerned with ripples. Please provide the details of the ripple detection approach.
Typically, ripple detection involves thresholding the root mean square signal, yet this is not reported. Were the authors not using this criterion? Given their description—bandpass between 80–120 Hz, 3 cycles, 38–500 ms—the detected “ripples” could represent many things, but not necessarily ripples.

iii) Furthermore, the authors report power peaks around 60 Hz in their CFC analyses. How can they be confident that the ripples are narrowband, given multiple other gamma-range power peaks?

B. Unclear contribution of epileptic activity to “ripples”
The authors claim to use “an appropriate algorithm capable of detecting epileptogenic spikes” but provide no details. This is not sufficient. They must specify the algorithm, parameter settings, and confirm (ideally via visual inspection by a trained neurologist) that it performed appropriately on the individual patient level. They should also report how much data were classified as epileptic artefacts. Analysing only non-pathological hemispheres does not address the issue, since interictal activity often occurs outside the epileptogenic zone.
To substantiate their findings, the authors should detect epileptic spikes during relevant periods using an automatic method similar to their ripple detection, then repeat their main analyses using those spikes. They should report:
- Epileptic spike–triggered averages
- Memory contrasts of spike density (cf. Fig. 2)
- Reactivation analyses based on spikes (cf. Fig. 3A)
- PCA analyses based on spikes (cf. Fig. 3B,C)
These results should appear in the supplementary material incl. figures, so readers can properly assess the main findings.

C. Control analyses are not meaningful.
The authors’ approach attempts to control for significant effects during a specific time window, yet they never explicitly test for time windows. Instead, what their statistics in the main part do is to indicate whether two conditions are different – but not when exactly (see Maris & Oostenveld, 2007, J. Neurosci. Methods).
For instance, Fig. S5 “... when averaging across the significant time points identified in Fig. 2B…” is problematic, as it overinterprets a temporal cluster from the permutation test of the main analysis. Proper control would allow clusters without constraining time in the shuffled data, and then assess whether the occurrence of a significant cluster in shuffled data is equally probable.
The same issue applies to results presented in Fig. S2 (“…decoding accuracy within the significant cluster identified in Fig. 2A…”). Instead, they should test condition differences in their control analyses without temporal constraints, mirroring the main analyses. They should also compare shuffled AM+ and AM– data to determine whether their observed differences could be found by chance.

Major points
1) How can the authors ensure that reactivation is not driven by the similarity between reading the identical word at encoding and retrieval? Patients’ attention can fluctuate drastically in the hospital setting, which could lead to reading in one trial, but not reading in another (“forgetting”). Reading elicits stereotypical cortical activity. As Fig. 1b suggests, electrodes cover lateral temporal and frontal regions—likely language areas—whose timing could plausibly match the observed reactivation patterns (~500 ms post onset). A similar argument could be constructed regarding the dimensionality analyses based on this contrast.

2) The reactivation in Fig. 3A spans 0–1 s after ripple onset and shows elevated t-values throughout encoding, also pre-ripple. The authors provide a mask that outlines the cluster from the permutation statistics, but this is not a good indicator for when reactivation actually occurs. They must present unmasked grand-average data to reveal temporal peaks, and test whether ripple-locking is indeed critical by repeating decoding on stimulus-locked data.
From the brief description in the results, it is very unclear whether the reactivation has any temporal relationship with the occurrence of ripples.

3) The claim that dimensionality increases after ripples is overstated; their statistics merely indicate a difference between conditions, not its exact timing. Concluding that dimensionality changed only after ripples and not before is incorrect. Fig S3A even shows a pre-ripple difference that goes unmentioned.

4) The PAC analyses are overinterpreted; PAC does not demonstrate inter-areal communication (see Schneider et al, 2021, Neuron, for a related discussion).
(Gamma) Oscillations: no justification provided that this is an actual oscillation. Frequency bands are defined arbitrarily rather than empirically. If anything, the plot in 6A shows that this is broadband, rather than narrow band activity. The latter would, however, be a prerequisite for an oscillation.
The choice of time window (–4 to 6 s) for PAC is also puzzling; extending by 1 s each side should have sufficed. Details on whether PAC analyses were limited to retrieval are missing.
Another question that comes to mind here is why the authors did not look at coupled ripples in the cortical electrodes (see Vaz et al.) and how this might relate to any of their theta gamma findings? This could strengthened their point about neuronal communication.
Fig 6B shows that close to the peak power (for both gamma and theta), there is higher PAC. Seems trivial, given the authors chose the data in a way that they would have the highest signal to noise ratio at this particular point.
Why are there no memory contrasts for PAC, as for LDA and PCA analyses?
Fig 3A: It seems there are 9 data points for gamma, and 3 for theta; why?

5) Do the main results hold when AM- items are split into wrong vs. no memory and then separately compared to AM+ items?

6) Using a common average reference across all electrodes eans that all electrode will share some variance, also hippocampal and cortical electrodes. Separate references for each would be more appropriate, given the framing of these as

distinct groups.

7) Statements such as “hippocampal ripples initiate a process…” are unwarranted; temporal precedence alone does not imply causality. Please check carefully whenever causality is implied.

8) Figure scales, colour schemes are sometimes confusing and may mislead readers.
-The scale in Fig S2B is decoding accuracy, while the scale in Fig 3A is t values, making it impossible to compare them. The authors need to provide a version of Fig 3A using the same scale as in Fig S1A and B.
-The use of the color scheme in FS2B is misleading. While in many other plots white indicates zero values, white indicates intermediate values. This gives the impression as if decoding accuracy would be very low within the boundaries of the outline when it is not.
Overall, this way of presenting the data may leave readers less confident in the results.

9) Questions on the dimensionality analysis:
-The choice / number of components for the dimensionality analysis seems arbitrary (page 8). Why these four and not others or more or less components?
-Fig 3 shows elevated dimensionality is prior to ripple onset. Why would that be?
-The cluster based statistics tests for differences between groups/conditions, but the temporal domain cannot be interpreted in the way the authors do it here.
-The authors should repeat their main dimensionality using the shuffled data in FS2A and B, separately.

Minor
-Key papers are neglected:
Axmacher et al 2008 Brain, Dickey et al 2022 PNAS, Sakon& Kahana 2022 PNAS,
Kunz et al 2024 Nat Neurosci; and others
These and other related papers should be discussed in the light of the present findings.

- The authors should compare ripple density between unsuccessful vs. successful recognized items.

-50% correct associative retrieval may indicate guessing behavior, given two alternatives. How do the authors control for this in their data and how would that affect their EEG analyses?

- the authors interpretation that the supplementary analyses “confirms” that “reinstatement was specific to the ripple latency of the current trial” is not correct. The non-significant difference in Fig S2B confirms – if anything – that there is no (significant) difference, but offers no insight on the timing.

- Why employ a linear mixed-effects model for pre–post ripple comparisons, when previous contrasts relied on simple tests?

-Why is the amplitude in Fig 1a increasing before ripple onset?

-Figure S1 shows that bilateral hippocampus electrodes for patient 12, but the authors state that only one hemisphere would be included in the analyse. In case this is not an error in the figure, the authors need to repeat all group level analyses.

-Page 5 Line 29: the authors do not show replay

-Selective interpretation of how the authors’ own findings align:
Authors don’t say that cortical reactivation was immediately following ripples, without delay, which is not in line with Michelmann et al. A less opaque discussion would be more interesting.

-Include a reference from animal literature about replay compressions

-Figure 1a, legend: What about unsuccessful recognition?

-Figure S4B: plotting problem?

(Remarks on code availability)

Version 1:

Reviewer comments:

Reviewer #1

(Remarks to the Author)
The authors have done a very rigorous job following up on my many questions. I generally believe the manuscript is now a detailed, valuable resource for readers despite some minor remaining concerns.
1. The authors addressed my comment by splitting their cortical contacts into anterior vs. posterior temporal cortex and re-running the primary analyses from Figure 3A-B. The authors seem to want to elide the fact that the posterior contacts might drive their significant observations, but I don't think this weakens the overall conclusions and the additional anatomical information is perhaps interesting to include and discuss for the reader as a supplementary figure. Particularly in relation to PMID 39970226 which is such a complementary work to this.
2. The authors do a great job of identifying putative interictal spikes and demonstrating that spike-aligned analyses do not replicate their ripple-aligned results. But I still think it is important to address a couple of remaining questions:
a. Why are the degrees of freedom for the t-test between AM+ and AM- spike counts only 11 (subject level) when there are 72 hippocampal contacts and hundreds of temporal contacts? Both regions are well known to show higher IED counts during poor memory encoding (hence my original concern).
b. Even if spike counts are the same across AM+ and AM-, it is odd to me that the dimensionality doesn't seem to change at all in both conditions then around the time of interictal spikes in the new supplementary plot. How do the authors interpret this? I would expect the variance to collapse to 1-2 dimensions around interictal activity to reflect the presumably synchronized activity across multiple electrodes.
3. I appreciate the authors' answer re: bipolar referencing not making sense for such an analysis but I still don't have a firm answer for why a trimmed common-average reference is appropriate. It is one thing to remove outlier channels entirely because you suspect they are artifactual or noisy – that is fine. But that doesn't seem like the logic applied to this trimmed common average and it would be nice to see firmer justification or some assessment of the impact it has on the results.

(Remarks on code availability)

Reviewer #2

(Remarks to the Author)
I thank the authors for carefully addressing my comments. I only have one comment regarding the code (see below). Other than that, the paper is suited for publication IMO.

(Remarks on code availability)
In most script, path to data and other packages is hard coded, this makes it tedious for users to adapt the code. Specifically, a line like
settings.base_path_castle = '/Users/kerrenadmin/Desktop/Other_projects/Dimensionality_ripples_Casper_and_Bernhard/';

should be:
settings.base_path_castle = [path_to_packages filesep path_to_CasperBernhard];

with path_to_packagee, path_to_CasperBernhard and other data and third party software properly defined in a proper configuration file.

Reviewer #3

(Remarks to the Author)
The authors carefully addressed my comments. I have no further remarks, and I believe that the article is suitable for publication.

(Remarks on code availability)

Reviewer #4

(Remarks to the Author)
The authors have addressed all points and provided additional information on their approaches; however, critical issues still persist. Unfortunately, these issues are likely to substantially affect the main results; therefore, they need to be resolved before the remainder of the results can be meaningfully evaluated.

Major Concern A:
The employed ripple detection algorithm is problematic:
-1.5 standard deviations as threshold for ripple detection is unusually low, below common practice. With such a low threshold, the likelihood of false positive events will be increased (see Liu et al., 2022, consensus statement);
Given the current debate in the field, adapting a more conservative approach to ripple detection is highly recommended. The authors should repeat their analyses with a detection threshold of mean plus 3 standard deviations.

Ai) the authors might have misunderstood this point:
“present non-time-resolved power spectra based on the retrieval data. Specifically, they should compute a simple Fourier transform over 0-3 s and display the group average in Figure 2, as well as individual patient spectra in Figure S1.”

The request was to show a power spectrum not centered on ripples, but instead over the whole retrieval period from 0 to 3 sec, during which ripples are assumed to occur. If there is a substantial amount of genuine ripple oscillations in the data, this should show as a peak in the power spectrum, independent of prior ripple detection.

Instead, the authors chose to center their data on detected ripple events, then compute a power spectrum; this approach was already used to create ripple centered TFRs. However, the outcome of this approach might be biased by centering data around transient sharp increases that might have passed the detection threshold. The resulting ripple centered TFR might look like a narrow band increase in power at ripple frequency, but cannot be distinguished from a smoothed, averaged broad band increase induced by transients.

B)
-the proposed handling of potential epileptic activity is not ideal: instead of interpolating the spikes which can create problems in the data, a more conservative approach would be to exclude epochs around epileptic activity (+/- 1 second) and repeat the analyses.
-The depicted spike triggered averages do not resemble what is usually found when interictal epileptic activity is averaged: a spike-wave complex, which usually (but not always) shows in the erp. It seems the authors averaged across all detected spikes on all channels, without adjusting for polarity, which might explain the difference. Increasing the time around the spike might also help here.
A central concern for this manuscript is that IEDs themselves can drive hippocampus-to-cortex coupling, as demonstrated by Gelinas et al., 2016, Nature Medicine. This raises the possibility that the reported coupling effects may, at least in part, reflect IED-related dynamics rather than genuine ripple-driven interactions. The liberal ripple detection criteria (see above), together with uncertainties regarding IED detection and control, substantially weaken confidence in the present approach. Gelinas et al 2016 and its implication for the findings presented here should also be thoroughly discussed in the manuscript.

Question on sufficient trial numbers:
multiple reviewers asked about don’t know and incorrect responses in AM-. Since trial numbers in AM- can be as low as 19 (table S1), how many trials are left for truly incorrect reponses, which make only 32.5% of AM- trials? And is that number (6 trials in patient 11?) sufficient to have stable estimates in each of the patients? When contrasting, are trial numbers adjusted across conditions to account for more variance in low trial number conditions?

(Remarks on code availability)

Version 2:

Reviewer comments:

Reviewer #1

(Remarks to the Author)
The authors have sufficiently addressed my concerns.

(Remarks on code availability)

Reviewer #4

(Remarks to the Author)
The authors have addressed all of my queries. I have no further comments.

(Remarks on code availability)

Open Access This Peer Review File is licensed under a Creative Commons Attribution 4.0 International License, which permits use, sharing, adaptation, distribution and reproduction in any medium or format, as long as you give appropriate credit to the original author(s) and the source, provide a link to the Creative Commons license, and indicate if changes were made.
In cases where reviewers are anonymous, credit should be given to 'Anonymous Referee' and the source.
The images or other third party material in this Peer Review File are included in the article's Creative Commons license, unless indicated otherwise in a credit line to the material. If material is not included in the article's Creative Commons license and your intended use is not permitted by statutory regulation or exceeds the permitted use, you will need to obtain permission directly from the copyright holder.
To view a copy of this license, visit https://creativecommons.org/licenses/by/4.0/

Summary

We thank the reviewers for their thoughtful and constructive feedback, which has helped us to clarify the manuscript and strengthen several aspects of the analysis and interpretation. Across the reviews, a number of common themes emerged. Reviewers encouraged us to provide further validation of key analytical approaches, together with clearer methodological descriptions, additional control analyses, and complementary analysis pipelines. A second recurring theme concerned interpretational specificity, particularly the heterogeneity of the AM- condition, the functional role of ripple timing, and whether the reported effects might reflect reaction time, subjective uncertainty, slow oscillatory structure, or epileptiform activity rather than associative memory retrieval itself. Reviewers also highlighted the importance of addressing spatial and sampling considerations, including variability in cortical electrode coverage, referencing schemes, ROI definitions, and the implications of aggregating signals across distributed cortical regions. In addition, several comments focused on robustness and statistical control procedures, motivating expanded surrogate frameworks, spike-aligned control analyses, reaction-time-matched comparisons, alternative dimensionality metrics, and cross-validated decoding at encoding. Finally, reviewers requested clearer physiological framing, particularly regarding the relationship between hippocampal ripples, theta activity and cortical high-frequency dynamics.

In response, we have implemented a comprehensive set of additional analyses and clarifications throughout the manuscript. These include ROI-specific analyses, cross-validated controls, separation of incorrect and "don't know" trials, spike-aligned control analyses, alternative referencing and dimensionality approaches, additional surrogate frameworks testing ripple-specific timing, further PAC validation and waveform controls, and more detailed reporting of electrode coverage and trial structure. Together, these additions directly address concerns regarding robustness, interpretability, and methodological transparency, while strengthening the overall conclusion that ripple-locked cortical dynamics reflect structured, retrieval-related processes rather than artefactual or non-specific effects. Below the reviewers can find our response to each of their respective questions.

REVIEWER COMMENTS

Reviewer #1 (Remarks to the Author):

This manuscript explores an important question: do hippocampal ripples link pattern completion to cortical reinstatement? In this manuscript, Kerrén et al. analyze a previously collected dataset of 12 epilepsy patients performing an associative memory task in which participants encoded arbitrary word-image pairs, then were cued by the words to recall the associated images. As I understand it, the authors argue that during successful retrieval:
1. the prevalence of hippocampal ripples increases preceding memory responses
2. cortical reinstatement of stimulus identity uniquely follows the largest ripple
3. this cortical activity increases in dimensionality (n important PCs)
4. these dimensions reflect disentangled experimental variables
5. and this separation is enabled ripple-induced theta-gamma PAC in cortex
The authors take a unique approach in investigating the dynamics of reinstatement relative to ripple time. And I applaud the authors for this neat and intuitive story. But these are a lot of mechanistic assertions that ought to rely on careful and precise demonstration, and I find that almost every single step of this story requires significantly more support and validation. If the authors are able to do this, I think this work could contribute significantly to the field's understanding of ripples and memory in the human brain.

Major issues:
1. A few particularly finicky analyses are applied that require significantly more validation to support the author’s conclusions:
a. Analysis of cortical reinstatement:
i. The basic method requires further validation and justification.
1. Grouping every cortical electrode suggests that the authors believe that every region of cortex sampled across their population is involved in reinstating the contents of a simple word-picture association in the same way, whereas much literature would suggest a slew of region-specific differences (e.g. PMID 39970226). The authors should justify this approach - perhaps through demonstrating that different cortical ROIs behave similarly. This is particularly important given the variance in cortical coverage across participants.

The reviewer is absolutely right that cortical reinstatement and representational geometry can vary across regions, and we did not intend to imply that all cortical sites behave identically. Our choice to aggregate all extra-hippocampal electrodes was motivated by two practical constraints: (i) highly heterogeneous electrode coverage across participants and (ii) the limited number of electrodes per ROI in any single participant.

To address the reviewer’s concern, we mapped all electrodes to MNI coordinates and compared our coordinate-defined ROIs to those in the study cited by the reviewer (PMID 39970226). Our posterior ROI ($x < -25 | x > 25$, $y < -30$, $z < 20$) captures lateral occipital and posterior occipitotemporal cortex, corresponding closely to BA17-19 and BA37 (Occ + pTC). Here we kept 9 participants. Similarly, our anterior ROI ($x < -25 | x > 25$, $y > -7$, $z < -20$) corresponds to ventral anterior temporal cortex (BA20; aTC). Here we kept 10 participants with more than 1 contact in the anterior ROI. Thus, our coordinate-based definitions map well onto the ROIs used in the cited study. We ensured no difference on a group level in channel count ($t(11) = -.12$, $p = .91$). We had in total 107 anterior channels ($M = 8.92$, $SE = 2.38$) and 112 posterior channels ($M = 9.33$, $SE = 1.9$).

We then repeated the key analyses separately within these ROIs. Although statistical power was limited due to sparse per-ROI coverage and lower participant count, the qualitative pattern of results was consistent across regions (see Figure below). Importantly, none of the ROIs showed an opposing pattern.

These results suggest that ripple-related reinstatement and representational expansion are spatially distributed across temporal and occipitotemporal cortex, justifying our main-group analyses while also addressing the reviewer’s concern about regional variability.

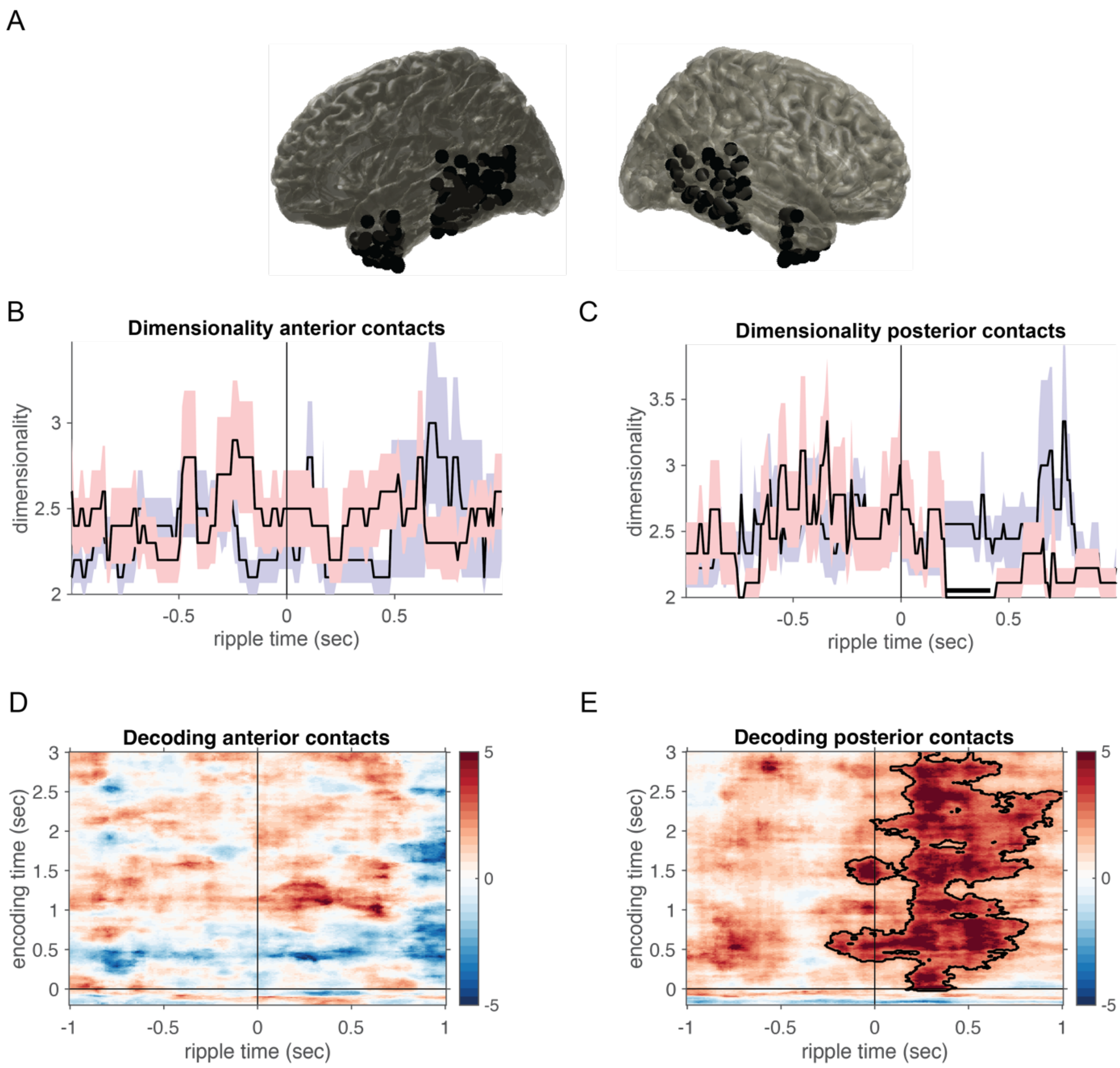

Dimensionality and decoding analyses using anterior and posterior cortical contacts. (A) Included anterior and posterior contacts. **(B)** No significant difference between AM+ and AM- for anterior contacts, but with the same increase around 600-800ms as in Fig 3B. **(C)** Significant difference between AM+ and AM- in a similar time window as in Fig. 3B. **(D)** No significant decoding difference between AM+ and AM- for anterior contacts, but with largest difference in the same post-ripple time window as in Fig. 3A. **(E)** Significant difference post ripple between AM+ and AM- for posterior contacts.

2. The relationship of cortical activity to ripples is
ii. There is insufficient detail to judge the reinstatement analysis.
1. I am not sure I understand the rationale for not performing cross-validation with a test in the encoding data. How do I know that the trained decoder is stable? A similar classifier-generalization analysis wa s used in a similar task by the authors' collaborators (from whom they received the data for this project), and cross-validation was performed in this case (PMID: 34880133).

We thank the reviewer for this comment and agree that it is important to validate the classifier at encoding before we assess generalisation. We used a 5-fold cross-validation,

training and testing at encoding and found significant decoding for both AM+ and AM- trials, compared to a 50% baseline.

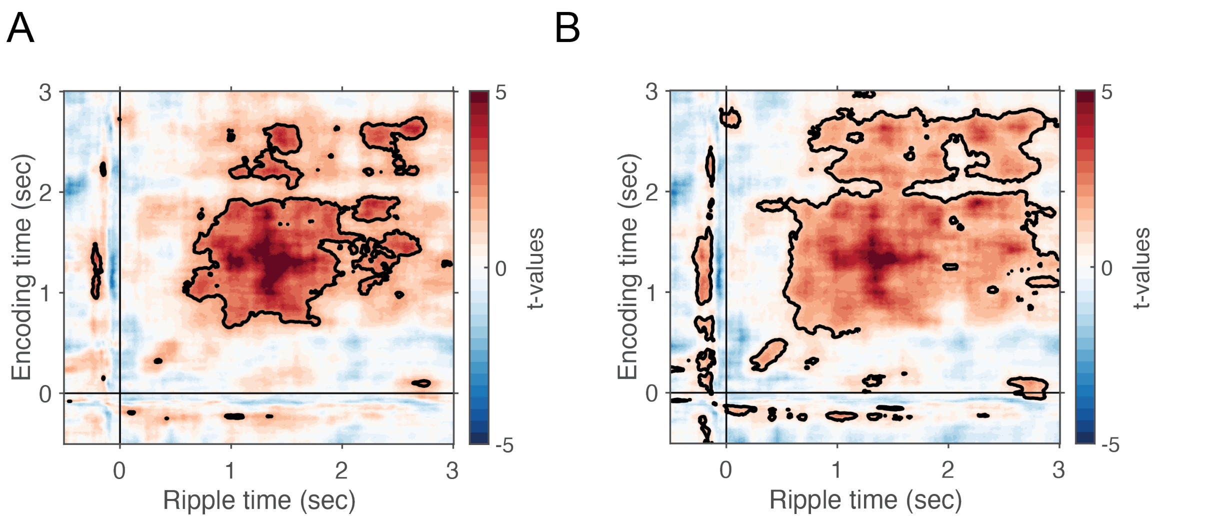

5-fold cross-validated decoding analysis. **A.** When using a 5-fold cross-validation training and testing at encoding, we found significant decoding, compared to a 50% baseline for AM+ trials. **B.** Similar results was found for AM- trials.

2. Treating "don't know" responses as equivalent to incorrect responses assumes the neural signature of failed memory is the same for guesses and explicit failures. That assumption affects both decoding comparisons (AM+ vs AM-) and dimensionality calculations that later depend on trial labels. They did this for balancing, which I get, but they need to show the main effects survive excluding such trials.

We thank the reviewer for raising this important concern regarding the heterogeneity of the AM- condition, which contains both incorrect responses and "don't know" responses. To address the reviewer's concern, we conducted additional analyses treating incorrect and don't know trials as separate categories.

Dimensionality analyses.
Both AM+ vs. incorrect and AM+ vs. don't know comparisons showed significant increases in post-ripple cortical dimensionality (same window as Fig. 3B). That is, successful retrieval reliably engaged higher-dimensional cortical states regardless of whether the comparison class was uncertainty-driven or error-driven.

Decoding analyses.
When comparing AM+ vs. don't know, we observed a significant decoding difference in the same post-ripple time window reported in Fig. 3A. When comparing AM+ vs. incorrect, we found a trend-level cluster (p = .09) in the very same time window. The temporal profile of both contrasts closely matched the original AM+ vs. AM- effect, indicating that the reinstatement differences are not specific to the "don't know" category.

Together, these results demonstrate that our main findings of enhanced reinstatement and increased dimensionality hold when don't know and incorrect trials are analysed separately.

Guided by the reviewer's suggestion we have now included these analyses and the related figure in the revised manuscript (P. 6, l. 19-20; Supplementary Fig. 4; and below).

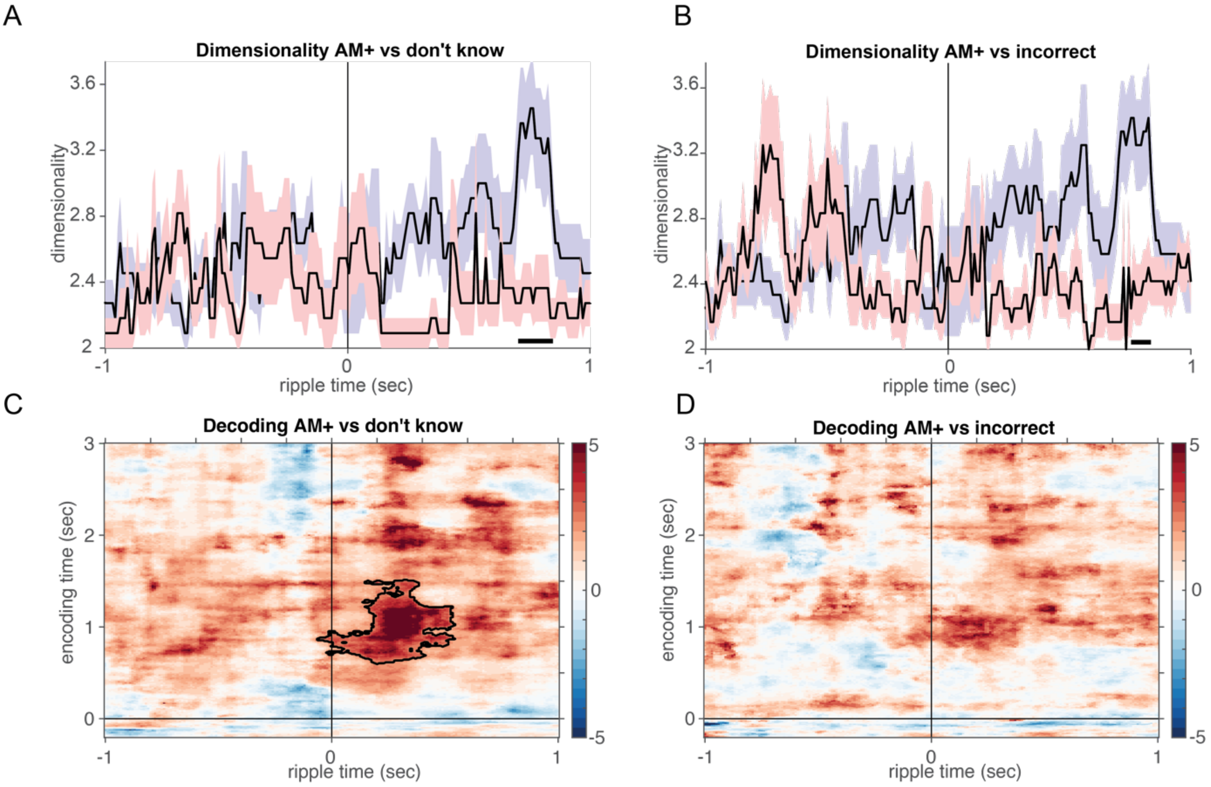

Dimensionality and reinstatement analyses dividing AM- into incorrect and don't know responses. **(A)** AM+ trials showed significant higher dimensionality as compared to don't know responses in a similar time window as main analysis in Fig. 3B. **(B)** AM+ trials showed significant higher dimensionality as compared to incorrect responses in a similar time window as main analysis in Fig. 3B. **(C)** AM+ trials showed significant higher decoding accuracy as compared to don't know responses in a similar time window as main analysis in Fig. 3A. **(D)** AM+ trials showed numerically higher decoding accuracy as compared to incorrect responses in a similar time window as main analysis in Fig. 3A.

b. PCA

i. The authors use a thresholded automatic artifact rejection process to get rid of putative interictal activity. Combined with shuffle-based controls, this might typically be sufficient for most analyses. But I am not convinced it's sufficient for PCA, where there might be a lot of sub-threshold interictal activity which can still explain a lot of variance in individual patients.

We thank the reviewer for raising this important point regarding the potential contribution of epileptiform activity to our ripple-locked findings. We implemented a comprehensive control pipeline to detect, quantify, and analyse epileptic spikes.

Our artefact rejection pipeline is intentionally conservative and designed to remove a broad class of transient noise events, including, but not limited to, epileptiform spikes. The procedure detects large-amplitude deviations, sharp voltage gradients, and broadband high-frequency transients, all of which are characteristic of pathological spikes but also of non-physiological artefacts such as movement or electrical noise. Thus, while the method effectively removes most spike-like events, it should not be interpreted as a dedicated

epileptic-spike detector. In response to the reviewer's request, we therefore implemented an additional spike-specific analysis

Since we used this method to detect artefacts, we include results where we quantified the fraction of all data points in a trial that fell within an automatically detected artefact segment, summed across channels and normalised by the total number of channel × time samples in that trial. A value of 1 would suggest that every channel-sample in that trial was marked as an artefact. Across participants, only .09% of channel×time samples within ripple-locked retrieval trials overlapped with artefact segments (SE = .03%), indicating low levels of contamination.

To identify interictal epileptiform discharges (IEDs), we applied a previously used outlier-based spike detection algorithm (Honey et al., 2012; Michelmann et al., 2021), in which transient high-amplitude deflections are detected as samples exceeding a robust amplitude threshold relative to each channel's distribution. Specifically, for each hippocampal channel, we computed the channel median and interquartile range (IQR) over the analysed recording segment and flagged candidate spike samples whose absolute deviation from the median exceeded 2 × IQR, considering both positive and negative deflections. To remove the detected events from the continuous signal, candidate spike samples were temporally dilated by ±10 ms and the affected samples were replaced using piecewise cubic interpolation from the surrounding non-spike samples. For alignment analyses, we collapsed each suprathreshold segment to a single peak sample by selecting, within each contiguous suprathreshold run, the sample with maximal absolute deviation from the channel median. To avoid counting high-frequency burst activity as multiple spikes, we imposed a 100 ms within-channel refractory period, retaining only the first detected peak within each refractory window. Spike-triggered averages were computed by extracting ±100 ms epochs around each spike peak. Epochs were high-pass filtered at 5 Hz and baseline-corrected by subtracting the mean voltage in a pre-spike window (−100 to −20 ms) prior to averaging across spikes and channels.

We computed spike-triggered averages from hippocampal channels and found no difference between number of spikes for AM+ and AM- trials ($t(11) = -1.15$, $p = .27$. This indicates that epileptiform activity is not selectively elevated on remembered vs. forgotten trials.

We repeated the full reinstatement analysis (Fig. 3A), aligning data to spike peaks instead of ripple peaks. No significant cluster emerged for spike-aligned reinstatement, contrasting AM+ and AM- trials. This stands in sharp contrast to the robust reinstatement observed when aligning to ripples. Finally, we repeated the PCA-based dimensionality analyses (Fig. 3B) using spike alignment. Again, we found no significant increase for AM+ trials compared to AM- trials.

Across all analyses requested by the reviewer, number of spikes for AM+ and AM-, reinstatement and PCA epileptic spikes failed to produce any of the neural signatures we observe around ripples. This demonstrates that the ripple-locked effects in reinstatement and dimensionality cannot be attributed to epileptiform artefacts.

We thank the reviewer for pointing out this important point and have now included these analyses in the revised manuscript P.33-34 and Supplementary Fig. 12 and 13).

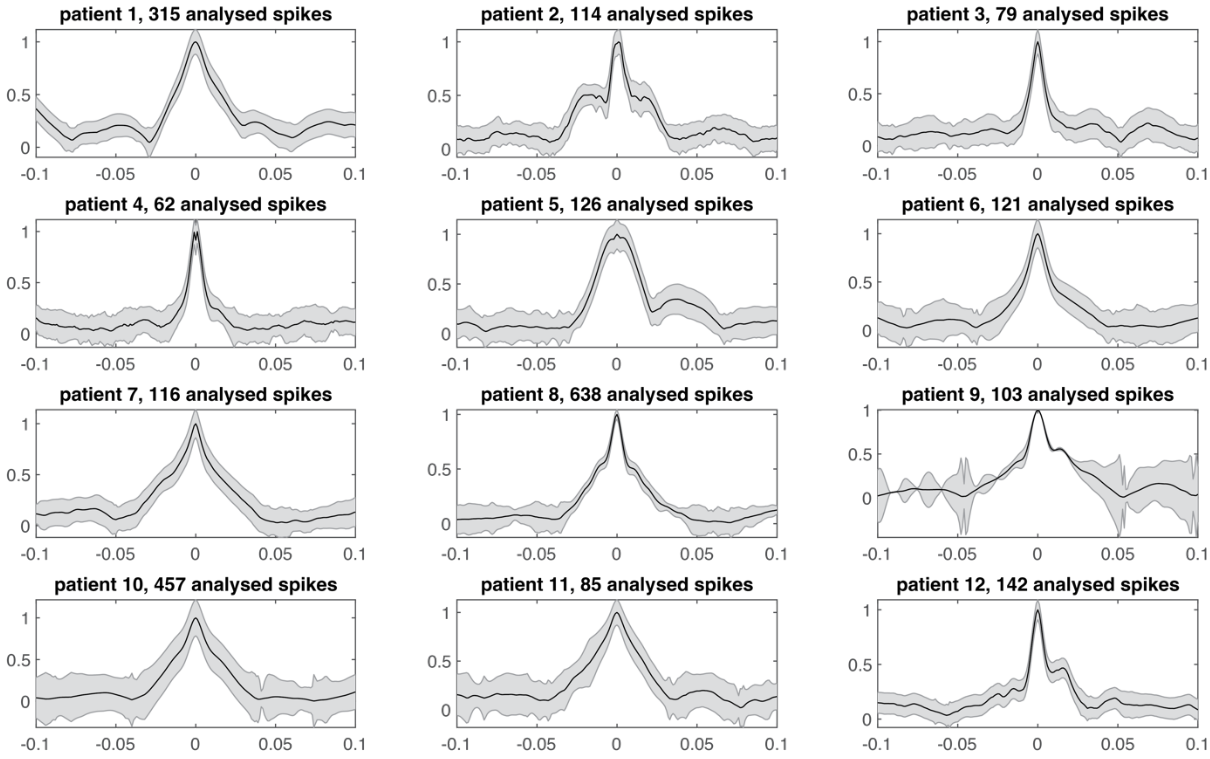

Spike-triggered averages. *For each participant, we extracted and averaged spikes, with the number of spikes above each spike-triggered average.*

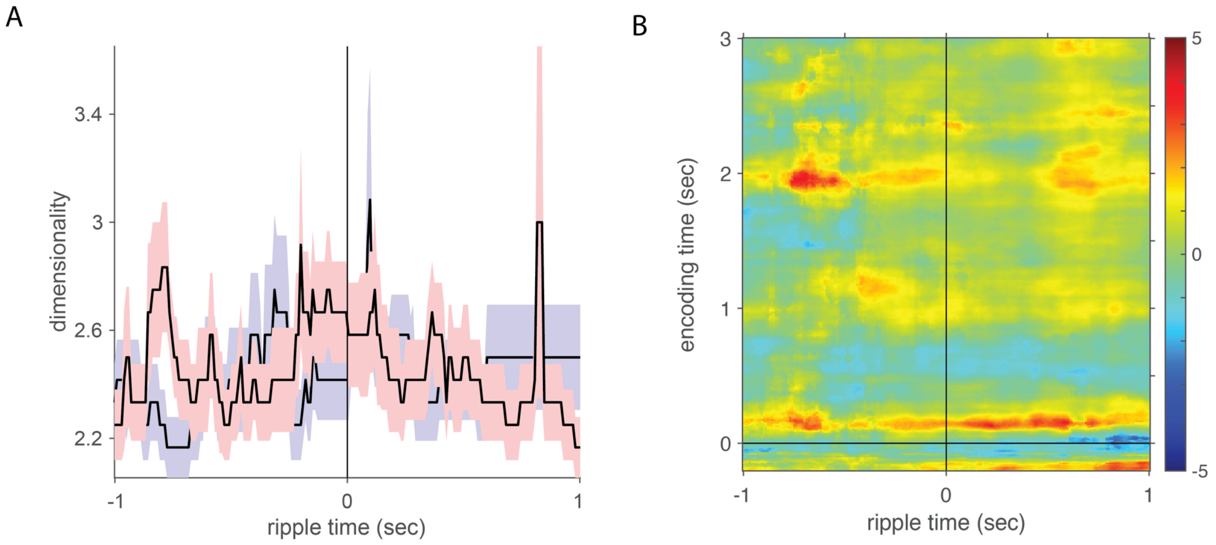

Dimensionality and decoding analyses based aligned on spikes. (***A***) Dimensionality analysis as in Fig. 3B showed no difference between AM+ (blue) and AM- trials (red). (***B***) Decoding analysis as in Fig. 3A showed no difference between AM+ and AM- trials.

ii. On a related note, the clean correlation between reaction time and dimensionality is actually a little concerning to me. Memory retrieval performance is often impaired due to interictal activity in contralateral to the seizure focus (which most of the sample hippocampi are; PMID 23685931). Can the authors verify that slow reaction times are not, in part,

explained by prevalence of subthreshold IEDs, and that these, in turn, do not lead to poor ripple identification or systematically lower dimensionality estimates?

We thank the reviewer for this thoughtful concern. We are aware of evidence that interictal epileptiform discharges (IEDs), including those arising contralateral to the seizure focus, can impair memory performance (PMID: 23685931), and we agree that any correlation between reaction time and dimensionality must therefore be examined carefully.

To address the reviewer's concern that subthreshold interictal discharges might jointly influence retrieval speed and our ripple-locked dimensionality estimates, we computed, for each retrieval trial, the proportion of samples overlapping artefact-marked segments in the continuous data (including padding). This yielded trial-wise indices of residual artefact load (that were interpolated in all our analyses) that were entered into correlation and regression analyses alongside reaction time and dimensionality change. Artefact load did not correlate with reaction time (Spearman ρ = -.28, p = .38) nor with dimensionality change (ρ = .27, p = .39). Crucially, the robust relationship between reaction time and dimensionality (ρ = -.67, p = .017) remained essentially unchanged after controlling for artefact load in a multiple regression (standardised β_RT = -.66; β_artifact = -.01). These analyses indicate that the reaction time-dimensionality association cannot be attributed to residual interictal activity or trial-level data quality. Consistent with this, ripple detection explicitly excludes all events overlapping artefact-marked segments (plus padding), ensuring that ripple-centred PCA windows are free of high-amplitude or pathological transients.

iii. Similarly, is dimensionality related to n electrodes? With n electrodes likely correlated with theorized epileptic spread, and thus potentially with RTs?

To address the possibility that variation in the number of cortical electrodes contributing to the PCA might influence our dimensionality estimates and might therefore confound the observed relation between reaction time (RT) and ripple-locked dimensionality, we extracted, for each participant, the number of electrodes included in the PCA, their mean RT, and their ripple-locked dimensionality change. The number of electrodes was not related to dimensionality (Spearman ρ = -.018, p = .96), indicating that sampling density did not bias the dimensionality estimates. Electrode count showed a trend-level association with RT (ρ = -.53, p = .076), suggesting that participants with more extensive coverage tended, if anything, to respond slightly more quickly. Crucially, when RT and electrode count were entered simultaneously into a multiple regression predicting dimensionality, the effect of RT remained strong (standardised β = -.95), whereas electrode count exerted a much smaller influence (β = -.56). Thus, although electrode count explains some variance in the model, the RT-dimensionality relationship survives when controlling for this factor, demonstrating that the key effect cannot be attributed to differences in the number of electrodes or to broader differences in sampling related to epileptic spread.

iv. How does the authors' unique referencing choice influence the PCA/dPCA results? A common average may lead to less local signal than, say, a bipolar reference. And I've never seen a trimmed common-average before; the implications are unclear for principal components analyses given that it removes electrodes which may contribute to large amounts of variance in a common average.

We thank the reviewer for suggesting a control analysis using an alternative referencing scheme. We believe that bipolar referencing is not an appropriate baseline against which to evaluate variance-based multivariate measures such as PCA or dPCA, mainly because bipolar referencing fundamentally alters the variance structure of the data in a way that biases dimensionality estimates.

Bipolar referencing acts as a spatial differencing operation, effectively functioning as a spatial high-pass filter. This reweighting attenuates spatially broad, low-frequency components and emphasises highly local fluctuations between neighbouring contacts. As a consequence, signals that are shared across larger cortical territories (precisely the components that contribute most strongly to population-level variance structure) are systematically suppressed. In addition, bipolar referencing reduces the rank of the data by construction, as each shaft with n contacts yields only n–1 bipolar channels, which further biases eigenspectrum-based dimensionality estimates downward. For these reasons, bipolar referencing is known to be suboptimal for analyses that aim to characterise distributed population geometry rather than highly focal effects.

Importantly, referencing constitutes a linear reweighting of the original signals and should not, in itself, introduce differences between experimental conditions or time windows. Rather, it selectively emphasises certain spatial components of the data while suppressing others. Thus, any attenuation of ripple-related dimensionality effects under bipolar referencing reflects the altered statistical properties of the signal, not the absence of the underlying neural phenomenon.

Nonetheless, to address the reviewer's concern directly, we repeated the main analyses under bipolar referencing. Ripple density was again significantly higher for AM+ compared to AM− trials ($M = .25$, $SE = .02$; $t(11) = 4.12$, $p = .002$), and ripple-aligned time–frequency representations were highly similar across referencing schemes, with consistent peak ripple frequencies at 90Hz (89Hz with trimmed average). The decoding results were also highly similar to the main Figure 3A. By contrast, PCA-based dimensionality estimates were reduced under bipolar referencing, consistent with the known suppression of globally shared variance.

We interpret this pattern as indicating that ripple-related dimensionality expansion reflects distributed, large-scale changes in cortical population activity, whereas more local pattern-based effects, such as decoding remain detectable under both reference schemes. Given our specific interest in global representational geometry across widespread cortical sites, we therefore retain our referencing as the primary analysis approach, while reporting the bipolar-referenced control analyses for completeness in the revised manuscript.

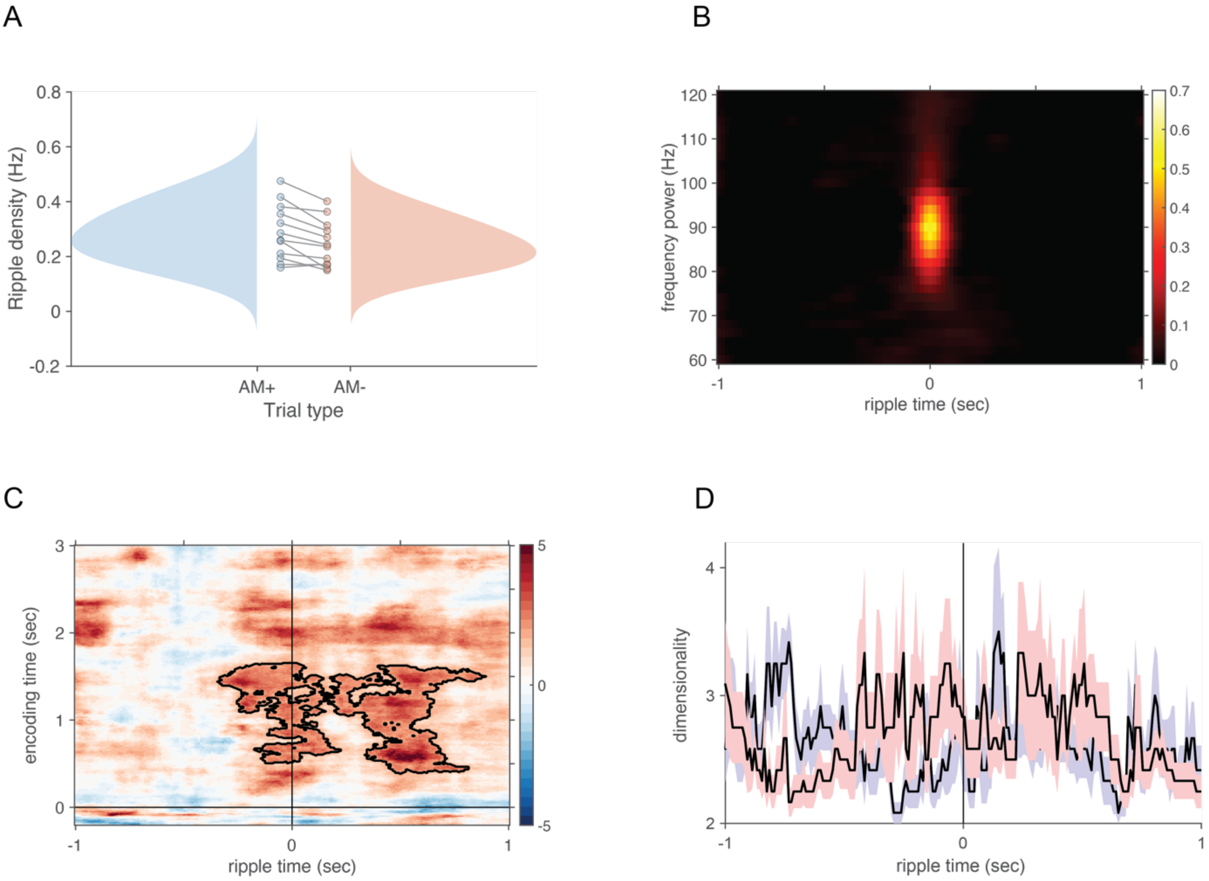

Analyses using bipolar reference. (A) Ripple density was significantly higher for AM+ vs. AM- trials. (B) Spectral grand average was almost identical to main analysis Fig. 2A. (C) Reinstatement analysis showed similar cluster as in main Figure 3A. (D) Time-resolved dimensionality analysis did not show the same significant cluster around 400-800ms post ripple, which is to be expected under bipolar reference scheme.

v. The use of time-resolved PCA is warrants further validation. With time windows that short (<=200 ms), are dimensionality estimates robust to noise?

We agree that short time windows could, in principle, make dimensionality estimates vulnerable to noise, and we therefore performed several control analyses to assess the robustness of our results. First, we explicitly varied the temporal window length used for PCA: in addition to our main 60 ms sliding window (90% overlap), we repeated the analysis with 100 ms and 200 ms windows. Across these parameter settings, the temporal profile of dimensionality and the AM+ / AM- difference were highly consistent (Supplemental Fig. 6A-B in revised manuscript), indicating that the effect is not driven by a particular, arbitrarily chosen window size.

Second, the dimensionality estimates themselves were stable across alternative methods and metrics. Our primary measure used the second-derivative ("elbow") criterion on the eigenvalue spectrum; we obtained very similar results when quantifying dimensionality as the distance to a fitted power-law, when using effective dimensionality (ED) and based on a suggestion from Reviewer #3, we also used Marchenko-Pastur threshold as alternative methods (Methods, and in revised manuscript included as Supplemental Figure 6F). Moreover, the retained components accounted for substantial variance in the data within the

significant time window (AM+: M = 53.85%, SD = 10.06%; AM-: M = 46.87%, SD = 13.17%), arguing against the notion that we are estimating dimensionality from purely noisy fluctuations.

Third, we directly validated that the reduced component space preserved behaviourally relevant information by reconstructing the data back into the original feature space and re-running the time-generalised decoding analysis. Decoding accuracy based on the reconstructed data closely tracked decoding from the full data (Spearman's ρ = .87, p < .001; Supplemental Fig. 6C), demonstrating that the components identified within these short windows capture the same class-selective structure as the original signal, rather than noise.

Finally, we confirmed that ripple-locked dimensionality expansion was specific and not attributable to generic noise or windowing artefacts: (i) AM+ trials showed higher dimensionality than a pre-stimulus baseline (t(11) = 3.80, p = .003), (ii) linear mixed-effects models revealed a significant interaction between condition (AM+/AM-) and time (pre/post ripple), and (iii) ripple-aligned surrogate controls (RetrCorrect, RandCorrect and CircCorrect) showed significantly lower dimensionality than the empirical data (Supplementary Fig. 8).

Taken together, these converging analyses indicate that our time-resolved PCA estimates are robust across window lengths and analysis choices, and that the observed ripple-locked increase in dimensionality reflects structured, behaviourally meaningful signal rather than noise.

c. PAC
i. Within-electrode PAC is already hard to identify; true long-distance PAC is even rarer and frankly I question the robustness of this finding. As the authors state, Figure 4 would represent the "the first evidence of hippocampal theta-cortical gamma phase-amplitude" and I think this warrants quite a bit more investigation to validated. So little has been done to make me trust this analysis that I find it a bit difficult to assess overall (it's not even clear how PAC was computed! Modulation index?)

We thank the reviewer for raising this important concern and for prompting us to clarify and further validate the PAC analysis.

Given that long-distance PAC is challenging to establish and can be confounded by signal properties, our primary goal was to use a conservative, well-validated coupling metric and to explicitly rule out known sources of spurious PAC. We therefore used a standard and widely adopted PAC measure: the mean vector length originally introduced by Bragin et al. (1995). This metric has been extensively used in both animal and human hippocampal PAC studies Canolty et al., 2006; Jensen & Colgin, 2007; Lakatos et al., 2005; ; Griffiths et al., 2021). Specifically, PAC was calculated as the magnitude of the complex average A(t)eiϕ(t), where A(t) is cortical gamma amplitude and ϕ(t) is hippocampal theta phase, yielding the classical PAC measure.

To ensure robustness, we used narrow-band frequency windows centred on subject-specific theta and gamma peaks (defined after 1/f removal using FOOOF). Surrogate distributions were created by shuffling trial identities of the gamma amplitude 250 times per participant

and frequency, destroying phase-amplitude relationships while preserving temporal autocorrelation. PAC values were averaged across hippocampus-cortex channel pairs and significance assessed using cluster-based permutation testing. We have now increased the baseline to include 500 permutations instead of before 250, with the result being identical to before.

Because waveform asymmetries can spuriously inflate PAC estimates, we explicitly quantified multiple gamma waveform-shape metrics, including peak-trough sharpness, rise-decay asymmetry, skewness, and kurtosis. Gamma waveforms were highly symmetric (peak–trough ratio: mean = 1.000, SD = .0045; rise–decay ratio: mean = 1.000, SD = .0021), exhibited negligible skewness, and showed kurtosis values consistent with narrowband filtering (mean = 5.98, SD = 14.04). Crucially, none of these metrics correlated with preferred gamma frequency (all $p > 0.17$), indicating that neither regional variation in gamma frequency nor waveform asymmetry could account for the observed PAC effects.

We thank the reviewer for highlighting this important point and hope that this clarification addresses the reviewer's concern. Guided by the reviewer, we have now included these analyses in the revised manuscript (P. 12, l. 19-25; P. 31, l. 21 – P. 32, l. 24; and Supplementary Fig. 10 and 11).

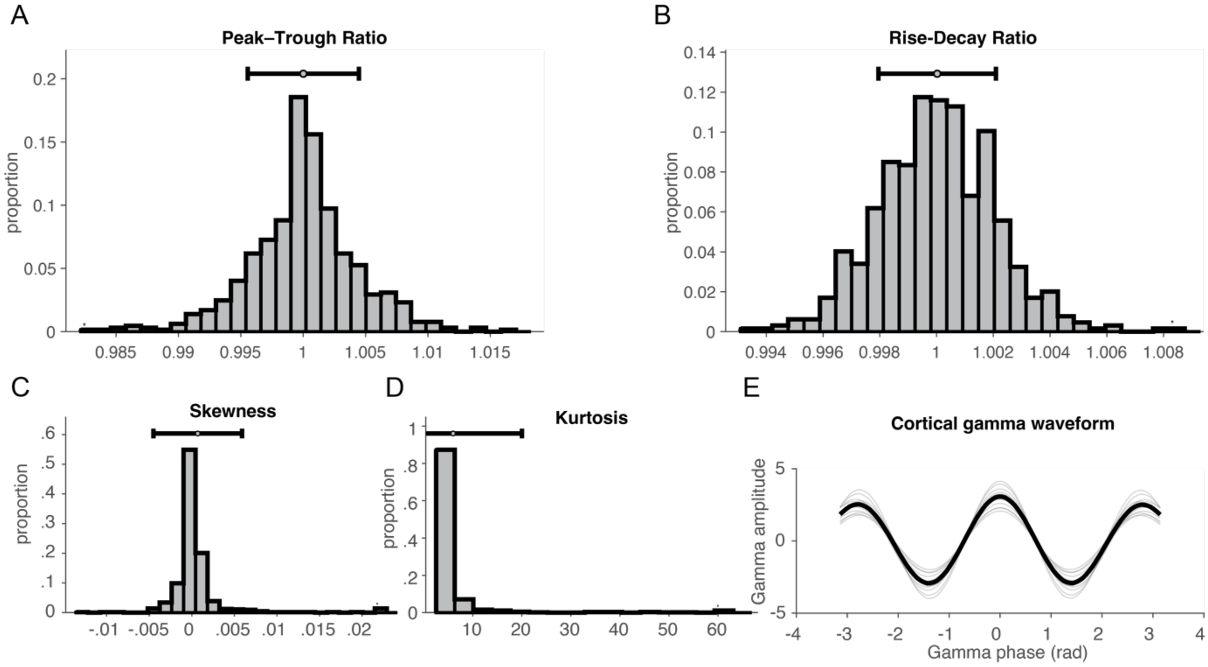

Gamma waveform-shape metrics. **(A)** Peak-trough sharpness ratio: ratio of mean peak sharpness to mean trough sharpness, reflecting symmetry of extrema. Values tightly clustered around 1 indicate near-perfect symmetry. **(B)** Rise-decay ratio: ratio of mean rise time (trough to peak) to mean decay time (peak to trough). Ratios near 1 indicate minimally asymmetric cycles. **(C)** Skewness of the gamma-band waveform, indexing asymmetry of the signal's amplitude distribution (values centred at zero indicate symmetric waveforms). **(D)** Kurtosis, reflecting the heaviness of the tails of the amplitude distribution. The distribution is consistent with narrowband-filtered gamma activity, and no systematic deviations were observed. **(E)** The average cortical gamma waveform.

1. I find the averaging across channel pairs across the entire cortical sampling here very

obfuscating. For example, given that the authors align PAC to local peaks, what would it even mean for 5 Hz to coupled to 90 Hz in temporal cortex vs. 55 Hz in parietal cortex? Do the authors really think theta oscillations in the hippocampus propagate in the same way, and at the same time, across all of cortex? Even at the level of the hippocampus, theta power and frequency differs across the longitudinal axis- does it make sense to average across anterior and posterior hippocampal electrodes?

We thank the reviewer for pointing out the need for clarification. We estimated the peak per channel, but averaged over all the channels within each region (hippocampus and cortex separately) to obtain an average of that region's dominant theta and gamma peak. PAC was then computed using these region-level peak frequencies, not channel-specific peaks, and all subsequent PAC analyses were aligned to these regional peak values (theta: ±2 Hz; gamma: ±10 Hz).

This means the analysis did not assume that individual cortical sites contain identical gamma peaks or that hippocampal theta propagates uniformly across cortex. Instead, the procedure identifies the dominant oscillatory mode in each region at the population level and quantifies how strongly that mode participates in cross-frequency coupling around ripple events. This approach has two advantages:

1. *It avoids spurious heterogeneity arising from noisy peak estimates at individual channels.*

2. *It ensures comparability across participants, many of whom have sparse and idiosyncratic electrode coverage that precludes fine-grained anatomical sub-partitioning (e.g., anterior vs. posterior hippocampus, or temporal vs. parietal cortex).*

Nonetheless, to address the reviewer's concern, we directly quantified the dispersion of per-channel peak frequencies around the region-level (hippocampal or cortical) peaks used in the PAC analysis.

Hippocampal theta peaks showed very low variability, with a mean standard deviation of 1.88 Hz, a median absolute deviation (MAD) of .17 Hz, and 74.8% of hippocampal channels falling within ±2 Hz of the participant-level peak. This confirms that hippocampal theta oscillations are strongly clustered around a single narrowband frequency within each participant, justifying the use of a region-level theta peak for alignment.

Cortical gamma peaks exhibited broader dispersion, as expected for gamma-band activity across distributed cortical sites (mean SD = 15.9 Hz, mean MAD = 0.42 Hz). Importantly, the low MAD indicates that most channels' gamma peaks lie close to the participant-level peak, and the larger SD is driven by a small number of high-frequency outliers. Even so, 31.1% of channels fell within ±10 Hz of the regional gamma peak, consistent with prior work showing substantial regional variability in cortical gamma (e.g., Buzsáki & Wang, 2012).

Together, these analyses demonstrate that the region-level peak frequencies used in our PAC analysis accurately reflect the central oscillatory modes of hippocampal theta and to some

extent cortical gamma, and that the procedure does not mask large channel-wise differences.

Finally, we note that the goal of the PAC analysis was not to map precise propagation patterns across the cortical surface, but to test whether hippocampal theta phase modulates cortical gamma amplitude at ripple timing, and whether this coupling relates to dimensionality expansion. The regional-peak approach is well-suited for this purpose and avoids the interpretational problems highlighted by the reviewer.

Guided by the reviewer's suggestions, we have now added these analyses to the Result section P.12, l.28 – P. 13, l. 3 and Method section P.31, l.5-19.

2. Does the trial-level shuffling (only 250 permutations) address concerns about gamma waveform shape contributions to PAC? Does gamma waveform shape differ in different regions or across different frequencies of gamma oscillation?

We agree that non-sinusoidal waveform shape could spuriously inflate within-channel PAC estimates. Our analytical approach addresses this in two ways. First, our PAC measure is computed across structures (hippocampal theta phase × cortical gamma amplitude), rather than within the same signal, which already reduces sensitivity to purely local waveform confounds. Second, our trial-shuffled surrogate preserves the single-trial gamma waveforms (including any non-sinusoidal morphology and their alignment to ripple onset) and only disrupts the cross-trial relationship between hippocampal phase and cortical amplitude. Any PAC driven solely by gamma waveform shape or local autocorrelation structure is therefore captured in the surrogate distribution. The fact that empirical PAC significantly exceeds this surrogate indicates a consistent hippocampal-cortical coupling beyond what waveform shape alone can explain.

To directly address the reviewer's concern, we (i) increased the number of trial-shuffled surrogates from 250 to 500, obtaining an indistinguishable pattern of significant PAC (see Figure below); (ii) quantified gamma waveform asymmetry (peak-trough sharpness and rise-decay asymmetry) across cortical regions and gamma sub-bands, finding no systematic differences that parallel the PAC effects. Together, these controls make it unlikely that our hippocampal theta-cortical gamma PAC findings are driven by regional differences in gamma waveform shape.

To directly assess (ii), we quantified several gamma waveform-shape metrics for each cortical channel at the frequency used for PAC analysis, including peak-trough sharpness, rise-decay asymmetry, skewness, and kurtosis. Gamma waveforms were highly symmetric (peak-trough ratio mean = 1.000, SD = .0045; rise-decay ratio mean = 1.000, SD = .0021), exhibited negligible skewness, and showed kurtosis distributions consistent with narrowband filtering (mean = 5.98, SD = 14.04). Crucially, none of these metrics correlated with the preferred gamma frequency (all $p > .17$), indicating that neither regional variation in gamma frequency nor waveform asymmetry could account for the PAC effects. We also correlated the waveform metrics with our PAC effect and found no relationship (all $p > .2$). Moreover, our surrogate analysis preserves waveform shape while disrupting cross-trial phase-amplitude alignment, further ensuring that the observed PAC reflects genuine hippocampal-

cortical coupling rather than waveform artefacts. Below, we include the figures showing the effect with 500 permutations for the baseline and for the gamma wave-shape metrics that was also added to the Reviewer's comment c.

These results have now been added to the Result section P.12, l. 19-25 and Method section P.31, l. 21 - P. 32, l. 24.

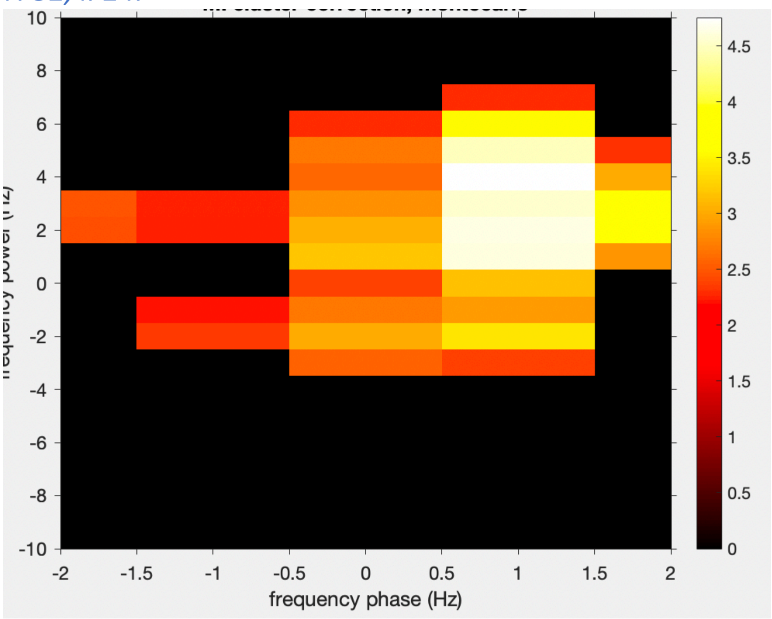

TG-PAC analysis yields identical results when increasing the shuffling from 250 to 500 permutations.

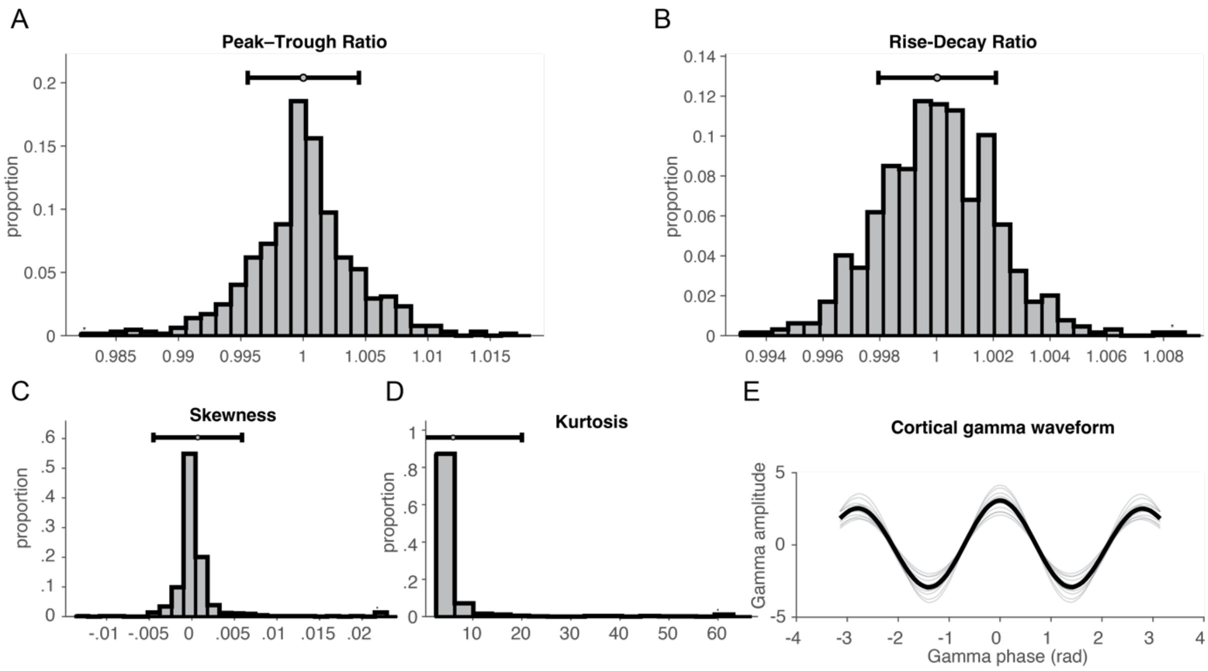

Gamma waveform-shape metrics. **(A)** Peak-trough sharpness ratio: ratio of mean peak sharpness to mean trough sharpness, reflecting symmetry of extrema. Values tightly clustered around 1 indicate near-perfect symmetry. **(B)** Rise-decay ratio: ratio of mean rise time (trough to peak) to mean decay time (peak to trough). Ratios near 1 indicate minimally asymmetric cycles. **(C)** Skewness of the gamma-band waveform, indexing asymmetry of the signal's amplitude

distribution (values centred at zero indicate symmetric waveforms). **(D)** Kurtosis, reflecting the heaviness of the tails of the amplitude distribution. The distribution is consistent with narrowband-filtered gamma activity, and no systematic deviations were observed. **(E)** The average cortical gamma waveform.

3. What is the relationship of ripple activity and theta power in the hippocampus? Theta power and memory?

We thank the reviewer for this important point. We conducted additional analyses to assess the relationship between ripple activity and theta power and theta power and memory around ripple events.

Using one ripple per trial, and estimating theta power in 3-8Hz, although we observe a general increase of theta power around the ripple peak, we find no difference in theta power between correct and incorrect trials (see fig below). We then correlated theta power with ripple amplitude. Although we see the highest correlation at ripple peak, this correlation is very weak and does not survive any correction across time. We also correlated number of ripples with theta power, and we observed a negative correlation around the ripple peak, which also did not survive correction across time. In sum, we find no relationship between theta power and memory around ripple events and also no relationship between theta power and ripple characteristics.

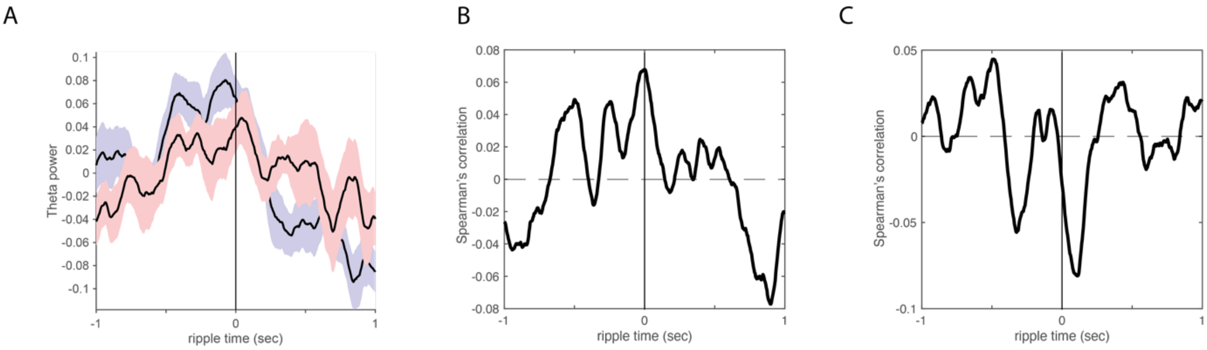

Relationship ripples and theta power. ***(A)*** *AM+ and AM- trials showed no difference in theta power around ripple events.* ***(B)*** *Correlation between ripple amplitude and theta.* ***(C)*** *Correlation between number of ripples and theta power.*

ii. The authors cite two papers by Vaz et al., but ignores the most pertinent findings from that group involving cortical ripples coupling to MTL ripples. I find it hard to square their findings of ripple-induced cortical PAC with cortical gamma with the prevalence of cortical ripples and consider this a major gap in understanding the implications of this paper in relation to existing literature.

We thank the reviewer for pointing this out and agree that the work by Vaz and colleagues on cortical ripples is highly relevant for interpreting our findings.

First, our high-frequency gamma band is defined in a data-driven way using FOOOF, and TG-PAC is computed on narrow-band peaks in the 40-140 Hz range and we measure the modulation of narrow-band high-frequency amplitude in cortex by hippocampal theta phase.

In that sense, our findings are fully consistent with the idea that hippocampal ripples coordinate widespread networks of fast cortical events, including cortical ripples as described by Vaz et al.

Second, there are also some important differences in focus and emphasis. Vaz and colleagues primarily characterised co-occurrence and coupling between ripples across hippocampus and cortex and showed that cortical ripples are widespread and strongly coupled to MTL ripples during memory processes. In contrast, here we use hippocampal ripples as temporal anchors and ask how they relate to two specific cortical phenomena: (i) the expansion of representational dimensionality and (ii) narrow-band high-frequency activity whose amplitude is locked to hippocampal theta phase. Our TG-PAC measure should therefore be seen as capturing a mechanism of communication (a theta-dependent gain modulation of fast cortical activity) rather than as a competing account to ripple-ripple coupling.

Third, we now clarify in the text that our cortical gamma band likely includes contributions from ripple-range activity. This is particularly important given the ubiquity of cortical ripples reported by Vaz et al. Rather than claiming that hippocampal theta modulates gamma and not cortical ripples, we interpret our findings as showing that hippocampal theta rhythm gates a class of high-frequency cortical events, potentially including ripples, that precede and predict ripple-locked increases in cortical dimensionality. We now explicitly acknowledge this in the Discussion, P. 16, l. 7-12 and reads as following:

Importantly, we also acknowledge that our cortical gamma-band likely includes contributions from high-frequency events in the ripple range; rather than assuming strict separation between gamma and cortical ripples, we interpret our findings as evidence that hippocampal theta rhythm gates a broad class of high-frequency cortical activity, including potential ripples, that precedes and predicts ripple-locked increases in cortical dimensionality.

Finally, we have added a brief clarification in the Methods to make our PAC computation more transparent (phase-amplitude vector length, with a trial-shuffled baseline), and we explicitly state that the purpose of this analysis is not to distinguish between gamma and ripple sub-bands per se, but to establish a temporal link between hippocampal ripples, hippocampal theta, fast cortical activity and representational expansion (P. 30, l. 36-37 - P. 31, l. 1-3):

For clarity, we note that our PAC computation reflects the phase-amplitude vector length (theta phase × gamma-band amplitude) relative to a trial-shuffled baseline, and the goal of this analysis is not to distinguish between gamma and ripple sub-bands per se, but to establish a temporal link between hippocampal ripples, hippocampal theta, fast cortical activity, and the subsequent expansion of cortical representational dimensionality.

We hope that this additional context makes clear that our findings are compatible with, and complementary to, the existing literature on cortical ripples rather than in conflict with it.

d. dPCA

i. I admittedly don't know too much about dPCA; can the authors provide citations for other works using dPCA with field potentials recorded from epileptic direct-brain recordings to further support their application here? If not, and given my qualms with the other methods

here, I would request further empirical validation of the approach against potential pitfalls.

We thank the reviewer for raising this important point. Although dPCA was originally developed for high-dimensional single-unit recordings, the method has since been applied to human intracranial field potentials, including in epilepsy patients. Most relevantly, Fetterhoff et al. (2024) applied dPCA to iEEG field potentials recorded from depth and subdural electrodes in neurosurgical patients performing a memory task. Their implementation follows the same principles as ours (treating features (channels × time) as population activity and decomposing variance according to task variables) demonstrating that dPCA is appropriate for analysing human field potentials and capable of revealing representational structure in macroscopic signals.

Our analysis follows the procedure described in Kobak et al. (2016) and Fetterfoff et al (2024) and implemented in the openly released toolbox, with one important extension, namely that we introduced an additional multiple-comparison correction for decoding accuracy, using cluster-based permutation tests. Neither the original dPCA toolbox nor Fetterhoff et al. incorporated such correction. Including this step was critical for ensuring that all decoding results reflect statistically robust, temporally extended effects rather than noise-driven fluctuations.

Finally, we note that dPCA is well suited for iEEG because it does not require spiking data; it only assumes that the multichannel signal reflects population-level activity structured by task variables. This assumption is met by field potentials, which integrate activity over local ensembles and have been widely used for state-space analyses (e.g., PCA, LDA, demixing-based approaches). Thus, dPCA provides a principled and validated way to examine how task variables are represented and separated in cortical population activity.

Together, these points provide empirical, methodological, and conceptual support for the use of dPCA on human intracranial field potentials in the present study.

Across analyses:
2. The surrogate-based control analyses utilized here are unconvincing:
a. The statistical comparison between empirical and shuffled data seems very lenient. If I understand it correctly, the authors are conducting a paired t-test between the empirical decoding and the mean of the shuffles within each patient. Typically, one would be interested in whether the empirical data is systematically greater than 95% of the shuffled data within each patient.

a. The first shuffle method used (RandCorrect) also seems too liberal (it is easy to destroy classifier performance!), and yet the empirical data is still only barely better than the most liberal shuffle (RandCorrect, p=0.02), even given the lenient hypothesis testing mentioned above.
b. The second shuffle method used (CircCorrect) is more well-motivated but doesn't quite work (p=0.06). I think this could be because there seem to be only ~ 38 AM+ retrieval trials per person (if I am understanding the number of trials included correctly) - how many unique circular shuffles could there even be?

c. I would be more convinced by a control framework that really tests the central question more precisely. The central question is (as I understand): is the time following ripple onset special? For example, does the (largest) ripple's onset time t consistently predict reinstatement of feature-specific encoding activity better than alternative times during retrieval? So, a control analysis might test the decoding aligned to every other time in the trial, and then z-score ripple decoding compared to this population. Or, one could image performing the exact same analyses relative to IEDs rather than ripples!

We thank the reviewer for these thoughtful comments. We agree that the key question is whether the period following ripple onset is privileged relative to other moments during retrieval, and we have therefore implemented the specific control framework the reviewer suggests. We then re-evaluated the remaining surrogate comparisons using a more stringent group-level approach.

To test directly whether ripple onset provides a uniquely effective alignment point, we compared ripple-aligned reinstatement against a distribution obtained by aligning the analysis to alternative time points during retrieval. Specifically, we computed reinstatement for a set of pseudo-events spanning the retrieval epoch and then expressed ripple-aligned reinstatement relative to this distribution.

Rather than collapsing each participant's surrogate distribution to a single summary statistic, we now use a group-level permutation test (Stelzer et al., 2013). On each iteration, one surrogate estimate is sampled per participant and averaged across participants, yielding a group-level null distribution that preserves both within-participant and between-participant variability. The empirical group-mean cluster value is then compared to this null distribution to obtain a two-sided p-value. This framework provides a distribution-aware test at the group level and avoids overly liberal or overly conservative within-participant thresholding.

Using this approach, empirical reinstatement was significantly greater than the group-level null ($z = 6.05$, $p < .01$). This provides an explicit and direct confirmation of the reviewer's central concern: reinstatement is reliably stronger following ripple onset than when the same analysis is aligned to other moments during retrieval.

We agree that RandCorrect is comparatively liberal, in that it disrupts ripple-to-reinstatement alignment while remaining anchored to each participant's ripple events. Importantly, even under the updated group-level permutation framework, empirical reinstatement remained significantly greater than the RandCorrect null distribution ($z = 2.19$, $p = .029$). This indicates that the effect cannot be explained by random temporal alignment of ripple times.

CircCorrect is intentionally conservative because it preserves temporal autocorrelation and tests ripple onset against alternative temporal alignments within the retrieval epoch. Contrary to the reviewer's estimate, the number of AM+ trials contributing to decoding was higher (mean ≈ 58; range 31–144; Table S1), providing sufficient trials to support circular shifting. Using a paired-samples comparison within the empirically defined cluster, we had originally cautioned that a statistical trend for stronger reinstatement was observed with

CircCorrect (t(11) = 2.05, p = .06); however, we realised that for our directed hypothesis of an increase, we had mistakenly applied a two-sided t-test, which is overly conservative; the interpretation of the the stricter CircCorrect comparison, should therefore be adjusted to t(11) = 2.05, p = .03 . Critically, the new RetrCorrect analysis described above directly addresses the reviewer's central question with a strong positive result.

Finally, we agree that aligning the analysis to non-ripple events such as IEDs would be an informative additional comparison and have added this in response to comment b. PCA.

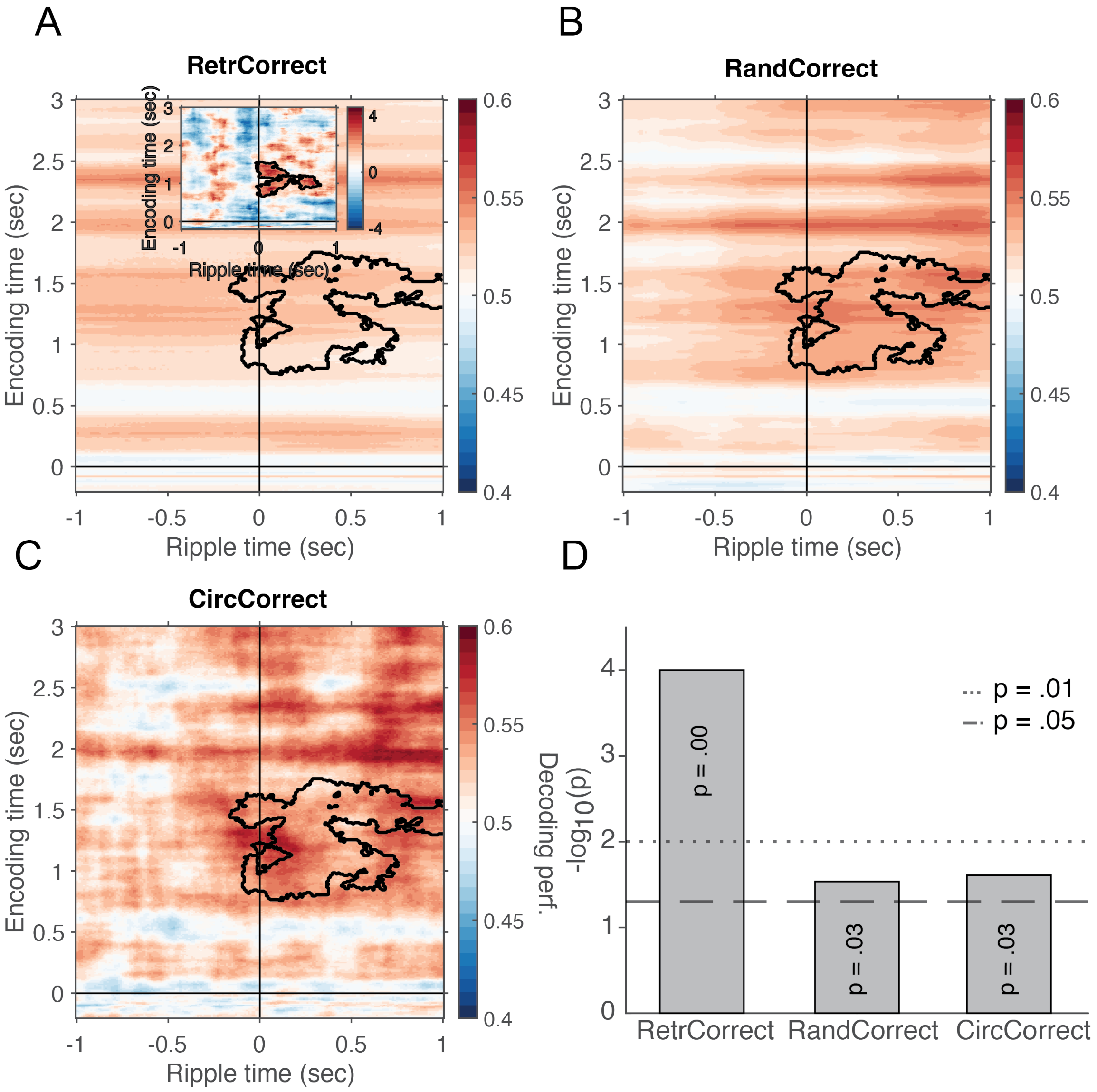

***Control analyses confirming ripple-triggered reinstatement specificity*. (A)** From 1-5 seconds after cue onset, we picked 60 time points and treated them as ripple events and performed the same analysis as in 3A. The time generalisation matrix shows the decoding accuracy for these events with the significant cluster identified in Figure 3A highlighted in black. Inset shows a cluster-based permutation between the permuted baseline and empirical data, with a significant cluster in the same time window as in Figure 3A. **(B)** Decoding accuracy when AM+ trials were shuffled 1000 times. **(C)** A similar analysis was performed using a stricter control in which ripple times were swapped with those of adjacent trials (i.e., neighbouring trial shuffling). This revealed significantly higher decoding in the

empirical AM+ condition compared to the adjacent-trial control. **(D)** P-values when comparing empirical data with the different controls.

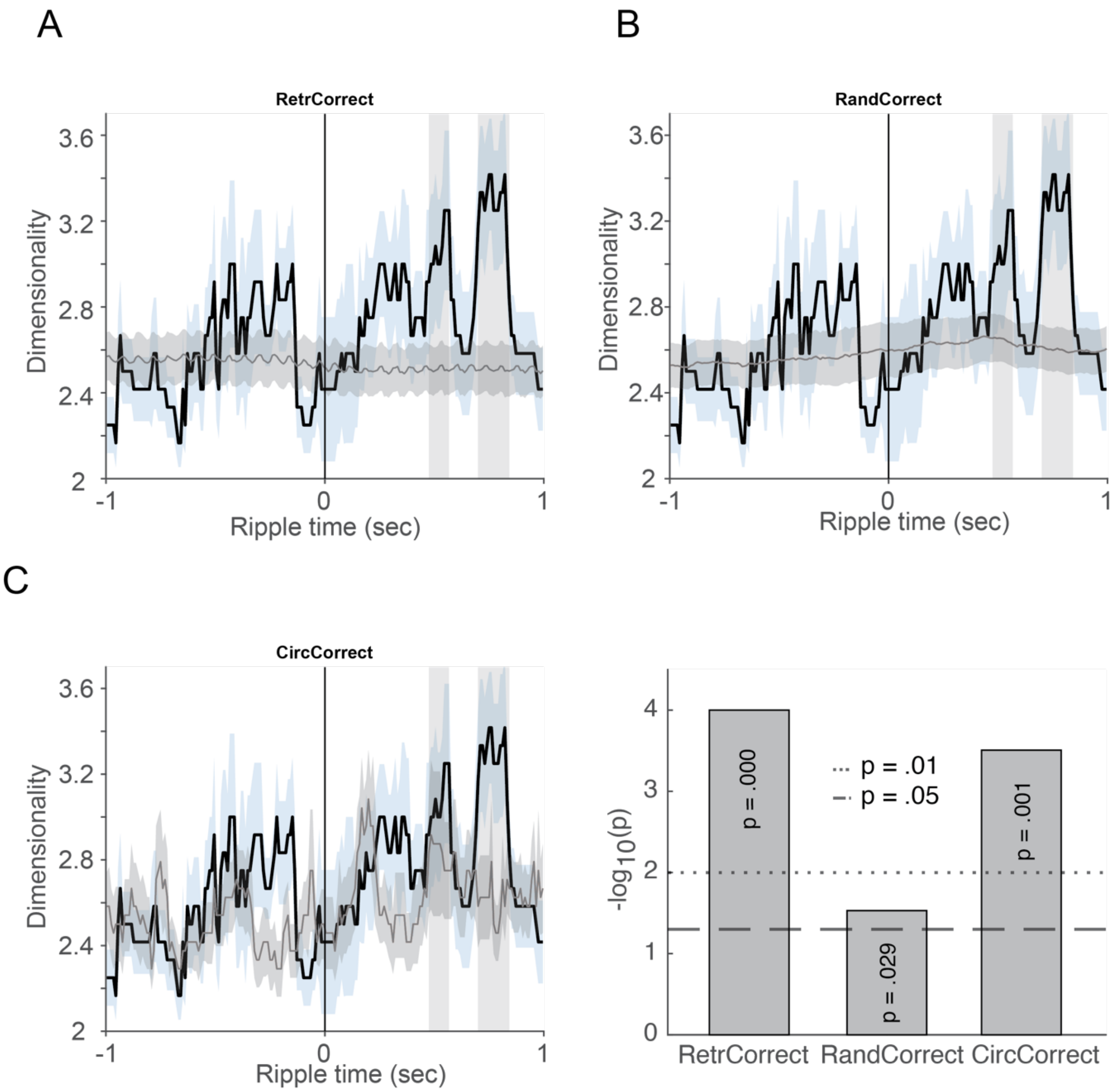

Control analyses confirming specificity of dimensionality expansion **(A)** From 1-5 seconds after cue onset, we picked 60 time points for each participant and treated them as ripple events and performed the same analysis as in 3B. **(B)** Shuffling AM+ trials 1000 times. **(C)** Empirical AM+ (blue) and adjacent trials (i.e. temporally shifted controls, grey). **(D)** P-values when comparing empirical data with the different controls.

3. Selecting the single largest ripple per trial does not seem that theoretically motivated, and potentially too arbitrary. Do the authors believe that reinstatement only occurs following the largest ripple? Do the authors believe that the chance of large ripples increases with ripple density? Does the size of the largest ripple systematically differ between AM+ and AM- trials, or with timepoint during retrieval (e.g do they tend to occur later)? How do all ripple qualities differ between AM+ and AM-, and how are these accounted for in comparing AM+ and AM- ripple-locked variables? For example, ripple length is the property associated with memory (PMID: 31197012).

We thank the reviewer for these thoughtful points. Our decision to select a single ripple per

trial, specifically, the largest, was motivated by methodological rather than theoretical considerations. We calculated the percentage of trials with zero, one or more than one ripple during retrieval. In brief, we found 1 ripple in on average 23% of the trials, no ripples in on average 21% of the trials and more than one ripple in 56% of the trials. Thus, many retrieval trials contain multiple ripple events, which introduces substantial temporal overlap between ripple-aligned windows, dependency between adjacent events, and confounds related to variability in ripple count across trials and participants. Selecting a single event per trial avoids these issues. We chose the largest ripple because it provides a consistent, high-signal-to-noise alignment point, but we do not assume that reinstatement occurs only after the largest ripple.

To empirically address the reviewer's concerns about ripple qualities between AM+ and AM- trials, we compared ripple properties. There were no significant differences in ripple size ($t(11) = .69$, $p = .50$), duration ($t(11)$ .65, $p = .53$), or density ($t(11) = .73$, $p = .48$). Thus, ripple characteristics do not confound the reinstatement effects we report.

Although ripple duration has been linked to memory strength in previous work (e.g., PMID: 31197012), duration did not differ between AM+ and AM- trials when we only picked one ripple per trial.

We thank the reviewer for highlighting this important point and have now added these controls to the Result section P. 5, l. 18 – P. 6, l. 2.

Minor issues and clarifications:
1. Can the authors cite a source on their automatic artifact rejection approach?

We thank the reviewer for this question. Our automatic artefact rejection approach follows widely used principles for iEEG artefact detection, combining absolute amplitude thresholds, sample-to-sample voltage gradients, and high-frequency activity (used to detect epileptogenic spikes). This multi-metric approach is consistent with established procedures in intracranial EEG preprocessing pipelines (e.g., Staresina et al., 2015, Hierarchical nesting, Vaz et al., 2019, Coupled ripple), all of which rely on z-scored amplitude, voltage derivatives, and high-frequency bursts to identify epileptiform or movement-related artefacts. We now cite these sources in the Methods (P. 20, l. 20).

2. How were false positive ripples identified for exclusion?

We used the built-in false-positive rejection step of our ripple-detection algorithm, configured to require a clear ripple-band spectral peak around each candidate event. For each putative ripple, we extracted a ±250 ms window around the event envelope maximum and computed a multi-taper time-frequency decomposition (FieldTrip, Hanning taper) from 65-135 Hz in 2 Hz steps, with frequency-dependent windows of ~100 ms. We then averaged power over a narrow window from −50 to +50 ms around the event and normalised each event's spectrum by its maximum. Events were retained only if the normalised spectrum contained at least one local maximum between 75 and 125 Hz whose prominence exceeded .2. Candidates that did not show such a prominent 75-125 Hz peak were classified as false positives and excluded from further analyses. Note that ripple detection and this spectral validation were applied

only to samples outside artefact segments (plus padding), so events overlapping interictal spikes or other large transients were removed earlier in the pipeline.

3. Why is ripple density a useful measure in the authors' analysis framework? They only end up using the largest ripple for subsequent analyses. Do they think cortical reinstatement is occurring relative to every ripple? Wouldn't that make it harder to resolve ripple-induced reinstatement in the authors' analysis pipeline when ripple density is higher?

We thank the reviewer for raising this point. Conceptually, we agree that retrieval is unlikely to be a single, punctate event triggered by one isolated ripple. Instead, the hippocampus often generates multiple ripples within a retrieval period, and these may each contribute to ongoing mnemonic processing. Indeed, recent human work suggests that ripple sequences or clusters may reflect sustained memory search or prolonged hippocampal-cortical interaction during retrieval (e.g., Vaz et al., 2020 - Replay of cortical spiking sequences). Ripple density therefore provides a useful index of the overall level of hippocampal engagement.

However, although ripple density is behaviourally and theoretically informative, it is less suitable as a temporal alignment variable for the analyses used in this paper. As we describe in response (3), aligning reinstatement or dimensionality directly to every ripple within a trial would substantially reduce temporal precision, create overlapping windows, and lead to non-independent samples, especially given that 56% of retrieval trials contained more than one ripple. This would make interpretation very difficult: reinstatement aligned to a given ripple would be contaminated by upcoming or preceding ripples within the same trial.

For this reason, we aligned neural data to a single ripple per trial, chosen as the ripple with the maximum envelope amplitude. This approach has two advantages: 1) It provides a consistent, high-signal anchor point for trial-wise alignment, and 2) It avoids double-counting reinstatement events that might co-occur within a ripple cluster, thereby preventing smearing of temporal effects.

Importantly, our choice to use the largest ripple does not imply that other ripples within the trial are irrelevant. Rather, we interpret ripple density as reflecting a continuous retrieval-related process, whereas the largest ripple serves as a clean temporal reference point around which fine-grained analyses (reinstatement, dimensionality, TG-PAC) can be reliably aligned.

Thus, ripple density is a useful descriptive and functional measure of hippocampal engagement, while using the largest ripple as the alignment point allows us to isolate the ripple-related cortical dynamics without contamination from other ripples within the same trial.

4. It is not totally clear to me how many trials were performed by each participant.

We thank the reviewer for this question and acknowledge that this was not included the previous version of the manuscript. Participants performed between 159 and 300 encoding trials and 300 and 450 retrieval trials (including new items). Lower numbers are for participants that couldn't continue the experiment due to either technical reasons or due to

fatigue. We have now included a Supplementary Table 1, where we list all the relevant characteristics per patient, including number of trials we analysed.

Table S1. Characteristics per patient.

Number of analysed trials is for one ripple per trial.

Patient	Implantation scheme	Number of analysed HC contacts	Number of analysed extra-HC contacts	Number of ripples	Number of analysed AM+ trials	Number of analysed AM- trials
1	Longitudinal	8	28	563	51	63
2	Longitudinal	7	59	1098	34	89
3	Longitudinal	7	28	907	48	109
4	Longitudinal	6	70	563	81	27
5	Longitudinal	6	37	1088	144	45
6	Longitudinal	6	53	356	48	46
7	Longitudinal	8	24	832	35	41
8	Longitudinal	7	54	2048	44	115
9	Lateral	2	61	220	31	29
10	Lateral	3	59	441	59	63
11	Lateral	4	81	304	42	19
12	Lateral	10	93	1028	87	38

5. Is it truly the case that encoding data is down-sampled and smoothed into low-frequency signal, but not the retrieval data? If so, is this meant to test compression of experience from encoding to retrieval? Can the authors clarify this point and better theoretically justify and/or empirically validate this analysis choice?

We thank the reviewer for pointing out this error in the previous manuscript. We have now changed on P. 22, l. 33. We down-sampled and smoothed both encoding and ripple-aligned data in a similar way.

6. What happens to the scale of PAC in Figure 4C (bottom) and 4D? The caption also mislabels 4E as 4F.

We thank the reviewer for spotting these confusions. Figure 4C (bottom) and 4D shows PAC compared to chance, therefore, being a very small number.

We have changed the caption label to now refer correctly to 4E.

Reviewer #2 (Remarks to the Author):

In the manuscript, Kerren et al. present new data on the interplay between hippocampal and neocortical dynamics during an associative memory task. Using intracranial electroencephalography (iEEG) recordings from 12 human patients with pharmaco-resistant epilepsy, the authors report: (i) a higher occurrence of hippocampal ripples during successful compared to unsuccessful retrieval; (ii) at retrieval, a reinstatement of neocortical activity patterns that were present during encoding, time-locked to hippocampal ripples; (iii) greater effective dimensionality of neocortical representations following ripples during successful, but not unsuccessful, retrieval; and (iv) increased theta-gamma phase-amplitude coupling (TG-PAC) during retrieval, occurring after the ripple and preceding the dimensionality expansion. Taken together, the study proposes that neocortical pattern reinstatement is supported by hippocampal ripples, which trigger TG-PAC and subsequently expand "compressed" cortical representations to facilitate successful associative retrieval. While the results are potentially of interest, the manuscript requires further clarification and additional controls to warrant publication.

Throughout the paper, the "unsuccessful retrieval" condition (AM-) combines both incorrect responses and trials in which patients recognized the image but explicitly reported uncertainty about the associated item ("don't know" responses, denoted "?"). Importantly, "don't know" responses constitute 67.5% of all AM- trials, whereas only 32.5% correspond to truly incorrect responses. Thus, the study effectively compares successful associative retrieval against a heterogeneous group dominated by uncertainty, not purely by retrieval failure. This is critical because the authors' main claim concerns processes specific to associative recognition memory rather than simple recognition, a particularly restrictive interpretation. Grouping "don't know" trials with incorrect ones introduces potential confounds, as many of the reported effects (e.g., ripple rate, cortical dimensionality, TG-PAC) might reflect participants' subjective certainty or uncertainty rather than accuracy per se. The authors attempt to justify this grouping (p.18, lines 25-26) by stating that "failure to recall is behaviourally equivalent to an incorrect memory judgement." However, these two cases differ in perceived confidence: in the former, participants recognize the image but are uncertain of the association, while in the latter they are confidently wrong. A more detailed characterization of these subcategories is therefore necessary to strengthen the interpretation of the results.

We thank the reviewer for raising this important concern regarding the heterogeneity of the AM- condition. As noted, AM- trials contain both incorrect responses and don't know responses, with the latter constituting the majority of AM- trials. This raises the possibility that some effects may reflect subjective uncertainty rather than retrieval failure per se.

To directly address the reviewer's concern, we conducted additional analyses treating incorrect and don't know trials as separate categories.

Dimensionality analyses.
Both AM+ vs. incorrect and AM+ vs. don't know comparisons showed significant increases in post-ripple cortical dimensionality (same window as Fig. 3B). That is, successful retrieval

reliably engaged higher-dimensional cortical states regardless of whether the comparison class was uncertainty-driven or error-driven.

Decoding analyses.
When comparing AM+ vs. don't know, we observed a significant decoding difference in the same post-ripple time window reported in Fig. 3A. When comparing AM+ vs. incorrect, we found a trend-level cluster (p = .09) in the very same time window. The temporal profile of both contrasts closely matched the original AM+ vs. AM- effect, indicating that the reinstatement differences are not specific to the "don't know" category.

Together, these results demonstrate that our main findings of enhanced reinstatement and increased dimensionality hold when don't know and incorrect trials are analysed separately. Guided by the reviewer's suggestion we have now included these analyses and the related figure in the revised manuscript (Supplementary Fig. 4 and below).

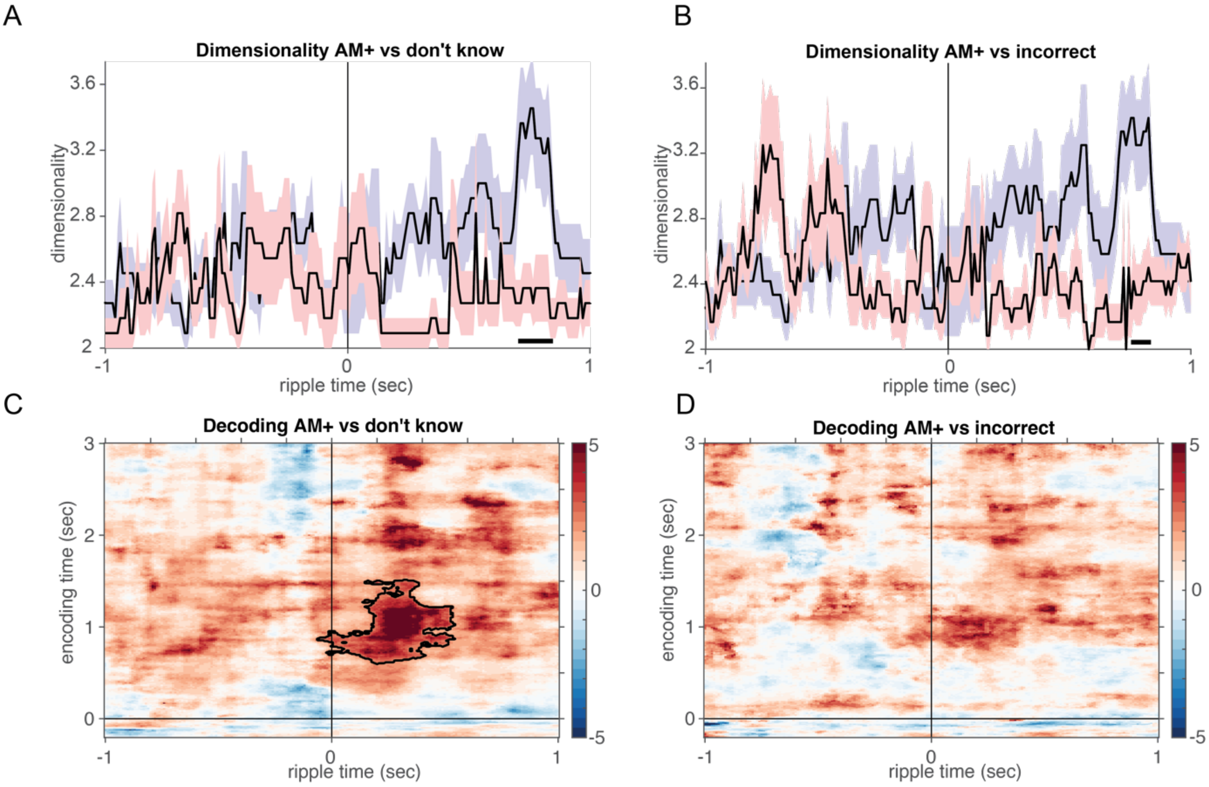

Dimensionality and decoding analyses using don't know and incorrect responses **(A)** Comparing AM+ to don't know responses yielded a similar significant time window as in Figure 3B. **(B)** The same was true when comparing AM+ to incorrect responses **(C)** We observed a similar decoding peak when comparing AM+ to don't know responses as in Figure 3A. **(D)** A numeric different with a peak in the same time window as in Figure 3A, when comparing AM+ to incorrect trials.

The manuscript provides detailed information on hippocampal electrode localization but does not specify the placement of neocortical contacts. Are all extra-hippocampal electrodes located within the temporal lobe, or do they span multiple cortical regions? Moreover, are neocortical contact sites comparable across participants? Clarifying the degree of overlap in neocortical coverage, and whether these regions are areas known to support associative learning, would provide important context for interpreting the findings. It would also be

informative to discuss whether the recorded neocortical sites are implicated in visual or semantic associative memory processes. Including a figure analogous to Fig. S1 to illustrate neocortical electrode locations would greatly enhance clarity.

We thank the reviewer for this comment and agree that a more detailed information of the neocortical contacts would be helpful and in addition to the coverage in Figure 1C, we have now included a supplemental figure 2 with extra-hippocampal contacts for each patient.

In addition to hippocampal depth electrodes, all patients had extensive extra-hippocampal coverage in temporal and parahippocampal cortex. Across participants, the highest coverage was observed in left inferior temporal gyrus (AAL atlas: Temporal_Inf_L; 84 electrodes in 12/12 patients), right parahippocampal gyrus (ParaHippocampal_R; 78 electrodes in 12/12 patients), left parahippocampal gyrus (ParaHippocampal_L; 76 electrodes in 12/12 patients), and right inferior temporal gyrus (Temporal_Inf_R; 76 electrodes in 11/12 patients). These regions are strongly implicated in visual and associative/semantic memory processing. Guided by the reviewer's suggestion, we have now added this information to the revised manuscript P. 19, l. 34 – P. 20, l. 2.

The reported increase in ripple rate during AM+ trials may be confounded by differences in response times, as AM+ trials had faster responses on average (1.9 s vs. 2.1 s for AM-; p. 4, lines 7-8). Given that the mean ripple rate across patients during retrieval is reported to range from 0.1 to 0.4 Hz, a 2-second response window implies that some trials likely contained no ripples, many contained one, and only a few contained more than one. In cases with a single detected ripple, response time could therefore strongly bias the computed ripple rate within retrieval epochs. To control for this potential artifact, the authors could (i) repeat the analysis after subsampling AM+ and AM- trials to match response-time distributions, or (ii) report the average ripple count within response windows rather than the rate.

We thank the reviewer for raising this important point regarding potential confounds introduced by reaction-time (RT) differences between AM+ and AM- trials. Because hippocampal ripples occur at a relatively low rate (approximately .1-.4 Hz), it is correct that shorter trials could, in principle, yield inflated ripple-density estimates when normalised by RT.

To address the reviewer's concern, we performed a set of additional analyses to directly assess whether the observed memory-related differences in ripple activity were driven by RT.

First, we examined raw ripple counts per trial (summing the number of ripples across hippocampal channels, and not averaging across channels as in main analysis), without any RT normalisation. AM+ trials contained numerically more ripples (mean = 2.784) than AM- trials (mean = 2.457).

We then conducted a mixed-effects regression predicting trial-wise raw ripple counts from memory condition (AM+/AM-) while statistically controlling for trial-specific RT, with subject included as a random intercept. Both a Gaussian linear mixed model and a Poisson generalised linear mixed model showed that memory condition remained a highly significant

predictor of ripple count after accounting for RT. In the Gaussian model, the condition term was significant (estimate = .473, t = 4.793, p < .01), and in the Poisson model the effect was even stronger (estimate = .195, t = 6.651, p < .01). These results demonstrate that the memory-related increase in ripple occurrence persists when RT is explicitly modelled as a covariate.

Moreover, we performed an RT-matched subsampling analysis, in which we equated the RT distributions of AM+ and AM- trials within each participant using quantile-based matching. Even with RT fully controlled in this way, AM+ trials continued to show significantly higher ripple counts (t(11) = 2.868, p = .015). This analysis ensures that the effect is not attributable to unequal opportunities for ripple detection across conditions.

Together, these convergent analyses demonstrate that the memory-related increase in hippocampal ripple occurrence is not an artefact of RT differences. Instead, the effect remains robust across absolute ripple counts, regression models that account for RT at the trial level, and RT-matched subsampling procedures.

We thank the reviewer for highlighting this important point and have now added this clarification to the manuscript and report the additional analyses in the revised Results section, P. 4, l.33- P. 5, l. 2.

P7, L32-35: “higher post-ripple dimensionality during AM+ trials was associated with faster response times …. linking representational expansion to … behavioural … markers of successful retrieval.”. Is their a relationship between when the dimensionality expansion occurred in a trial, and the reaction time of that trial?

We thank the reviewer for this insightful suggestion. In principle, the question of whether trial-by-trial variability in the timing of dimensionality expansion predicts trial-by-trial reaction time would be highly informative. However, in our current analysis pipeline, this relationship cannot be computed.

Specifically, our dimensionality estimates are derived after flattening the data across channels and across trials within each participant. This step is necessary to obtain stable covariance structure and reliable low-rank embeddings. As a consequence, the dimensionality metric is produced at the participant level, not at the single-trial level, and thus does not preserve the temporal dynamics of individual trials. Because dimensionality is not defined per single trial, we cannot relate the latency of dimensionality expansion to the reaction time of that same trial.

For the analysis in Fig 3A-C, how many retrieval trials contained >1 ripples? It is claimed in the methods that when this occurs, the ripple with maximum envelope is chosen. However, if all ripples instantiate cortical reinstatement, will the other ripple not contaminate the analysis? If there are few of these trials, can they be excluded?

We thank the reviewer for raising this point. Across all retrieval trials included in the dimensionality analyses shown in Fig. 3A-C, 21% of trials contained no ripples, 23% contained exactly one ripple, and 56% contained more than one ripple. Thus, multiple-ripple trials are

common in our dataset. We have now included these metrics in our Method section P. 22, l. 13-15.

In our main analysis, we aligned the neural data to a single ripple per trial, specifically the ripple with the largest high-frequency envelope. This ripple-centred approach means that we do not average over entire trials, but rather over local windows time-locked to the selected event. Additional ripples that occur earlier or later in the same trial will therefore not systematically "contaminate" the estimate of ripple-aligned reinstatement; at most, they add unsystematic noise.

Given that 56% of the trials contained more than one ripple, we deem it unfeasible to run the same analysis excluding these trials.

While Figures S3-S5 greatly bolster the author's argument, showing alternative measures of dimensionality would help
Page 7 lines 27-28: "Similar results were found using effective dimensionality".
Page 19 lines 26-27: "Almost identical results were observed when calculating the distance to a fitted power-law distribution across time".
These are stated but not shown. Please show this in a supplementary figure.

We thank the reviewer for pointing this out and agree that these analyses should of course be a part of the supplemental material. We have now updated Supplementary Fig. 6 to include all the alternative methods.

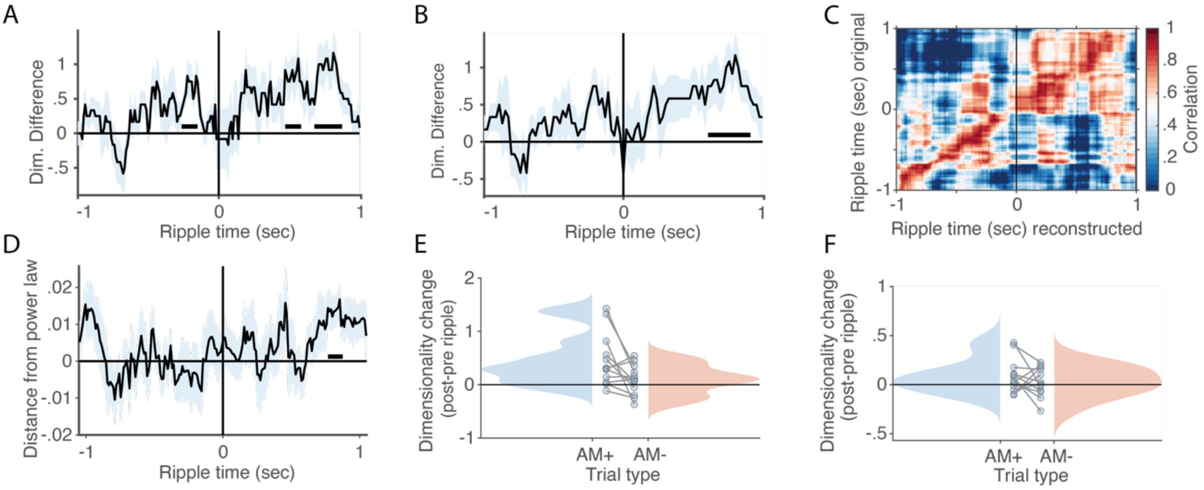

Robustness of dimensionality estimate, reconstruction analysis and alternative methods. Using a 100 ms **(A)** or 200 ms **(B)** sliding window for dimensionality estimation produced results highly similar to those obtained with a 60 ms window, demonstrating the robustness of the approach. **(C)** Correlation between original and reconstructed data, using the same number of components as in the PCA analysis, revealed a strong diagonal pattern, indicating a high-fidelity reconstruction of the original data structure. **(D)** Calculating the distance to a power-law distribution provided similar results as in 3B. **(E)** Estimating dimensionality using effective dimensionality provided similar results as in 3C. **(F)** Estimating dimensionality using Marčenko-Pastur threshold provided similar results as in 3C.

It should be further addressed why decoding performance from the components oscillates around ripple time (Fig 3D). While the manuscript primarily argues about the improved

decoding 400-800ms after the ripple, there are periods of significance roughly every 0.5s in Figure 3D.

We thank the reviewer for this thoughtful comment. As noted, the decoding time course in Fig. 3D displays slow oscillatory fluctuations around ripple onset, with peaks approximately every 0.5 s. Rather than reflecting alternating periods of improved and diminished reinstatement, this rhythmic structure likely arises from the fact that ripple timing is not random with respect to ongoing slow fluctuations in the hippocampal LFP.

Because this issue is closely related to the reviewers next question, regarding the possibility that ripple timing is aligned to a slow (~2 Hz) rhythm, we provide a full analysis and interpretation in our response to that question. In brief, we show that ripples in our dataset are indeed significantly phase-locked to slow (0.5-2 Hz) oscillations, consistent with previous findings in humans (Axmacher et al., 2008; Kunz et al., 2024). This rhythmic gating of ripple timing naturally produces the slow oscillatory structure visible in ripple-locked decoding curves. Crucially, however, as detailed in our response to Question 2, this slow-phase modulation cannot account for the selective post-ripple increase in decodability and dimensionality associated with successful retrieval.

In addition, dPC1 displays symmetric decoding performance before and after the ripple (Figure S6). This oscillatory pattern suggests that the computed decoding performance may be modulated by a slow (~2 Hz) brain rhythm. If so, ripple timing could be systematically aligned to this ongoing rhythm, and the observed relationship between ripples and neocortical dimensionality might arise from this coupling rather than retrieval per se. Additional control analyses are therefore needed to rule out this possibility.

We thank the reviewer for raising this interesting point. Indeed, previous work has shown that hippocampal ripple amplitude can be modulated by slow (.5-2 Hz) fluctuations, particularly in humans (Axmacher et al., 2008; Kunz et al., 2024), where ripples tended to occur preferentially on the descending phase of a delta cycle, consistent with the idea that ripple probability is gated by ongoing slow rhythms.

Importantly, however, the presence of slow-frequency modulation does not undermine our interpretation. First, hippocampal delta-ripple coupling has been shown to co-exist with the memory-related functions of ripples rather than replace them. That is, slow rhythms regulate when ripples occur, but do not by themselves produce reinstatement or retrieval-specific cortical dynamics. In our data, the delta-like oscillation visible in the dPC1 decoding trace simply indicates that ripple timing is not random with respect to slow fluctuations, but a phenomenon entirely consistent with prior literature.

To directly address the reviewer's concern, we calculated the absolute phase at the ripple peak and found very similar results to previous studies, with a mean phase of 37 degrees, which survived a phase-shuffled surrogate distribution, p = 0.019.

We thank the reviewer for highlighting this important point and have now added these results to Fig 2A and in the result section P.4,l.18-26.

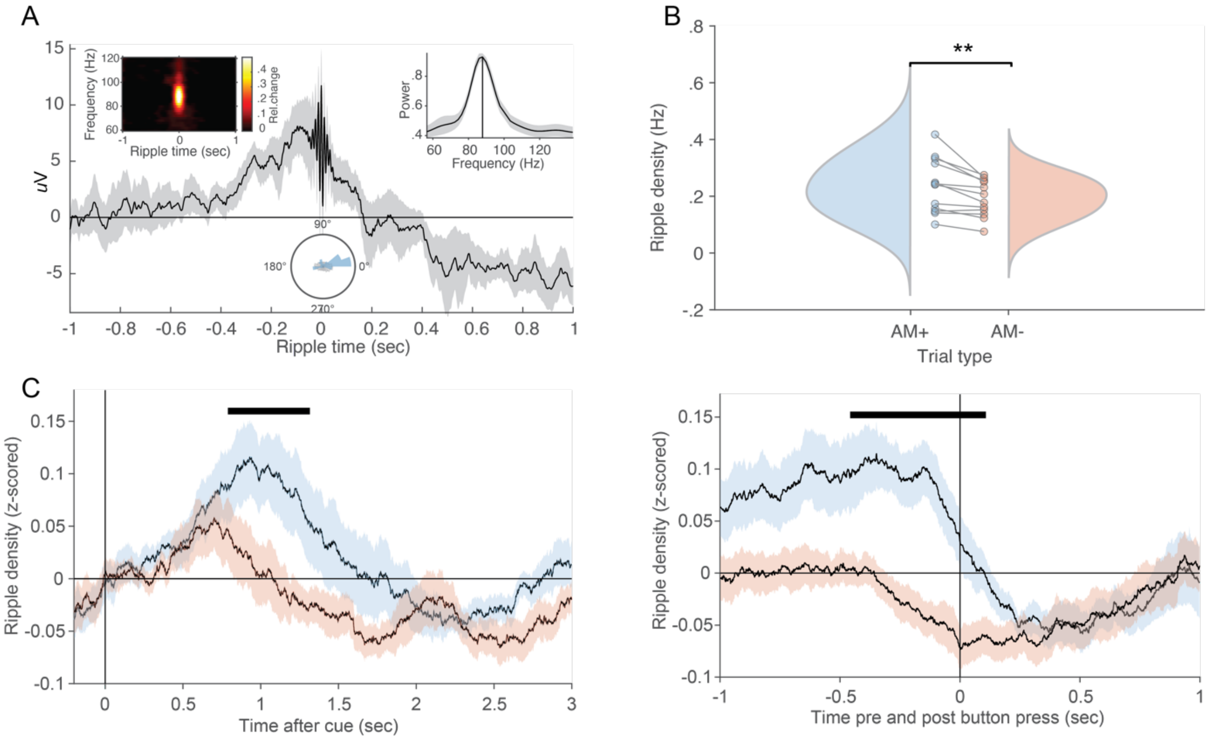

Revised Figure 2

In the same analysis, since the ripple tends to happen 1 second after the cue, and the dimensionality expansion 400-800ms after the ripple, does that not correspond to the time when the patient would have already or almost-already made their choice? Is the decodability of AM+ versus AM- in part due to the fact that the patient is very close to reacting at this point? Is choice time not factored into the analysis?

Several aspects of our design and analyses ensure that the observed AM+ vs. AM- differences are not driven by proximity to the button press or by generic motor preparation.

First, participants were required to press a button on every trial, regardless of whether their associative memory was correct, incorrect, or "don't know". Thus, motor preparation and execution were matched across AM+ and AM- conditions by design.

Second, we explicitly aligned all neural data to ripple onset, not to the response. Ripple events occurred at variable latencies relative to both cue onset and the button press. The distribution of ripple timings relative to response time was broad (median 925 ms post-ripple; i.e., it is unlikely that ripple-locked neural activity reflects fixed-time reaction-related processes), with 73% of the analysed ripples having more than 570ms (end of first cluster) between ripple peak and RT and more than half (54%) of the analysed ripples having more than 850ms (end of second cluster) between ripple peak and RT. We had further excluded all ripples that occurred 250ms before response Importantly, when we lock our analysis to equally variable delays with comparable distance to the motor response (circular shifted trials used as a control), we do not observe any significant cluster when comparing AM+ and AM-. Thus, ripple-locked neural activity cannot simply reflect fixed-time reaction-related processes.

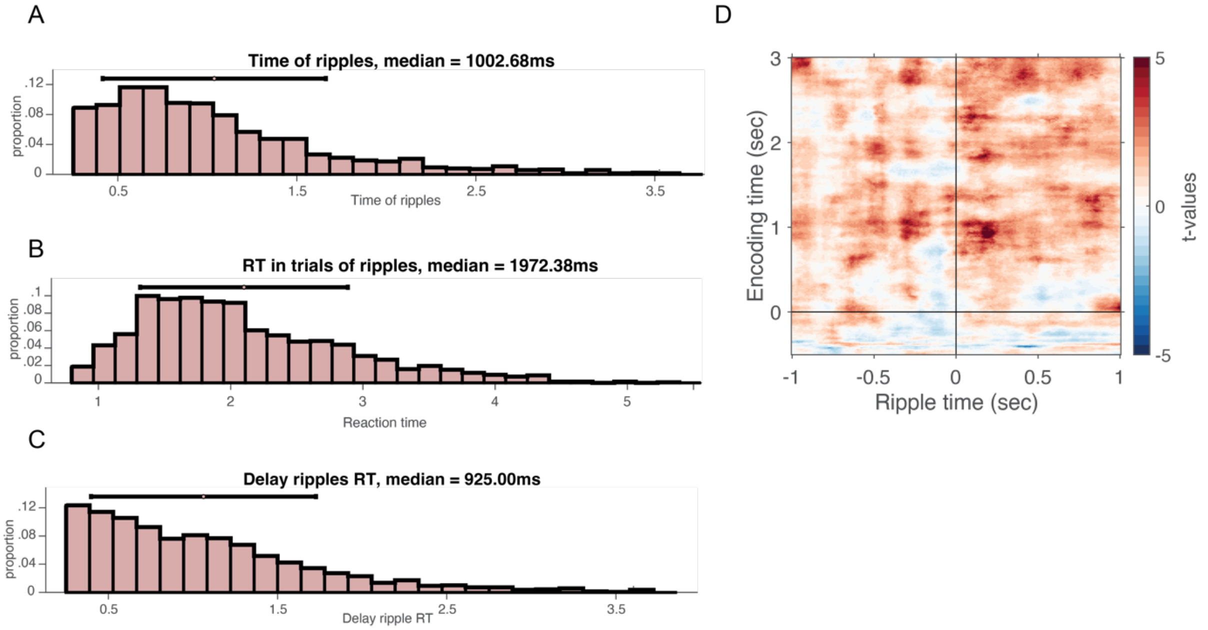

Distribution of ripple time and RTs. **A.** Median time for ripples was approximately 1002ms after cue onset, with a right-skewed distribution. **B.** Median RT was approximately 1972ms after cue onset. **C.** The median difference was approximately 925ms, again with a right-skewed distribution. **D.** When circularly shifting the trials one step, thereby keeping the time of the ripples and RT distributions, we found no significant decoding.

In demixed PCA, are components decomposed from different marginalizations guaranteed to be orthogonal in the original space, similar to components decomposed from the same marginalization (i.e the same matrix)? If not, the plot in 3E and S6H might be misleading.

We thank the reviewer for raising this important point regarding orthogonality of demixed PCA components. In standard dPCA, components extracted from within a single marginalization are orthogonal with respect to the covariance structure used for that marginalization. However, components extracted from different marginalizations are not constrained to be orthogonal in the original neural space, because each marginalization is decomposed independently (Kobak et al., 2016). Thus, stimulus-, condition-, and time-related components may exhibit non-orthogonal projections when plotted together.

In the present manuscript, the axes plotted in Figs. 3E and Supplemental Fig. 9H are not intended to convey orthogonality between marginalizations but rather to illustrate how single trials evolve along each demixed component. To avoid any possible misunderstanding, we have revised the figure caption to clarify that cross-marginalisation orthogonality is not assumed in dPCA and that the axes represent independent sources of variance but not necessarily orthogonal subspaces. Importantly, our conclusions do not rely on the orthogonality of components from different marginalisations.

In figure 3D, the dashed line shows the separability of clusters found via Kmeans, and the increase is suggestive of greater dimensionality. However, would it not be more appropriate to plot the separability (silhouette score) of clusters as defined and labelled by experimental variables, not Kmean clusters? Furthermore, it seems that some of the results of cluster

separation are reported in the Methods (P. 22 L30-33), which is a bit odd, these should be in the result section and potentially shown on Figure 3.

We thank the reviewer for this excellent suggestion. In the original submission we used K-means clustering to obtain a fully data-driven estimate of cluster separability. However, we agree that a complementary analysis based on the experimentally defined conditions (4 target associations × 2 memory outcomes) provides an alternative test of whether ripple events also increase the representational distinctiveness of task-relevant information. Thus, our K-means analysis addresses a complementary question: rather than assessing the distinctiveness of predefined conditions, it estimates how many distinct representational groups the neural activity supports in a fully data-driven way.

To directly test this prediction, we repeated the analysis using label-defined clusters. For each point in the dPCA state space (dPCs 1, 2, and 4), we computed the silhouette value using the fixed experimental labels rather than K-means assignments, and averaged silhouette scores across all points within the pre- and post-ripple windows. This analysis revealed a robust ripple-related increase in distinctiveness (pre: .632; post: .903; paired t-test: $t(327) = 13.03$, $p < .01$), demonstrating that cortical representations corresponding to the eight experimental conditions become significantly more distinct following ripple onset.

To further characterise the effect, we quantified label-defined separability separately for correct and incorrect trials. Both trial types showed a significant pre- to post-ripple increase (AM+: pre = .626, post = .915, $t(163) = 11.39$, $p < .01$; AM-: pre = .644, post = .892, $t(163) = 7.44$, $p < .01$). Importantly, the increase in representational separation was significantly larger for AM+ than AM- trials (difference AM- = 0.249; difference AM+ = .289; $t(163) = 2.56$, $p = .01$), indicating that ripple-triggered differentiation of cortical state space is most pronounced when memory retrieval is successful. The same pattern was observed when using dPC 1,3, and 4 instead.

These label-based results are an excellent alternative way of assessing distinctiveness and are therefore now presented in the main text, while we keep the unsupervised K-means results which address a complementary question. This analysis revealed an increase from 4-6 pre-ripple clusters to 8 post-ripple clusters, consistent with a ripple-triggered expansion of representational structure.

The reviewer also correctly notes that part of the clustering analysis was previously described in the Methods. In light of this, and following the addition of the label-defined clustering analyses, we have now moved all inferential results, including silhouette scores, pre/post comparisons, and statistical tests, into the Results section. The Methods section now contains only the procedural description of the dPCA projection and clustering pipeline. Together, these revisions ensure that all statistical inferences appear in the Results.

Fig 4D would benefit from some clarification: is PAC after ripple time-averaged? Or is the maximum taken? Same for dimensionality; is only the 400-800ms range used for dimensionality, while all post-ripple time is used for PAC?

We thank the reviewer for this comment and agree that we could have been more transparent on our method. To calculate the correlation between PAC and dimensionality, we averaged the result within the significant time points (highlighted with a horizontal black line) for PAC and dimensionality, separately. We then correlated the two resultant vectors. In the revised manuscript, we have explained this method in more detail (P. 13, l. 14-16).

P16, lines10-12: Eight patients had electrodes along the longitudinal axis of the hippocampus, while four had lateral implants (via the temporal lobe), likely capturing more of the transverse axis. Where there any systematic differences between these groups (i.e. did the longitudinal implants detect more ripples? How did that affect reported ripple rate?). Do the results qualitatively hold if these groups are analyzed separately?

We thank the reviewer for this insightful comment. Patients with lateral implantation exhibited a mean of 498 ripples, whereas those with longitudinal implantation showed a mean of 938 ripples. This difference is expected, as longitudinal depth electrodes typically contain more hippocampal contacts, increasing the likelihood of detecting events.

Unfortunately we only had 4 patients without longitudinal implantation scheme which is severely underpowered for group level statistical analysis. However we show that the effect holds when excluding these patients and that the effect is numerically the same in those patients.

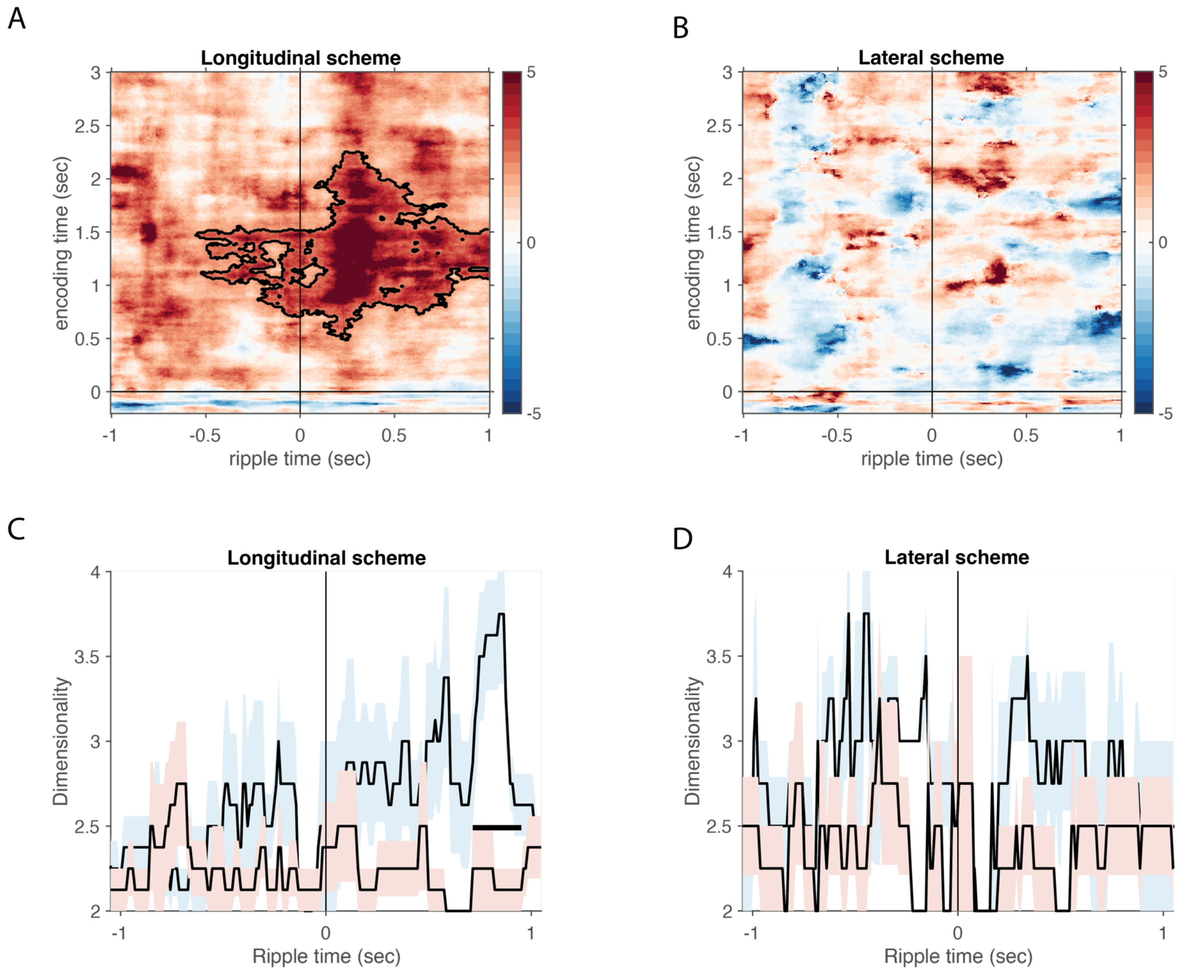

Reinstatement and dimensionality analyses using longitudinal and lateral implantation schemes. **(A)** A significant reinstatement cluster was observed for AM+ vs AM- in the longitudinal scheme **(B)** We did not perform any statistics for lateral implentation scheme due to too few patients, but nuemrically we saw the highest decoding difference in the same post-ripple time window. **(C)** Dimensionality was significantly higher post-ripples for AM+ vs AM- trials in the longitudinal implentation scheme. **(D)** We did not perform and statistics for dimensionality estimate, but observed a numerical increase following ripple onset for AM+ vs AM-.

Minor:

In general, more participant-specific information, possibly in the form of supplementary tables, would be helpful. For examples:

- P16 lines 28-32: a total of 72 hippocampal contacts across patients, and 647 extra-hippocampal contacts also across patients. But how many per-patient? How uniform or otherwise is the distribution ?

We thank the reviewer for this comment and agree that this is important information. We have now added a Supplementary Table 1, with a breakdown for each patient.

Table S1. Characteristics per patient.

Number of analysed trials is for one ripple per trial.

Patient	Implantation scheme	Number of analysed HC contacts	Number of analysed extra-HC contacts	Number of ripples	Number of analysed AM+ trials	Number of analysed AM- trials
1	Longitudinal	8	28	563	51	63
2	Longitudinal	7	59	1098	34	89
3	Longitudinal	7	28	907	48	109
4	Longitudinal	6	70	563	81	27
5	Longitudinal	6	37	1088	144	45
6	Longitudinal	6	53	356	48	46
7	Longitudinal	8	24	832	35	41
8	Longitudinal	7	54	2048	44	115
9	Lateral	2	61	220	31	29
10	Lateral	3	59	441	59	63
11	Lateral	4	81	304	42	19
12	Lateral	10	93	1028	87	38

- P3, line22 - page4, line8: AM+ and AM- numbers (and their breakdown) for different patients should be reported, as opposed to the average across patients.

We thank the reviewer for this comment and have now included a Supplementary Table 1, with all the characteristics.

- Figure S1: A comment on why the number of detected ripples in different patients is very variable, from ~400 events in some patients to >2,000 events in others. Possibly, knowing number of hippocampal contacts per patient would help.

We thank the reviewer for this comment and have also noted that there is a large difference between the patient with lowest and highest number of ripples. The patient with lowest number of ripples is the patient that only had two reliable contacts in hippocampus. We have now included a Supplementary Table 1 with all the necessary information per patient.

P8, Ll33-36: Incorrect reference to Figure 4A

Solved.

Labelling axes of Fig4B as theta and gamma would be helpful. Currently it must be inferred from the range of frequencies plotted.

Solved.

Since the dPCA decoding is an important part of the analysis In Fig3 and S6, a little clarification on how dpca_classificationAccuracy works in the methods would be useful. It is unclear to readers not familiar with the package.

We thank the reviewer for pointing this out and in the revised manuscript extended the explanation of the decoding procedure, P. 28, l. 11-34 and reads as following:

For decoding, we included the top 3 components for each marginalisation and repeated the classification 100 times using *dpca_classificationAccuracy*. To quantify how much task-relevant information was captured by the demixed components, we used the *dpca_classificationAccuracy* function from the original dPCA toolbox. This procedure performs cross-validated linear decoding on the dPCA projections. The function first computes the noise

covariance matrix across conditions, then runs dPCA on the training data to obtain decoder (W) and encoder (V) matrices for each marginalisation (e.g. target association, memory, their interaction, and time). For each cross-validation repetition, dpca_classificationAccuracy randomly assigns a subset of trials to a test set and uses the remaining trials to form a training set ("pseudo-ensembles"). The training data are projected onto the selected dPCs, and for each marginalisation and component, class means are computed along the relevant task dimensions (here corresponding to the four target associations, the two memory outcomes, or their interaction). The test data are projected using the same decoders, and at each time point the function assigns each test observation to the nearest class mean in this one-dimensional projection (minimum absolute distance). Classification accuracy is then computed as the proportion of correctly assigned conditions, averaged across repetitions and normalised by the number of conditions.

The output is an accuracy matrix with dimensions (marginalisation × component × time), where each element reflects how well that particular dPC discriminates between the predefined classes at a given time point. In our analyses, we focused on the first three components for each marginalisation (numComps = 3) and used the default cross-validation settings (100 repetitions). To assess statistical significance, these empirical accuracy time courses were compared against a label-shuffled baseline using the same decoding pipeline (see below for details on the permutation-based cluster correction).

Reviewer #2 (Remarks on code availability):no documentation and no code as far as I can tell, just data mat files

We thank the reviewer for pointing this out. In the original manuscript we did not provide links to the data and code (only in the submission form), which we apologise for. In the revised manuscript this is now included.

We also include the link here: https://github.com/kerrencasper/Hippocampal-ripples-initiate-cortical-dimensionality-expansion-for-memory-retrieval.git and 10.5281/zenodo.18490239

Reviewer #3 (Remarks to the Author):

The authors of this manuscript showed that cortical dimensionality increases immediately (<1s) after hippocampal ripples during memory retrieval in human subjects. After hippocampal ripples, but just before cortical dimensionality expansion, phase-amplitude coupling between hippocampal theta and cortical gamma emerges. While their findings are potentially interesting, I have several issues that should be addressed before the manuscript can be adequately evaluated.

1) For PCA-based dimensionality estimation, the authors determine the dimensions by identifying the “elbow” point at which the explained variance sharply declined across subsequent components. Is this method valid and used or supported by the literature? A more standard and widely used method to extract a statistically meaningful number of components identified by PCA is to determine the number of eigenvalues that exceed the Marchenko-Pastur threshold (Peyrache et al., 2009, Nat Neurosci 12:919-926). Since dimensionality estimation analysis is the basis of this manuscript, I suggest using this method to determine whether it and your method lead to the same conclusion.

We thank the reviewer for raising this important methodological point. We were not aware of that the Marchenko-Pastur (MP) distribution provides a principled way to identify statistically meaningful principal components. We are grateful for the suggestion to apply it here.

We implemented the MP procedure following Peyrache et al. (2009). After z-scoring each channel’s activity within a time window, we computed the channel × channel correlation matrix, extracted its eigenvalues, and determined the number that exceeded the MP upper bound. As the reviewer notes, eigenvalues above this bound reflect variance exceeding the level expected from random, uncorrelated data.

Critically, MP-based dimensionality estimation yielded the same conclusion as our elbow method when evaluating it using all blocks in a linear mixed effects model (significant interaction indicating greater ripple-induced expansion for remembered events (Estimate = -.083, SE = .030, t = -2.82, p = .0049). Dimensionality increased significantly following hippocampal ripples and this increase was larger for AM+ compared to AM- trials.

Thus, both approaches converge on the key finding that ripple events precede a behaviourally relevant expansion of cortical state space. We now include this MP-based control analysis in the Methods and in Supplementary Fig. 6F (also, see below).

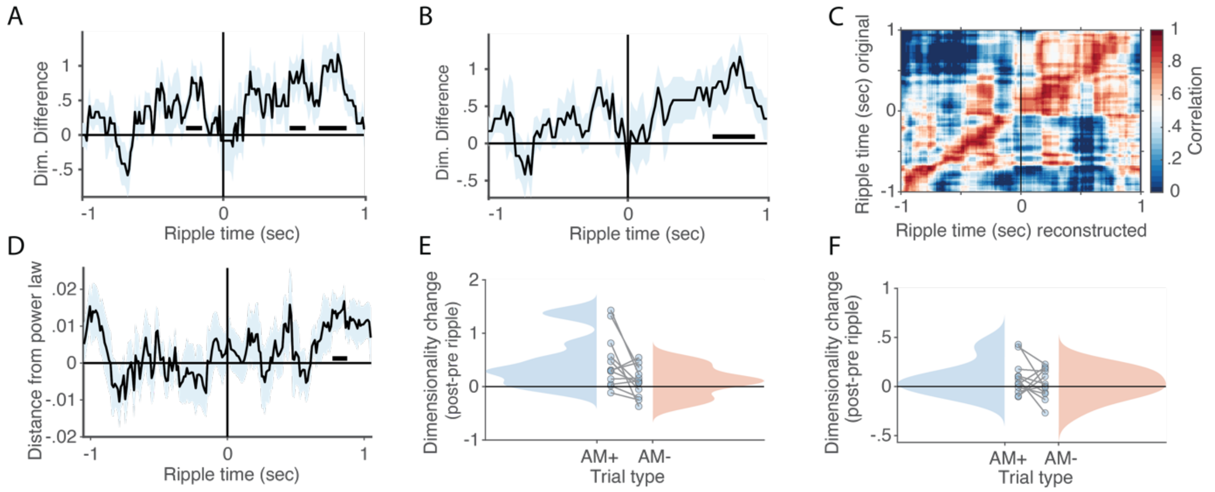

Robustness of dimensionality estimate, reconstruction analysis and alternative methods. Using a 100 ms **(A)** or 200 ms **(B)** sliding window for dimensionality estimation produced results highly similar to those obtained with a 60 ms window, demonstrating the robustness of the approach. **(C)** Correlation between original and reconstructed data, using the same number of components as in the PCA analysis, revealed a strong diagonal pattern, indicating a high-fidelity reconstruction of the original data structure. **(D)** Calculating the distance to a power-law distribution provided similar results as in 3B. **(E)** Estimating dimensionality using effective dimensionality provided similar results as in 3C. **(F)** Estimating dimensionality using Marčenko-Pastur threshold provided similar results as in 3C.

2) Hippocampal sharp-wave ripples occur during non-theta states and rarely occur concurrently with theta oscillations, at least in rodents (Buzsaki, 2015). The authors claimed that, immediately after ripples in the hippocampus, phase-amplitude coupling between hippocampal theta and cortical gamma emerges. Does this mean that ripples and theta oscillations occur concurrently in the human hippocampus? Or, does the brain state change immediately after ripples, entering theta-states? Alternatively, are the hippocampal ripples reported in this manuscript not bona fide sharp-wave ripples, but could they be gamma oscillations nested in theta oscillations in the hippocampus?

We thank the reviewer for raising this important physiological point. In rodents, sharp-wave ripples (SWRs) are well known to occur predominantly during non-theta states such as immobility and slow-wave sleep, and strong theta-ripple co-occurrence is rare (Buzsáki, 2015). However, several aspects of human intracranial electrophysiology differ from the rodent case and are essential for interpreting our findings.

First, a substantial body of work has demonstrated that awake human hippocampal ripples routinely occur during active cognitive processing, including episodic memory encoding and retrieval (e.g., Vaz et al., 2019; Norman et al., 2019; Zhang et al., 2021). In humans, therefore, theta and ripple activity are not mutually exclusive behavioural-state markers, and ripples are observed across a wider range of cognitive states than in rodents.

Second, our results do not indicate that theta and ripples overlap temporally within the same oscillatory event. The ripple waveform itself is brief (40-80 ms) and narrowband, and we did not detect theta power increases during the ripple (see figure below). Thus, our findings are consistent with the idea that ripples mark a transition point where they occur first, followed by the emergence of a theta-dominated hippocampal state that supports retrieval.

Third, our ripple detections are unlikely to reflect gamma oscillations nested within theta. We identified ripples using established criteria (band-limited 80-120 Hz energy, RMS-based envelope, stringent artefact rejection), and the events exhibited the spectro-temporal characteristics of canonical human SWRs. Additionally, we performed dedicated waveform-shape analyses on the cortical gamma signal, quantifying peak-trough sharpness, rise-decay asymmetry, skewness, and kurtosis, and found no evidence of the asymmetric or harmonic-rich waveforms expected if our ripple detections were contaminated by theta-nested gamma bursts. These shape metrics also did not correlate with peak gamma frequency, indicating that regional variations in gamma profiles cannot account for the observed PAC.

Taken together, these results support the idea that human hippocampal ripples do not occur within ongoing theta, but rather initiate a rapid transition into a theta-rich retrieval state, during which hippocampal theta couples to cortical high-frequency activity. This framework reconciles classical rodent SWR physiology with the well-documented presence of awake ripples in humans, while also providing a mechanistic account of how ripple-locked hippocampal output could coordinate subsequent cortical reinstatement.

In addition, we performed further analyses to understand the relationship between theta power and ripple events. Using one ripple per trial we estimated theta power in 3-8Hz, although we observe a general increase of theta power around the ripple peak, we find no difference in theta power between correct and incorrect trials (see fig below). We then correlated theta power with ripple amplitude. This correlation is very weak and does not survive correction for multiple comparisons across time. We also correlated number of ripples with theta power, and we observed a negative correlation around the ripple peak, which also did not survive correction across time. In sum, we find no relationship between theta power and memory around ripple events and also no relationship between theta power and ripple characteristics.

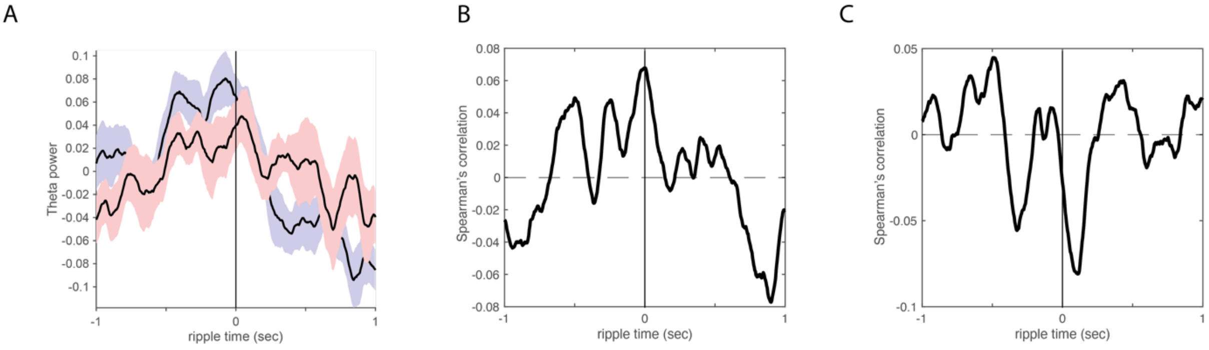

Relationship ripples and theta power. (A) *AM+ and AM- trials showed no difference in theta power around ripple events.* ***(B)*** *Correlation between ripple amplitude and theta.* ***(C)*** *Correlation between number of ripples and theta power.*

3) Which cortical areas were the iEEGs obtained from for cortical dimensionality estimation and analysis of hippocampal theta and cortical gamma phase-amplitude coupling?

We thank the reviewer for this comment and have in the revised manuscript included an additional supplemental figure illustrating all the cortical contacts included per patient as well as a Supplementary Table 1, for a breakdown for each patient.

Table S1. Characteristics per patient.

Number of analysed trials is for one ripple per trial.

Patient	Implantation scheme	Number of analysed HC contacts	Number of analysed extra-HC contacts	Number of ripples	Number of analysed AM+ trials	Number of analysed AM- trials
1	Longitudinal	8	28	563	51	63
2	Longitudinal	7	59	1098	34	89
3	Longitudinal	7	28	907	48	109
4	Longitudinal	6	70	563	81	27
5	Longitudinal	6	37	1088	144	45
6	Longitudinal	6	53	356	48	46
7	Longitudinal	8	24	832	35	41
8	Longitudinal	7	54	2048	44	115
9	Lateral	2	61	220	31	29
10	Lateral	3	59	441	59	63
11	Lateral	4	81	304	42	19
12	Lateral	10	93	1028	87	38

4) In summary, the authors claimed, “Ripple events also marked the separation of task-relevant variables in cortical state space,” however, I could not find the analysis and figures that support this claim. Please clarify.

We thank the reviewer for raising this point and appreciate the opportunity to clarify our claim. In the original submission, the statement that “ripple events marked the separation of task-relevant variables in cortical state space” referred to analyses performed on the dPCA projections, where we observed increases in the number of clusters and in the distances between clusters following ripple onset. These analyses were based on k-means clustering and were unfortunately described too briefly, with some inferential components appearing in the Methods rather than the Results, which made it difficult for the reviewer to locate the supporting evidence.

In response to this comment and in line with a related suggestion by Reviewer #2, we have now performed a clearer and more direct analysis using the experimentally defined task conditions complementing the k-means-derived cluster labels. This allows us to test explicitly whether ripples increase the representational separation of the eight task conditions (four target identities × two memory outcomes) in cortical state space. For each time point in the dPCA projection (using the three components carrying the most task variance: dPC1, dPC2, and dPC4), we computed silhouette values using these fixed labels and averaged silhouette scores within the pre-ripple and post-ripple windows. Silhouette values quantify how well points assigned to the same condition cluster together relative to how far they are from other condition clusters. Thus, higher silhouette scores indicate that the neural representations associated with different task-defined conditions (4 target identities × 2 memory outcomes) are more distinct and internally consistent in the state space.

This analysis revealed a robust ripple-related increase in representational distinctiveness: silhouette values increased from .632 before the ripple to .903 after the ripple (paired t(327)

= 13.03, p < .01). Thus, cortical activity corresponding to the eight experimental conditions becomes substantially more separable following ripple onset.

We further separated trials according to memory outcome. Both AM+ and AM- trials showed significant increases in silhouette values from pre- to post-ripple (AM+: .626 to .915, t(163) = 11.39, p < .01; AM-: .644 to .892, t(163) = 7.44, p < .01). Importantly, the magnitude of this increase was significantly larger for AM+ than for AM- trials (t(163) = 2.56, p = .01), indicating that ripple-triggered distinctiveness of cortical state space is strongest during successful associative retrieval. The same qualitative pattern was obtained when using the alternative set of dPCs (1, 3, and 4).

To improve clarity and transparency, we have now moved all inferential results related to clustering and separability, including silhouette scores, pre/post comparisons, and statistical tests, from the Methods into the Results section. The newly added label-based silhouette analysis is now presented in the main text and forms the main support for the claim that ripples increase the separation of task-relevant cortical representations. Importantly, the earlier K-means clustering analysis addresses a complementary question: rather than assessing the distinctiveness of predefined conditions, it estimates how many distinct representational groups the neural activity supports in a fully data-driven way. This analysis revealed an increase from 4-6 pre-ripple clusters to 8 post-ripple clusters, consistent with a ripple-triggered expansion of representational structure. We think that the label-based silhouette analysis provides a more direct test of task-relevant separability and therefore now present it in the main text alongside the K-means results as complementary evidence for a data-driven increase in cluster complexity.

5) Fig 3E, "Plotting dPCs 1, 2 and 4, showed overlapping representations of experimental variables before ripples (left), whereas their separability increased after ripples (right)." If dPCA was performed on the pre- and post-ripple events together and the results were shown separately using the same cPC1, 2, and 4 coordinates, then the results would be more interpretable. Otherwise, it is difficult to interpret the comparison between the pre- and post-ripple events.

We thank the reviewer for this comment and want to clarify that the dPCA was performed on the entire data from -1 to 1 second around ripples. For results and analyses, we divided the data into pre and post ripple. We have explained this in the revised manuscript on P. 9, l. 16.

6) How many contacts (recording sites) were used to estimate cortical dimensionality? The number of recording sites affects the number of estimated dimensions, which can make them difficult to interpret. This point should be discussed.

We thank the reviewer for this insightful comment. We ensured that an equal number trials (see P. 24, l. 22-23) and the same number of contacts were used to estimate dimensionality for both conditions. We further visualise this in the revised manuscript in Supplementary Table 1.

7) page 7, lines 27-28: "Similar effect were found using effective dimensionality". Since estimating dimensionality is probably the most important aspect of this manuscript, I

suggest presenting the results of effective dimensionality in supplemental figures.

We thank the reviewer for this comment and have now included the additional measures in Supplementary Fig. 6D-F and below.

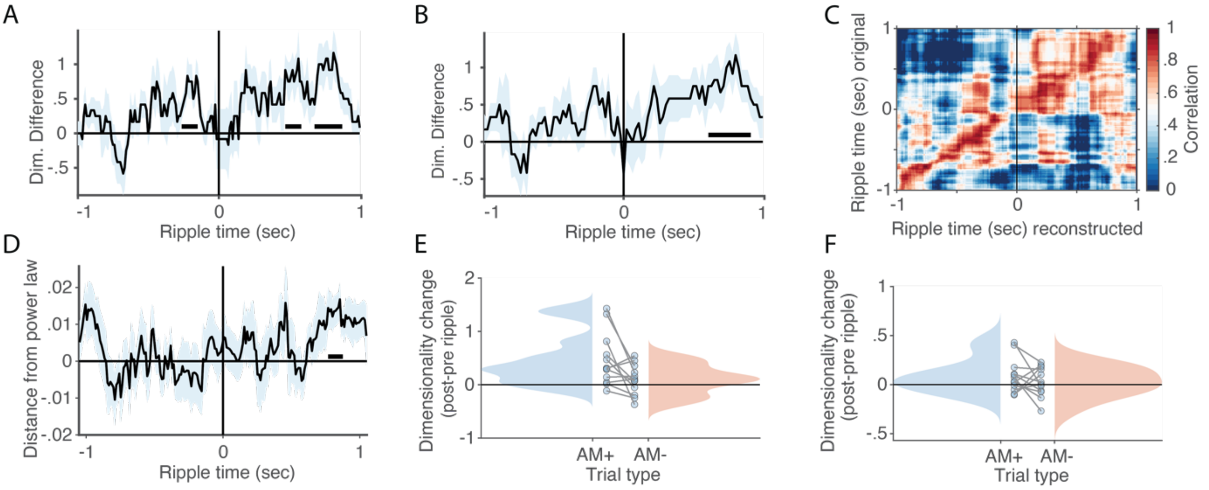

Robustness of dimensionality estimate, reconstruction analysis and alternative methods. Using a 100 ms **(A)** or 200 ms **(B)** sliding window for dimensionality estimation produced results highly similar to those obtained with a 60 ms window, demonstrating the robustness of the approach. **(C)** Correlation between original and reconstructed data, using the same number of components as in the PCA analysis, revealed a strong diagonal pattern, indicating a high-fidelity reconstruction of the original data structure. **(D)** Calculating the distance to a power-law distribution provided similar results as in 3B. **(E)** Estimating dimensionality using effective dimensionality provided similar results as in 3C. **(F)** Estimating dimensionality using Marčenko-Pastur threshold provided similar results as in 3C.

8) page 19, lines 26-27 "Almost identical results were observed when calculating the distance to a fitted power-law distribution across time." I could not understand this sentence or its relationship to the other sentences in the paragraph. Please clarify.

We apologise for not being very thorough on this point. We have now updated the revised version of the manuscript with the following:

To ensure that our dimensionality estimates did not depend on the elbow-detection procedure, we implemented an additional control analysis based on the deviation of each eigenvalue spectrum from a fitted power-law distribution. For each time window, we fitted a power-law function to the normalised eigenvalues of the covariance matrix using least-squares regression in log-log space (MATLAB fit, model power1). We then quantified the strength of the elbow by computing the signed difference between the empirical eigenvalue at the elbow (identified via the second-derivative method) and the eigenvalue predicted by the fitted power-law at the same component index. Large positive values indicate that the empirical spectrum bends more sharply than expected under a scale-free distribution, reflecting a stronger separation between signal- and noise-dominated components, whereas values close to zero indicate an eigenvalue decay well-approximated by a power-law.

The temporal profile of this measure closely paralleled the dimensionality time course obtained using the elbow method (Supplemental Fig. 6D): both showed a marked increase following ripple events, and both were significantly larger for AM+ compared with AM- trials. This convergence demonstrates that the observed ripple-triggered increase in representational complexity is not an artefact of the elbow-detection algorithm but reflects a

genuine change in the structure of the neural covariance spectrum.

9) page 19, lines 36-37 "In a follow-up analysis, we correlated the significant time points of dimensionality estimates with (1) …" It is unclear to me how the significant time points were identified. Please clarify.

We thank the reviewer for pointing this out. In the revised manuscript, we have now amended this paragraph on P. 25, l. 18-22:

In a follow-up analysis, per participant, we extracted the average dimensionality estimate within the significant time points (black horizontal line in Figure 3B) and correlated this resultant vector containing one number per participant with (1) participants' reaction times (correct trials only), and (2) decoding accuracy (i.e., classifier performance for correct minus incorrect trials) using two-sided Spearman's rank correlations.

10) page 20 lines 14-36, page 21 line 1-3. This section is difficult to understand and would benefit from the addition of explanatory figures.

We thank the reviewer for noting that the reconstruction section was difficult to follow. We agree that this part of the analysis is conceptually dense, so we have substantially revised the text to clarify each step of the procedure and to make the purpose of the reconstruction analysis explicit. The updated version breaks the workflow into shorter, more interpretable stages, explains why reconstruction is necessary for validating the dimensionality reduction, and more clearly describes how the resulting correlations should be interpreted.

Importantly, the reconstruction analysis is not a central result in itself but a validation step that ensures the PCA-based dimensionality estimates retain the task-relevant structure necessary for decoding. The revised text now clearly states this purpose, outlines the logic of the reconstruction using straightforward language, and explains how correspondence with the original decoding provides evidence that the reduced components preserve meaningful information.

These clarifications substantially improve readability without altering the analysis or its conclusions and can be found in the revised manuscript (P. 26, l. 31 – P. 27, l. 26) and reads as following:

To ensure that dimensionality reduction preserved task-relevant structure in the neural data, we performed a reconstruction analysis that quantified how much of the original decodable information was retained after projecting the data into the reduced PCA space.

First, for each participant we computed the within- and between-class covariance matrices and extracted the eigenvectors corresponding to the number of PCA components identified through the elbow method. We then reconstructed the neural activity by multiplying the class centroids for each condition with these eigenvectors, separately for encoding and retrieval. This produced a low-dimensional approximation of the original data expressed back in the full sensor space.

To evaluate how well the reduced components preserved class-specific information, we applied the same decoding procedure to both the original and reconstructed data. For each time point, we compared the projection of each trial to the reconstructed class centroids (colour vs. scene) and assigned the label corresponding to the closest centroid. This produced a time-generalisation matrix analogous to that obtained from the original data.

We then computed the Spearman correlation between the decoding matrices derived from original and reconstructed data. This resulted in a two-dimensional matrix capturing, for each retrieval time point, how closely its reconstructed decoding profile matched the original profile across all encoding time points (See Supplemental Fig. 6C). The diagonal of this matrix reflects correspondence at matched time points. To summarise this relationship at the participant level, we averaged across retrieval time, yielding one correspondence value per participant and trial type.

These values showed a strong and significant correlation across participants (Spearman's $\rho = .87$, $p < .001$), demonstrating that the PCA-based dimensionality reduction retained the majority of information relevant for distinguishing AM+ from AM- trials. Although decoding accuracy was used here as the validation metric, the same reconstruction procedure could be performed with any other measure of representational structure.

11) page 29, lines 4-6 "(B) The first four dPCs primarily reflected a mixture of "target association" and "memory" (dark blue), "target association only" (red), and "memory only" (beige)." It is unclear how the authors determined which dPCs associated with "target association," "memory," "target association only," and "memory only." Please clarify.

We thank the reviewer for requesting clarification on how we determined which demixed principal components (dPCs) corresponded to "target association," "memory," "target association only," and "memory only." We agree that this needed to be explained more explicitly.

We have now added a detailed description to the revised manuscript and clarified the caption to state that the assignment of components to marginalisations follows directly from the dPCA algorithm, which provides separate encoder/decoder matrices for each predefined marginalisation (target association, memory, interaction, condition-independent). Thus, the association between components and task variables is not inferred post hoc; it is explicitly given by the demixing procedure, as originally described in Kobak et al. (2016).

On page 9, lines 31-35, we have revised the text as follows:

Each dPC is assigned to the marginalisation (target association, memory, interaction, or condition-independent) whose decoder captures the greatest portion of its explained variance. Thus, the labels in Supplemental Fig. 9B ("target association," "memory," etc.) directly reflect the output of the dPCA algorithm and correspond to the marginalisation that best explains each component's variance.

Reviewer #4 (Remarks to the Author):

The authors present results from intracranial recordings suggesting that ripples play a crucial role in memory reactivation in humans. They analyse previously published data (Staresina et al., 2012, 2016) to show that following ripples: (i) memory decoding increases during successful retrieval, (ii) the dimensionality of cortical intracranial EEG rises during successful retrieval, and (iii) theta-gamma coupling increases, unrelated to memory.
The combination of dimensionality analyses with ripples is both innovative and intriguing. Unfortunately, in many instances, methodological details are insufficiently described or remain opaque, and several control analyses are unconvincing. The authors also tend to overstate some interpretations, employing causal language where it is not warranted.
I have outlined my principal concerns and other points below. The most pressing issue is that it remains unclear whether the detected ripples are physiological in nature or merely artefacts.

Major concerns:
A) Ripple detection is critical, yet it is uncertain whether the reported ripples reflect genuine physiological events, and whether the detection methods can be trusted.

We fully agree that ripple detection is critical, and we appreciate the opportunity to clarify the steps we took to ensure that the reported events reflect genuine physiological ripples rather than artefacts or filtering artefacts. We have now expanded the Methods section (P. 20, l. 28 – P. 21, l. 18) to explicitly describe each component of the pipeline and to improve transparency about the parameters used and the safeguards implemented:

Ripple events were detected independently in each hippocampal channel using a validated ripple detection algorithm. Channel-specific artefact segments, including epileptogenic spikes detected by an automated algorithm, were excluded from the analysis with an additional ±50 ms padding window. Only data from non-pathological hemispheres were analysed.

For each channel, the signal was bandpass filtered between 80 and 120 Hz using a two-pass FIR filter. A 20 ms RMS envelope of the band-passed signal was then computed and smoothed with a 20 ms moving window. Ripple detection thresholds were computed in a strictly channel-wise fashion using only artefact-free data: the main detection threshold was set to the mean ripple-band envelope plus 1.5 standard deviations, and an upper cutoff of mean + 9 standard deviations was applied to suppress large-amplitude transients during threshold estimation.

Continuous supra-threshold segments were then identified and retained only if they met standard physiological criteria. Events had to be between 38 and 500 ms in duration and had to contain at least three full oscillatory cycles (three peaks and three troughs) in the underlying raw or band-passed signal, ensuring that events reflected true high-frequency bursts rather than single sharp deflections. For additional confirmation, each candidate event underwent a false-positive rejection procedure based on its frequency profile: for every event, a time-frequency representation (65-135 Hz) was computed in a ±250 ms window around the envelope peak, and the spectral power was normalised across frequencies. Events were accepted only if they exhibited a prominent narrowband peak within 75-125 Hz, providing independent verification that the event was spectrally consistent with physiological ripple activity. Events failing any of the criteria above were discarded.

Together, these steps reassured that we found physiological ripples, rather than pathological ones or artifacts.

i) If these are indeed physiological ripple oscillations, one would expect to see a narrow-band peak in the power spectrum. Rather than relying solely on time-frequency plots—which can be biased by the authors' filtering approach—the authors should present non-time-resolved power spectra based on the retrieval data. Specifically, they should compute a simple Fourier transform over 0-3 s and display the group average in Figure 2, as well as individual patient spectra in Figure S1. Sometimes removing 1/f helps to find peaks in higher frequencies.

We thank the reviewer for this insightful comment. In line with the reviewer's suggestion, we computed non-time-resolved, 1/f-corrected power spectra centred on ripple events. For each hippocampal contact, we extracted data from -250 to +250 ms around ripple peaks during retrieval, estimated the fractal (1/f-like) and original spectra using the IRASA method (Wen & Liu, 2016) in the 50-250 Hz range, and obtained the oscillatory spectrum by subtracting the fractal from the original component. We then averaged these oscillatory spectra across ripples and contacts within each patient and across patients. The resulting spectra (now shown in Fig. 2A) exhibit a clear, narrow-band peak in the high-frequency range (~80-100 Hz, max peak for the average within the ripple band at 86.5Hz, SE = .47) for all but one patient with a maximum peak at 70Hz and only a secondary around 90Hz. This pattern confirms that the detected events correspond to narrow-band ripple oscillations rather than broadband high-frequency activity, and the peak frequency is consistent with the ripple frequency estimated from our time-frequency analyses (mean 89 Hz). These results are now included in Fig. 2A and Supplementary Fig. 1.

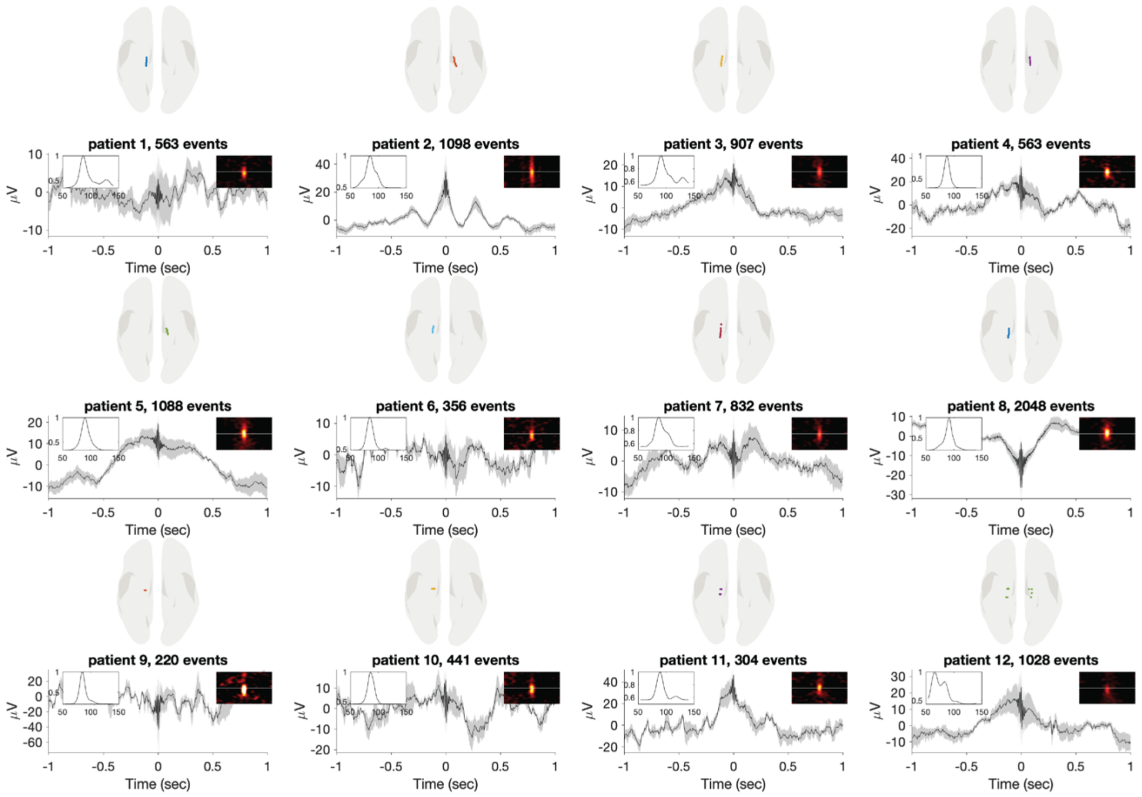

Revised Supplemental Figure 1.

ii) Merely citing literature instead of describing ripple detection methods in detail is inadequate for such a delicate and central issue. Moreover, two of the three referenced

papers are not even concerned with ripples. Please provide the details of the ripple detection approach.
Typically, ripple detection involves thresholding the root mean square signal, yet this is not reported. Were the authors not using this criterion? Given their description—bandpass between 80-120 Hz, 3 cycles, 38-500 ms—the detected “ripples” could represent many things, but not necessarily ripples.

We thank the reviewer for this point and apologise for not having included a thorough explanation of the method. We have updated the method section P. 20, l. 28 – P. 21, l. 18 and reads as following:

Ripple events were detected independently in each hippocampal channel using a validated ripple detection algorithm [95]. Channel-specific artefact segments, including epileptogenic spikes detected by an automated algorithm, were excluded from the analysis with an additional ±50 ms padding window. Only data from non-pathological hemispheres were analysed.

For each channel, the signal was bandpass filtered between 80 and 120 Hz using a two-pass FIR filter. A 20 ms root-mean-square (RMS) envelope of the band-passed signal was then computed and smoothed with a 20 ms moving window. Ripple detection thresholds were computed in a strictly channel-wise fashion using only artefact-free data: the main detection threshold was set to the mean ripple-band envelope plus 1.5 standard deviations, and an upper cutoff of mean + 9 standard deviations was applied to suppress large-amplitude transients during threshold estimation.

Continuous supra-threshold segments were then identified and retained only if they met standard physiological criteria. Events had to be between 38 and 500 ms in duration and had to contain at least three full oscillatory cycles (three peaks and three troughs) in the underlying raw or band-passed signal, ensuring that events reflected true high-frequency bursts rather than single sharp deflections. For additional confirmation, each candidate event underwent a false-positive rejection procedure based on its frequency profile: for every event, a time-frequency representation (65-135 Hz) was computed in a ±250 ms window around the envelope peak, and the spectral power was normalised across frequencies. Events were accepted only if they exhibited a prominent narrowband peak within 75-125 Hz, providing independent verification that the event was spectrally consistent with physiological ripple activity. Events failing any of the criteria above were discarded.

Together, these steps reassured that we found physiological ripples, rather than pathological ones or artifacts.

iii) Furthermore, the authors report power peaks around 60 Hz in their CFC analyses. How can they be confident that the ripples are narrowband, given multiple other gamma-range power peaks?

We thank the reviewer for raising this concern. Ripple detection was done on hippocampal channels, whereas power estimates was done on cortical channels, where we were agnostic about whether it was ripples or gamma power. Specifically, the PAC analysis was explicitly designed to test long-range coupling between hippocampal theta phase and cortical gamma amplitude. Thus, the gamma-frequency peak observed in Fig. 4 originates from cortical channels rather than from the hippocampal contacts used for ripple detection. Ripple detection, by contrast, was performed exclusively in hippocampal electrodes and only within

the canonical ripple band (80-120 Hz), using a multi-stage pipeline that included strict spectral false-positive rejection based on ripple-centred time-frequency representations (As also shown in response to reviewer's comment (A,i), we find a narrow-band peak in the power spectra for each participant). The presence of cortical gamma activity around 60 Hz does not influence the ripple detection process, nor does it affect the spectral verification of ripple events. Thus, the gamma peak reported in the PAC analysis is anatomically distinct from hippocampal ripples and does not undermine the conclusion that the detected events constitute genuine physiological ripple oscillations.

B. Unclear contribution of epileptic activity to "ripples"
The authors claim to use "an appropriate algorithm capable of detecting epileptogenic spikes" but provide no details. This is not sufficient. They must specify the algorithm, parameter settings, and confirm (ideally via visual inspection by a trained neurologist) that it performed appropriately on the individual patient level. They should also report how much data were classified as epileptic artefacts. Analysing only non-pathological hemispheres does not address the issue, since interictal activity often occurs outside the epileptogenic zone. To substantiate their findings, the authors should detect epileptic spikes during relevant periods using an automatic method similar to their ripple detection, then repeat their main analyses using those spikes. They should report:
- Epileptic spike-triggered averages
- Memory contrasts of spike density (cf. Fig. 2)
- Reactivation analyses based on spikes (cf. Fig. 3A)
- PCA analyses based on spikes (cf. Fig. 3B,C)
These results should appear in the supplementary material incl. figures, so readers can properly assess the main findings.

We thank the reviewer for raising this important point regarding the potential contribution of epileptiform activity to our ripple-locked findings, and for detailed guidance on how to rule out this potential confound. We now implemented this comprehensive control pipeline to detect, quantify, and analyse epileptic spikes, and we report the results in the revised Supplementary Materials.

Our artefact rejection pipeline is intentionally conservative and designed to remove a broad class of transient noise events, including, but not limited to, epileptiform spikes. The procedure detects large-amplitude deviations, sharp voltage gradients, and broadband high-frequency transients, all of which are characteristic of pathological spikes but also of non-physiological artefacts such as movement or electrical noise. Thus, while the method effectively removes most spike-like events, it should not be interpreted as a dedicated epileptic-spike detector. In response to the reviewer's request, we therefore implemented an additional spike-specific analysis

Since we used this method to detect artefacts, we include results where we quantified the fraction of all data points in a trial that fell within an automatically detected artefact segment, summed across channels and normalised by the total number of channel × time samples in that trial. A value of 1 would suggest that every channel-sample in that trial was marked as an artefact. Across participants, only .09% of channel×time samples within ripple-

locked retrieval trials overlapped with artefact segments (SE = .03%), indicating low levels of contamination.

To identify interictal epileptiform discharges (IEDs), we applied a previously used outlier-based spike detection algorithm (Honey et al., 2012; Michelmann et al., 2021), in which transient high-amplitude deflections are detected as samples exceeding a robust amplitude threshold relative to each channel's distribution. Specifically, for each hippocampal channel, we computed the channel median and interquartile range (IQR) over the analysed recording segment and flagged candidate spike samples whose absolute deviation from the median exceeded 2 × IQR, considering both positive and negative deflections. To remove the detected events from the continuous signal, candidate spike samples were temporally dilated by ±10 ms and the affected samples were replaced using piecewise cubic interpolation from the surrounding non-spike samples. For alignment analyses, we collapsed each suprathreshold segment to a single peak sample by selecting, within each contiguous suprathreshold run, the sample with maximal absolute deviation from the channel median. To avoid counting high-frequency burst activity as multiple spikes, we imposed a 100 ms within-channel refractory period, retaining only the first detected peak within each refractory window. Spike-triggered averages were computed by extracting ±100 ms epochs around each spike peak. Epochs were high-pass filtered at 5 Hz and baseline-corrected by subtracting the mean voltage in a pre-spike window (−100 to −20 ms) prior to averaging across spikes and channels.

As requested, we computed spike-triggered averages from hippocampal channels. We found no difference between number of spikes for AM+ and AM- trials ($t(11) = -1.15$, $p = .27$. This indicates that epileptiform activity is not selectively elevated on remembered vs. forgotten trials.

We repeated the full reinstatement analysis (Fig. 3A), aligning data to spike peaks instead of ripple peaks. No significant cluster emerged for spike-aligned reinstatement, contrasting AM+ and AM- trials. This stands in sharp contrast to the robust reinstatement observed when aligning to ripples. Finally, we repeated the PCA-based dimensionality analyses (Fig. 3B) using spike alignment. Again, we found no significant increase for AM+ trials compared to AM- trials.

Across all analyses requested by the reviewer, number of spikes for AM+ and AM-, reinstatement and PCA epileptic spikes failed to produce any of the neural signatures we observe around ripples. This demonstrates that the ripple-locked effects in reinstatement and dimensionality cannot be attributed to epileptiform artefacts.

The above analyses and related figures are now added to the revised manuscript (Supplementary Fig. 10 and 11) and can also be seen below.

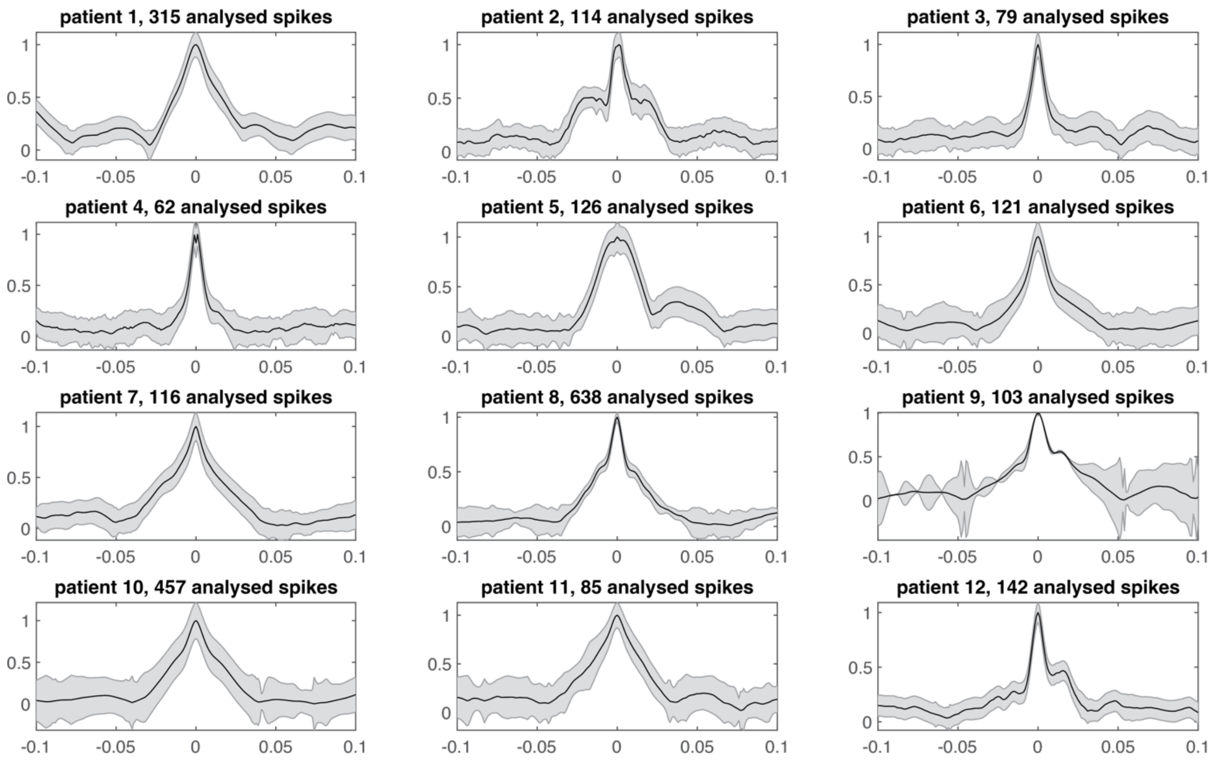

Spike-triggered averages. *For each participant, we extracted and averaged spikes, with the number of spikes above each spike-triggered average.*

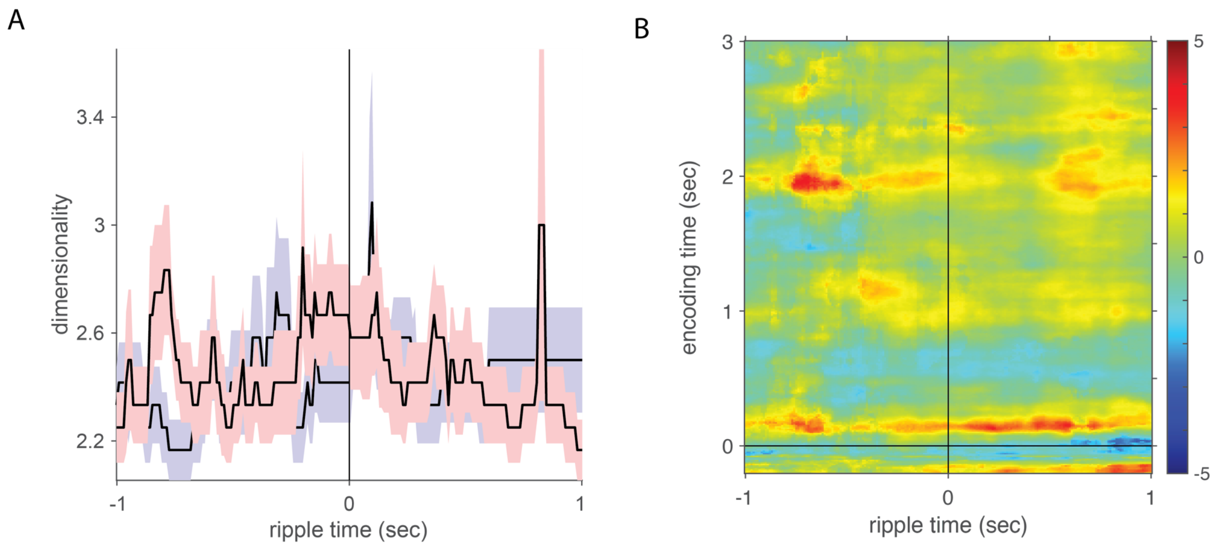

Dimensionality and decoding analyses based aligned on spikes. (***A***) Dimensionality analysis as in Fig. 3B showed no difference between AM+ (blue) and AM- trials (red). (***B***) Decoding analysis as in Fig. 3A showed no difference between AM+ and AM- trials.

C. Control analyses are not meaningful. The authors' approach attempts to control for significant effects during a specific time window, yet they never explicitly test for time windows. Instead, what their statistics in the main part do is to indicate whether two conditions are different - but not when exactly (see Maris & Oostenveld, 2007, J. Neurosci. Methods).

For instance, Fig. S5 "... when averaging across the significant time points identified in Fig. 2B…" is problematic, as it overinterprets a temporal cluster from the permutation test of the main analysis. Proper control would allow clusters without constraining time in the shuffled data, and then assess whether the occurrence of a significant cluster in shuffled data is equally probable.

The same issue applies to results presented in Fig. S2 ("…decoding accuracy within the significant cluster identified in Fig. 2A…"). Instead, they should test condition differences in their control analyses without temporal constraints, mirroring the main analyses. They should also compare shuffled AM+ and AM- data to determine whether their observed differences could be found by chance.

We thank the reviewer for this thoughtful comment and we agree that we have overstated the interpretation of the control analyses. Reviewer #1 comment "across analyses c" raises the same concern and we have addressed this by using the suggested control analysis. Here we reiterate the procedure.

To assess whether ripple onset provides a uniquely effective alignment point relative to all other moments in the retrieval epoch, we have now implemented an additional control analysis in which we, for each participant, computed reinstatement/dimensionality to 60 hypothethical ripple events, from 1 to 5 seconds after cue onset at retrieval. We epoched the data at each time point, allowing 1 second before and after, exactly the way we did it in the main analyses for Fig 3A-B.

We then subtracted the mean of these trials and subtracted by the standard deviation and ran a cluster-based permutation analysis on the z-value. This provides an explicit test of whether ripple onset is statistically privileged. We now report this analysis in the revised manuscript, and it yielded a significant cluster in the same time window as AM+ vs AM- reported in 3A (see Supplemental Fig. 5A, inset). For dimensionality analysis, we found a significant cluster ($p < .05$) in the same time-window (~700-800ms after ripples) as in Fig. 3B. We thank the reviewer for this insightful comment, which has strengthen our case.

In addition to this, we have revised our method for assessing significance within the found cluster in Figure 3A. We followed the statistically validated procedure in Stelzer et al (2013). Empirical values were first averaged within the time-frequency cluster identified in the main analysis, yielding a single cluster-mean value per participant. A group-level null distribution was then constructed by repeatedly sampling one surrogate estimate per participant from the corresponding control distribution and averaging these values across participants over 10 000 iterations. The empirical group-mean cluster value was compared against this null distribution to obtain a two-sided p-value.

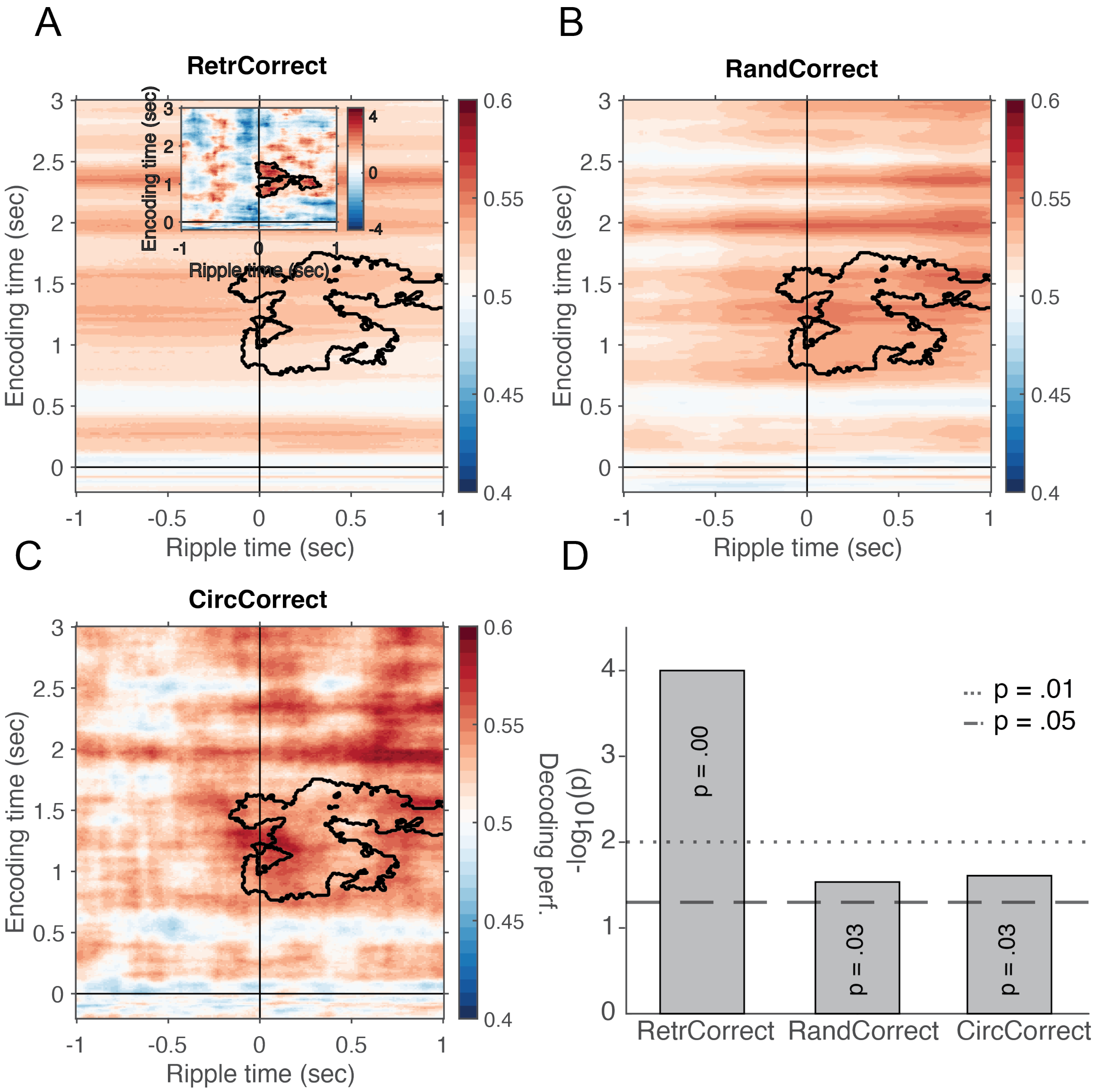

Control analyses confirming ripple-triggered reinstatement specificity. **(A)** From 1-5 seconds after cue onset, we picked 60 time points and treated them as ripple events and performed the same analysis as in 3A. The time generalisation matrix shows the decoding accuracy for these events with the significant cluster identified in Figure 3A highlighted in black. Inset shows a cluster-based permutation between the permuted baseline and empirical data, with a significant cluster in the same time window as in Figure 3A. **(B)** Decoding accuracy when AM+ trials were shuffled 1000 times. **(C)** A similar analysis was performed using a stricter control in which ripple times were swapped with those of adjacent trials (i.e., neighbouring trial shuffling). This revealed significant higher decoding in the empirical AM+ condition compared to the adjacent-trial control. **(D)** P-values when comparing empirical data with the different controls.

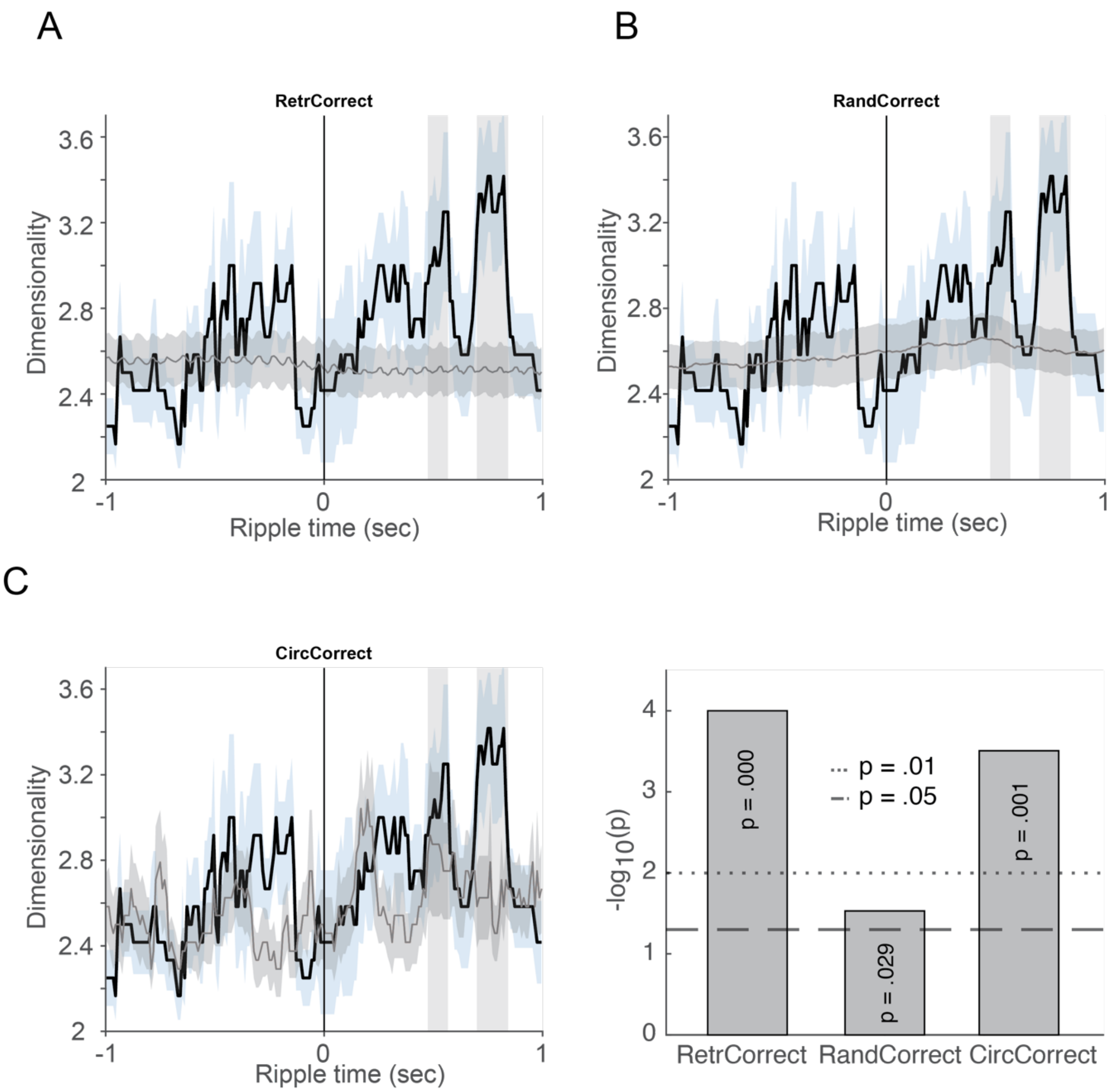

Control analyses confirming specificity of dimensionality expansion **(A)** From 1-5 seconds after cue onset, we picked 60 time points for each participant and treated them as ripple events and performed the same analysis as in 3B. Grey is permuted baseline, blue is empirical data. **(B)** Shuffling AM+ trials 1000 times. Grey is permuted baseline; blue is empirical data. **(C)** Empirical AM+ (blue) and adjacent trials (i.e. temporally shifted controls, grey). **(D)** P-values when comparing empirical data with the different controls.

Major points

1) How can the authors ensure that reactivation is not driven by the similarity between reading the identical word at encoding and retrieval? Patients' attention can fluctuate drastically in the hospital setting, which could lead to reading in one trial, but not reading in another ("forgetting"). Reading elicits stereotypical cortical activity. As Fig. 1b suggests, electrodes cover lateral temporal and frontal regions—likely language areas—whose timing could plausibly match the observed reactivation patterns (~500 ms post onset). A similar argument could be constructed regarding the dimensionality analyses based on this contrast.

We agree that, in principle, any encoding-retrieval similarity measure could be contaminated by stereotyped responses to visually identical word cues. We believe this is unlikely to explain our findings for several reasons, which we now clarify more explicitly below.

First, our reinstatement and dimensionality analyses target associative content, not simple word identity. At encoding, each word is paired with a category-level feature (colour: red/blue; scene: indoor/outdoor), and multiple different words are associated with the same colour or scene image. Classifiers and dimensionality estimates are therefore defined over the associative condition (e.g. indoor vs outdoor, AM+ vs AM-), not over individual words. Thus, the classifiers were trained on indoor and outdoor scene images or blue and red images. Every class of the four images were paired with multiple words, does making the classifier agnostic to the word, but predictive of the image class. Low-level visual or orthographic responses to the written cue are shared across trials and do not systematically distinguish which colour or scene was imagined. If reinstatement were driven purely by reading the word, it should not selectively recover which association was formed and decoding performance should not differ between AM+ and AM- trials.

Second, all critical retrieval analyses are ripple-locked, not cue-locked. Any stereotyped word-reading response that is tightly time-locked to cue onset (e.g. the 200-400 ms activity typically seen in language studies) will therefore be misaligned across trials in ripple-locked coordinates and will smear out rather than produce a consistent peak at a fixed ripple-aligned latency. By contrast, we observe a reliable increase in category-specific reinstatement and dimensionality after ripple onset in AM+ trials. This temporal relationship to ripples, rather than to cue onset, is difficult to reconcile with an explanation based purely on reading the cue.

Finally, we note that word reading is required on all retrieval trials (participants have to decide whether the word is new, old but not remembered, or old with a specific association). While attention can certainly fluctuate, a not-reading explanation of forgetting would predict performance failures and highly atypical RT patterns, which we do not observe. Moreover, ripples took place on average 1 second after cue onset that is classically associated with memory retrieval processes (e.g., Staresina & Wimber, 2019) rather than early sensory processing.

2) The reactivation in Fig. 3A spans 0-1 s after ripple onset and shows elevated t-values throughout encoding, also pre-ripple. The authors provide a mask that outlines the cluster from the permutation statistics, but this is not a good indicator for when reactivation actually occurs. They must present unmasked grand-average data to reveal temporal peaks, and test whether ripple-locking is indeed critical by repeating decoding on stimulus-locked data.
From the brief description in the results, it is very unclear whether the reactivation has any temporal relationship with the occurrence of ripples.

We thank the reviewer for this insightful comment. In addition to the results provided in "major concerns, C", which directly addresses this concern, we also ran the decoding analysis on stimulus-locked data. Although there seems to be an increase in decoding performance around 1.5-2.5 seconds for the encoding and retrieval axes, it did not survive cluster-based

permutation test.

Below we have included the unmasked grand average data, which are also added to the revised manuscript Supplementary Fig. 3 .

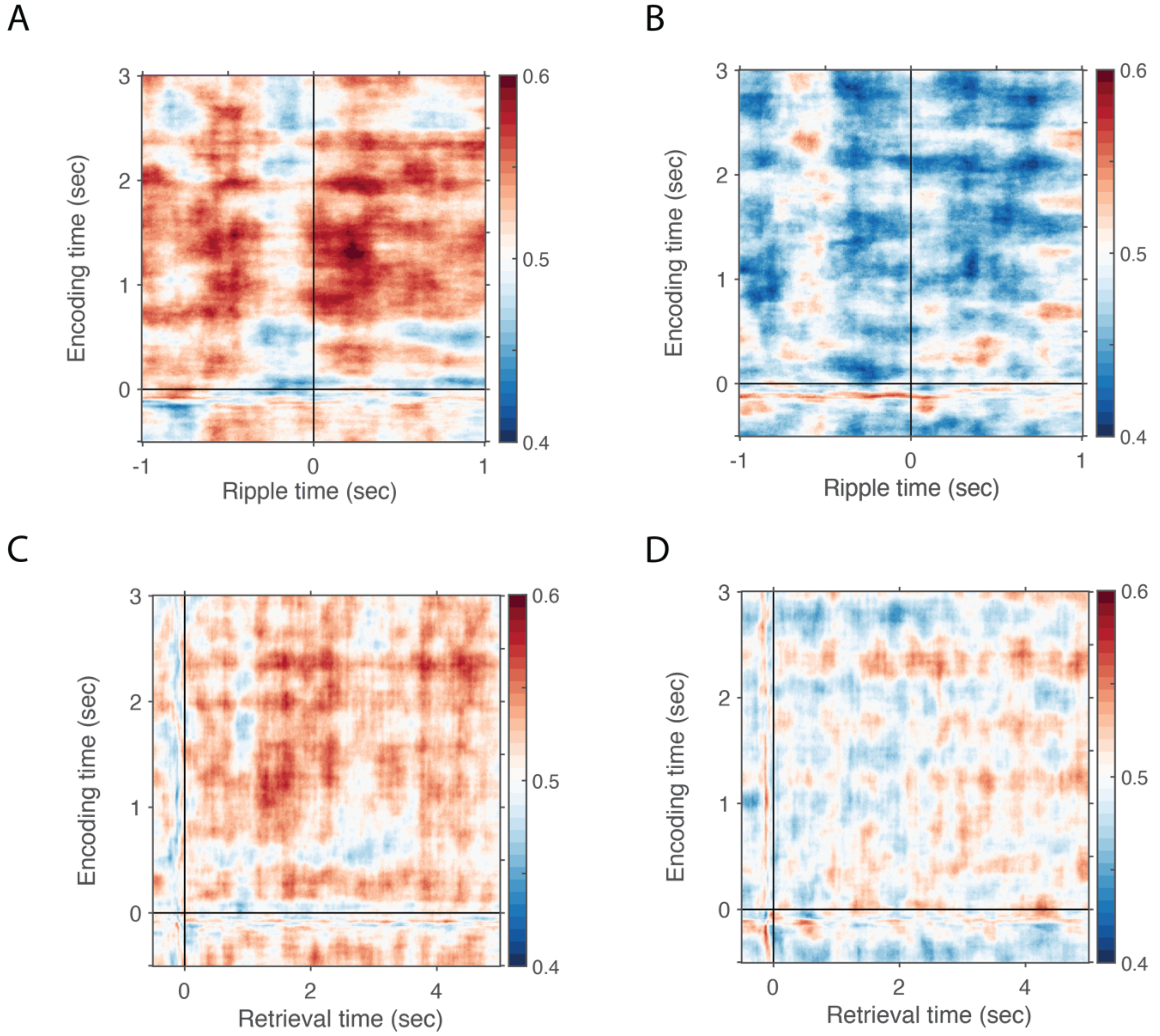

Reinstatement ripple-aligned and cue-aligned for AM+ and AM-, separately. **(A)** AM+ trials showed highest decoding accuracy following ripple events. **(B)** Chance-level decoding around ripple events for AM- trials. **(C)** When aligning the data to cue-onset at retrieval, we found the highest decoding accuracy around 1.5 to 2.5 seconds during encoding and retrieval. **(D)** Chance-level decoding during the entire retrieval period for AM- trials.

3) The claim that dimensionality increases after ripples is overstated; their statistics merely indicate a difference between conditions, not its exact timing. Concluding that dimensionality changed only after ripples and not before is incorrect. Fig S3A even shows a pre-ripple difference that goes unmentioned.

We appreciate the reviewer's comment and agree that our original wording may have suggested a stronger temporal claim than our statistics strictly justify.

First, our primary conclusion about timing is based on time-resolved, cluster-based permutation tests on the dimensionality time course, aligned to ripple onset. These tests

were conducted across the full -1 to +1 s window and revealed significant clusters only after ripple onset (470-840 ms post-ripple, p < .05, cluster-corrected), with no clusters reaching significance in the pre-ripple interval. Thus, the statement that dimensionality "increases following ripples" is grounded in time-resolved statistics rather than a single time-collapsed contrast.

Second, we explicitly tested pre- vs post-ripple epochs within each condition. For AM+ trials, dimensionality was significantly higher after ripples than before (before: M = 2.43, SE = 0.05; after: M = 2.50, SE = 0.06; t(11) = 2.22, p = .049), whereas AM- trials showed no such change (before: M = 2.44, SE = 0.06; after: M = 2.41, SE = 0.06; t(11) = −0.45, p = .66). A linear mixed-effects model with factors condition (AM+/AM-) and time (pre/post) also revealed a significant interaction, indicating that ripples are associated with a selective increase in dimensionality for remembered events. Together, these analyses go beyond a simple AM+ vs AM- difference collapsed across time and directly address the temporal relationship to ripple onset.

We agree, however, that we cannot prove that dimensionality does not change before ripples, only that we did not find reliable pre-ripple differences that survive correction for multiple comparisons using the 60ms sliding window in the main analysis.

With the additional analysis in "major concerns, C", we now have more evidence of AM+ trials showing increased dimensionality after ripple events, however, still with the caveat of not interpreting cluster-based permutation in the temporal domain.

To address the reviewer's concern, we have softened and clarified our wording in the main text. Instead of stating that dimensionality changed "only after ripples", we now write that dimensionality was selectively higher in AM+ than AM- trials and that this difference was most pronounced in the post-ripple window, where time-resolved cluster-based statistics and pre/post contrasts both show reliable effects, whereas we did not observe significant differences in the pre-ripple period (using the 60ms sliding window). We believe this revised phrasing more accurately reflects the temporal resolution and inferential limits of our analyses.

4) The PAC analyses are overinterpreted; PAC does not demonstrate inter-areal communication (see Schneider et al, 2021, Neuron, for a related discussion).
(Gamma) Oscillations: no justification provided that this is an actual oscillation. Frequency bands are defined arbitrarily rather than empirically. If anything, the plot in 6A shows that this is broadband, rather than narrow band activity. The latter would, however, be a prerequisite for an oscillation.
The choice of time window (-4 to 6 s) for PAC is also puzzling; extending by 1 s each side should have sufficed. Details on whether PAC analyses were limited to retrieval are missing. Another question that comes to mind here is why the authors did not look at coupled ripples in the cortical electrodes (see Vaz et al.) and how this might relate to any of their theta gamma findings? This could strengthened their point about neuronal communication.
Fig 6B shows that close to the peak power (for both gamma and theta), there is higher PAC. Seems trivial, given the authors chose the data in a way that they would have the highest signal to noise ratio at this particular point.

Why are there no memory contrasts for PAC, as for LDA and PCA analyses?
Fig 3A: It seems there are 9 data points for gamma, and 3 for theta; why?

We thank the reviewer for these thoughtful comments and are grateful for the opportunity to clarify the purpose and interpretation of the PAC analysis.

First, we fully agree that phase-amplitude coupling should not be taken as direct evidence of inter-areal communication. As Schneider et al. (2021) emphasise, LFP-based coupling measures (including coherence and PAC) primarily reflect correlations induced by afferent synaptic inputs and oscillatory power and, on their own, do not reveal effective connectivity or transmission gain. In line with this view, we have revised the text to avoid causal language and no longer interpret TG-PAC as a mechanism for communication. Instead, we describe it as a statistical marker of coordinated activity between hippocampal theta phase and cortical gamma amplitude that may co-occur with hippocampal-cortical interactions, without claiming that PAC itself implements communication. The paragprah reads as following:

Despite theoretical accounts emphasising its importance, there is currently little direct evidence in humans that theta-gamma-phase-amplitude-coupling (TG-PAC) supports statistical dependencies between regions during episodic memory retrieval. One likely reason for this gap is the considerable variability in retrieval timing across trials, which poses a challenge for aligning neural events consistently. Our findings suggest that hippocampal ripples can be leveraged to pinpoint the exact moments of reinstatement. Thus, we hypothesised that any statistical marker of coordinated activity between hippocampus and cortex would occur shortly after ripple onset, but prior to the cortical expansion of memory representations, and would be critical for reconstructing the memory trace.

Regarding the oscillatory nature of the gamma signal: the reviewer is correct that gamma bands should ideally reflect narrow-band activity rather than broadband increases. This is precisely why we implemented a two-step procedure: (i) removal of the 1/f background using FOOOF to ensure that peak frequencies were identified empirically rather than selected arbitrarily, and (ii) PAC computation restricted to ±10 Hz around each participant's individual gamma peak and ±2 Hz around their individual theta peak. This ensured that PAC was evaluated on narrow-band activity centred on genuine oscillatory peaks (Fig. 4A). The PAC analysis itself was not performed on the FOOOF-isolated oscillatory component.

The reviewer also asks why we analysed a -4 to 6 s window. This broader window was used from stimulus onset for peak detection only, not for PAC estimation. We wished to include sufficient cycles, especially at low frequencies: following Cohen's recommendation (Analyzing Neural Time Series Data), a minimum of three full cycles is required for a reliable estimate, which for 1 Hz implies at least 3 s of data. Our 10 s window thus provided ~10 cycles at 1 Hz, offering a robust basis for identifying spectral peaks. Once the individual peaks were identified, PAC was computed exclusively in the -1 to 1 s ripple-aligned window, consistent with all other ripple-based analyses. We now state this explicitly in the Methods.

We also agree that we might not have been clear enough on the phase which we analysed for the PAC analyses. We now state explicitly that PAC was computed only during retrieval and only on ripple-containing retrieval trials and reads as following:

This allowed us to assess TG-PAC in narrow-band activity centred on peak frequencies, time-locked to ripple events during retrieval (-1 to +1 s).

The reviewer raises an important question regarding cortical ripples and their potential relation to TG-PAC. In the present study, our primary focus was on hippocampal dynamics that precede cortical representational changes, and we therefore did not systematically analyse cortical ripples. We now briefly note this in the Discussion section to acknowledge the link to Vaz et al, which reads as following:

While some studies have linked slow vs. fast gamma to encoding and retrieval respectively [46,80,81], we found no consistent frequency separation across participants. This may reflect region-specific gamma profiles outside the medial temporal lobe. Importantly, we also acknowledge that our cortical gamma-band likely includes contributions from high-frequency events in the ripple range; rather than assuming strict separation between gamma and cortical ripples, we interpret our findings as evidence that hippocampal theta rhythm gates a broad class of high-frequency cortical activity, including potential ripples, that precedes and predicts ripple-locked increases in cortical dimensionality [35].

Regarding Fig. 4B, the reviewer notes that PAC appears strongest near the peak theta and gamma frequencies and suggests this may be trivial given higher SNR. Because PAC was computed relative to a trial-shuffled surrogate baseline (which we have now increased from 250 to 500 shuffles per participant), any effects driven purely by local power or waveform shape are captured in the surrogate distribution. The significant TG-PAC therefore reflects a consistent cross-trial alignment between hippocampal theta phase and cortical gamma amplitude that exceeds what would be expected from power differences alone. We clarify this point in the text.

The reviewer also asks why PAC analyses do not include memory contrasts. We have now added AM+ versus AM- comparisons to the revised manuscript and it reads as following:

We observed a significant coupling between hippocampal theta phase and cortical gamma amplitude around the identified peaks when contrasting AM+ with the shuffled control ($p_{cluster}$<.05; Fig. 4B). We did not find a significant difference between AM+ and AM- trials, suggesting that TG-PAC is as a general feature of retrieval-related hippocampal-cortical coordination rather than a memory-specific effect.

Finally, the apparent discrepancy in Fig. 4A (nine gamma points and three theta points) is purely a plotting artefact: several participants share identical peak frequencies, so multiple individuals collapse onto the same marker. As in all other analyses, the PAC analysis is based on all 12 participants. We have now ammended Figure 4A, with a slight offset between individual points.

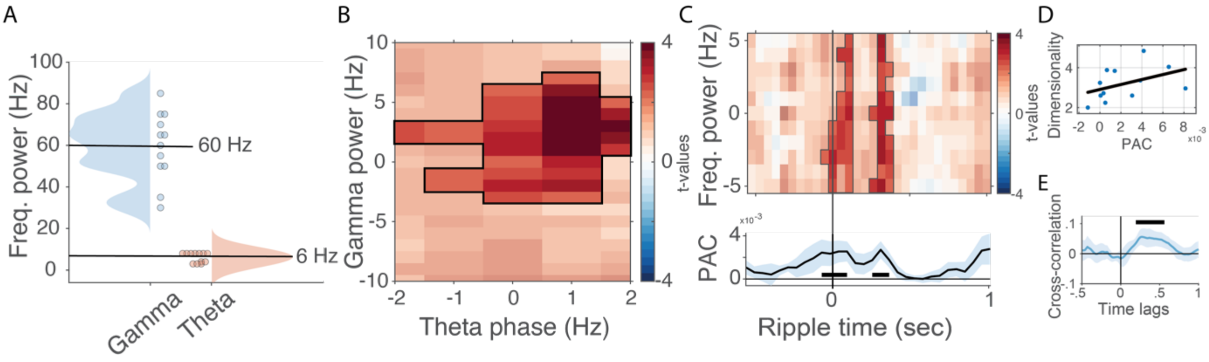

Figure 4. Phase-amplitude coupling following ripple events are related to dimensionality expansion.
(A) On average gamma oscillations peaked at 60 Hz, whereas theta on average peaked at 6 Hz. **(B)** Significant TG-PAC around peak frequencies for respective frequency (0 on x and y axes). **(C)**. Top: A time-resolved TG-PAC commencing around ripple events and persisted for approximately 400ms after the events. Bottom: Plotting the time-resolved TG-PAC averaged across the y-axis in top panel. **(D)** A positive correlation between TG-PAC and cortical dimensionality expansion, such that stronger TG-PAC was related increased dimensionality expansion. **(E)** A cross-correlation showed that TG-PAC leads dimensionality expansion with a peak at approximately 260ms.

5) Do the main results hold when AM- items are split into wrong vs. no memory and then separately compared to AM+ items?

We thank the reviewer for raising this important concern regarding the heterogeneity of the AM- condition, which contains both incorrect responses and "don't know" responses. To address the reviewer's concern, we conducted additional analyses treating incorrect and don't know trials as separate categories.

Dimensionality analyses.
Both AM+ vs. incorrect and AM+ vs. don't know comparisons showed significant increases in post-ripple cortical dimensionality (same window as Fig. 3B). That is, successful retrieval reliably engaged higher-dimensional cortical states regardless of whether the comparison class was uncertainty-driven or error-driven.

Decoding analyses.
When comparing AM+ vs. don't know, we observed a significant decoding difference in the same post-ripple time window reported in Fig. 3A. When comparing AM+ vs. incorrect, we found a trend-level cluster (p = .09) in the very same time window. The temporal profile of both contrasts closely matched the original AM+ vs. AM- effect, indicating that the reinstatement differences are not specific to the "don't know" category.

Together, these results demonstrate that our main findings of enhanced reinstatement and increased dimensionality hold when don't know and incorrect trials are analysed separately and are now included in the revised manuscript Supplementary Fig. 4.

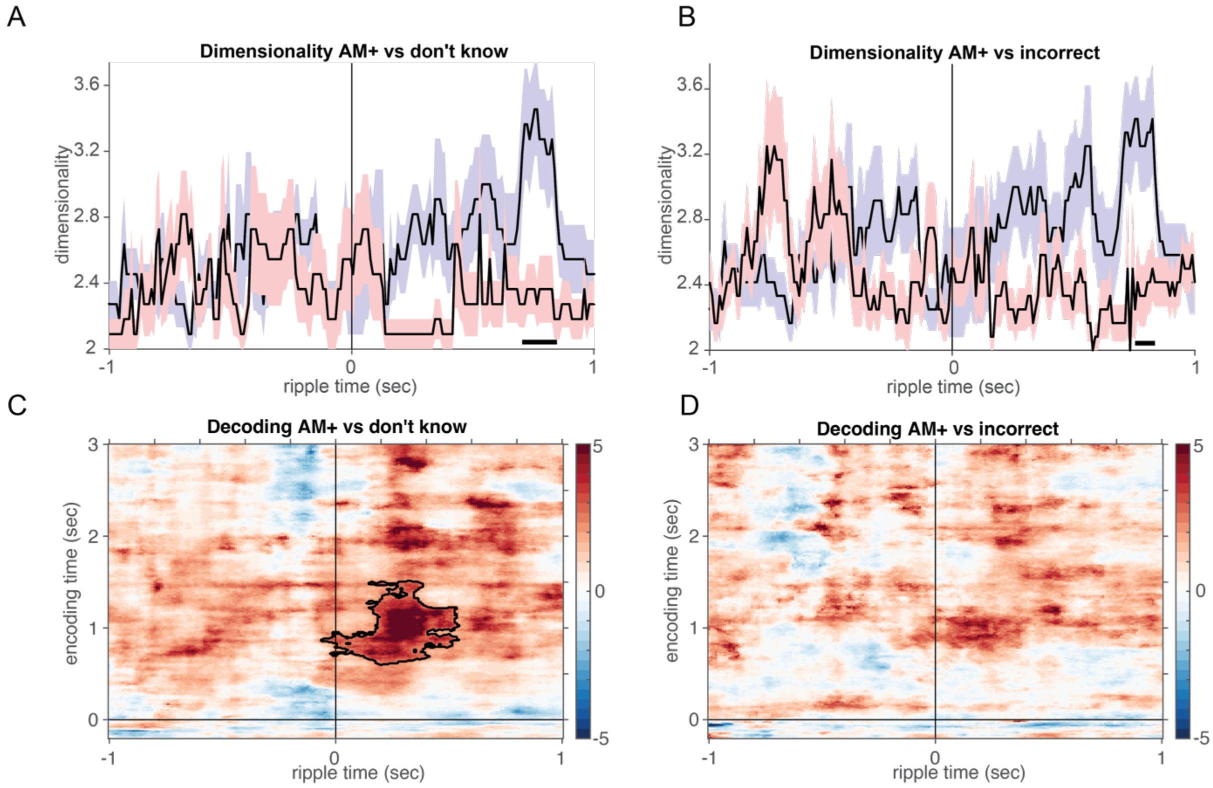

Dimensionality and reinstatement analyses dividing AM- into incorrect and don't know responses. **(A)** AM+ trials showed significant higher dimensionality as compared to don't know responses in a similar time window as main analysis in Fig. 3B. **(B)** AM+ trials showed significant higher dimensionality as compared to incorrect responses in a similar time window as main analysis in Fig. 3B. **(C)** AM+ trials showed significant higher decoding accuracy as compared to don't know responses in a similar time window as main analysis in Fig. 3A. **(D)** AM+ trials showed numerically higher decoding accuracy as compared to incorrect responses in a similar time window as main analysis in Fig. 3A.

6) Using a common average reference across all electrodes eans that all electrode will share some variance, also hippocampal and cortical electrodes. Separate references for each would be more appropriate, given the framing of these as distinct groups.

We thank the reviewer for raising this important methodological point regarding the referencing scheme. To address the concern that a common average reference might introduce shared variance between hippocampal and cortical electrodes, we reran all primary analyses using separate average references for hippocampal and cortical channels.

Across analyses, the results remained highly similar (and even stronger in some cases) to those obtained with the original common average reference. Ripple density ($t(11) = 5.49$, $p < .01$), ripple-triggered increase in cortical reinstatement ($p_{cluster} < .05$), and the linear mixed effects model (significant interaction indicating greater ripple-induced expansion for remembered events (Estimate = -.10, SE = .02, $t(20,380) = -4.66$, $p < .001$) were all highly similar to the original analyses. The only analysis not showing a significant difference between AM+ and AM- was the time-resolved dimensionality measure (See figure below), which is not unexpected, as dimensionality estimates are intrinsically sensitive to the covariance structure of the data. Referencing schemes directly affect cross-channel

covariance by construction, and separating hippocampal and cortical references necessarily alters the global variance structure from which dimensionality is derived. As a result, this manipulation reduces sensitivity for detecting subtle differences in representational geometry over time.

Because the core findings are preserved under both referencing schemes, and because changing the entire pipeline would require substantial restructuring without altering the interpretation, we retain the common average reference as the main approach in the manuscript.

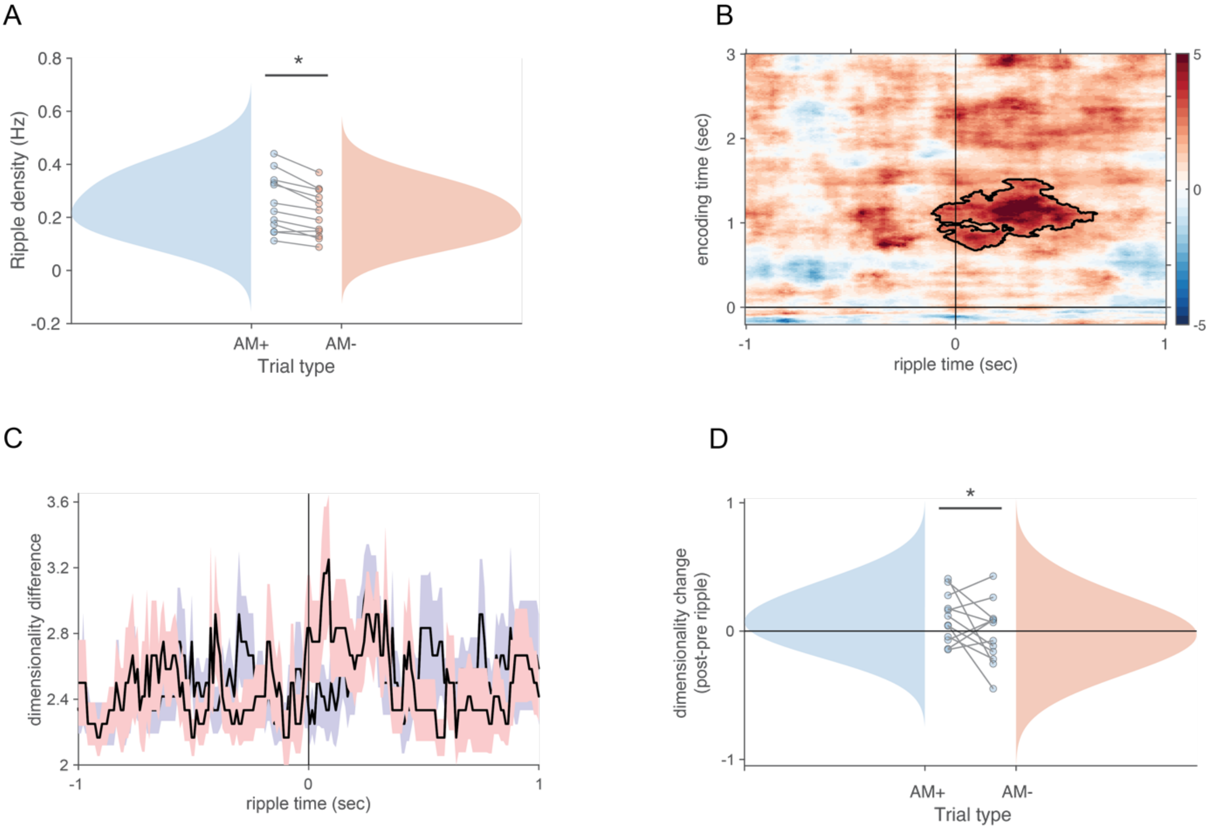

Main analyses using common trimmed average for hippocampal and cortical contacts, separately. (A) Ripple density was significantly higher for AM+ vs. AM- trials. (B) Reinstatement analysis showed similar cluster as in main Figure 3A. (C) Time-resolved dimensionality analysis did not show the same significant cluster around 400-800ms post ripple. (D) The linear mixed effects model showed the same significant interaction, indicating an increase in dimensionality for AM+ vs. AM- trials.

7) Statements such as “hippocampal ripples initiate a process…” are unwarranted; temporal precedence alone does not imply causality. Please check carefully whenever causality is implied.

We thank the reviewer for this important point. We fully agree that our findings are correlational and do not permit strong causal claims. In response, we have carefully revised the manuscript to adjust any phrasing that may have implied causality. Specifically, we have replaced terms such as “underlies” and “initiate” with more appropriate language (e.g., “is associated with” or “follows”), to better reflect the observational nature of our data.

We appreciate the reviewer’s attention to this detail, and we are confident that the revised wording ensures more accurate and scientifically responsible interpretation of our results.

8) Figure scales, colour schemes are sometimes confusing and may mislead readers.
-The scale in Fig S2B is decoding accuracy, while the scale in Fig 3A is t values, making it impossible to compare them. The authors need to provide a version of Fig 3A using the same scale as in Fig S1A and B.

We thank the reviewer for this attention to details, and have now added Supplementary Figure 3 to show decoding accuracy for AM+ and AM- trials, both ripple-aligned and cue-aligned.

-The use of the color scheme in FS2B is misleading. While in many other plots white indicates zero values, white indicates intermediate values. This gives the impression as if decoding accuracy would be very low within the boundaries of the outline when it is not. Overall, this way of presenting the data may leave readers less confident in the results.

Again, we thank the reviewer for this attention to details, and have made sure that all the figures are easier to interpret.

9) Questions on the dimensionality analysis:
-The choice / number of components for the dimensionality analysis seems arbitrary (page 8). Why these four and not others or more or less components?

We thank the reviewer for raising this important point. Our choice of components for the dPCA analysis was not arbitrary but based on a principled, data-driven procedure tied directly to 1) variance explained and 2) the presence of significant decoding around ripple onset.

Across participants, dPCA returned multiple components assigned to each marginalisation (target association, memory, interaction, and condition-independent). Among these, five components showed significant decoding aligned to ripple events in the same temporal window where we observed the PCA-based dimensionality increase (dPCs 1, 2, 3, 4, and 13). To avoid redundancy and ensure comparability across marginalisations, we selected one component per marginalisation. Specifically, the component that explained the largest amount of variance within that marginalisation. This yielded dPC1 (interaction), dPC2 (target association), and dPC4 (memory). We initially excluded dPC3 because it belonged to the same marginalisation as dPC2 (target association) and explained less variance.

Thus, the selected components (dPCs 1, 2, and 4) were chosen because they were the highest-variance representatives of each task variable, they showed significant ripple-locked decoding, and they provided full coverage of all experimental factors without redundancy.

As shown in Supplemental Fig. 9, the full set of 50 components was retained in all reconstruction steps; our selection only concerns which axes were used for visualisation and clustering. Additionally, we confirmed that the main effects are robust to using the alternative component set (dPCs 1, 3, and 4). The same ripple-locked increases in separability and label-defined silhouette scores appear for both sets, demonstrating that our conclusions are not dependent on a particular choice of components.

-Fig 3 shows elevated dimensionality is prior to ripple onset. Why would that be?
-The cluster based statistics tests for differences between groups/conditions, but the temporal domain cannot be interpreted in the way the authors do it here.
-The authors should repeat their main dimensionality using the shuffled data in FS2A and B, separately.

We agree with the reviewer that we cannot interpret the temporal domain in the way we did in the previous manuscript. We performed an additional analysis to understand whether the ripples are privileged when it comes to reinstatement and dimensionality expansion.

An ideal control analysis would assess whether ripple onset provides a uniquely effective alignment point relative to all other moments in the retrieval epoch. Conceptually, this is precisely the question that CircCorrect addresses: each circular shift effectively reassigns ripple onset to a different temporal offset within a participant's pool of ripple onsets, thereby sampling the entire retrieval time-series as potential alignment points while preserving temporal autocorrelation.

To address the reviewer's suggestion directly, we have now implemented an additional control analysis in which we computed reinstatement to 60 hypothethical ripple events, from 1 to 5 seconds after cue onset at retrieval (see Figure below, panel A). We epoched the data at each time point, allowing 1 second before and after, exactly the way we did it in the main analysis. We then subtracted the mean of these trials and subtracted by the standard deviation and ran a cluster-based permutation analysis on this z-value. This provides an explicit test of whether ripple onset is statistically privileged. We now report this analysis in the revised manuscript (Supplementary Fig. 5), and it yields a significant cluster in the same time window as AM+ vs AM- reported in 3A. We thank the reviewer for this insightful comment, which has strengthen our case.

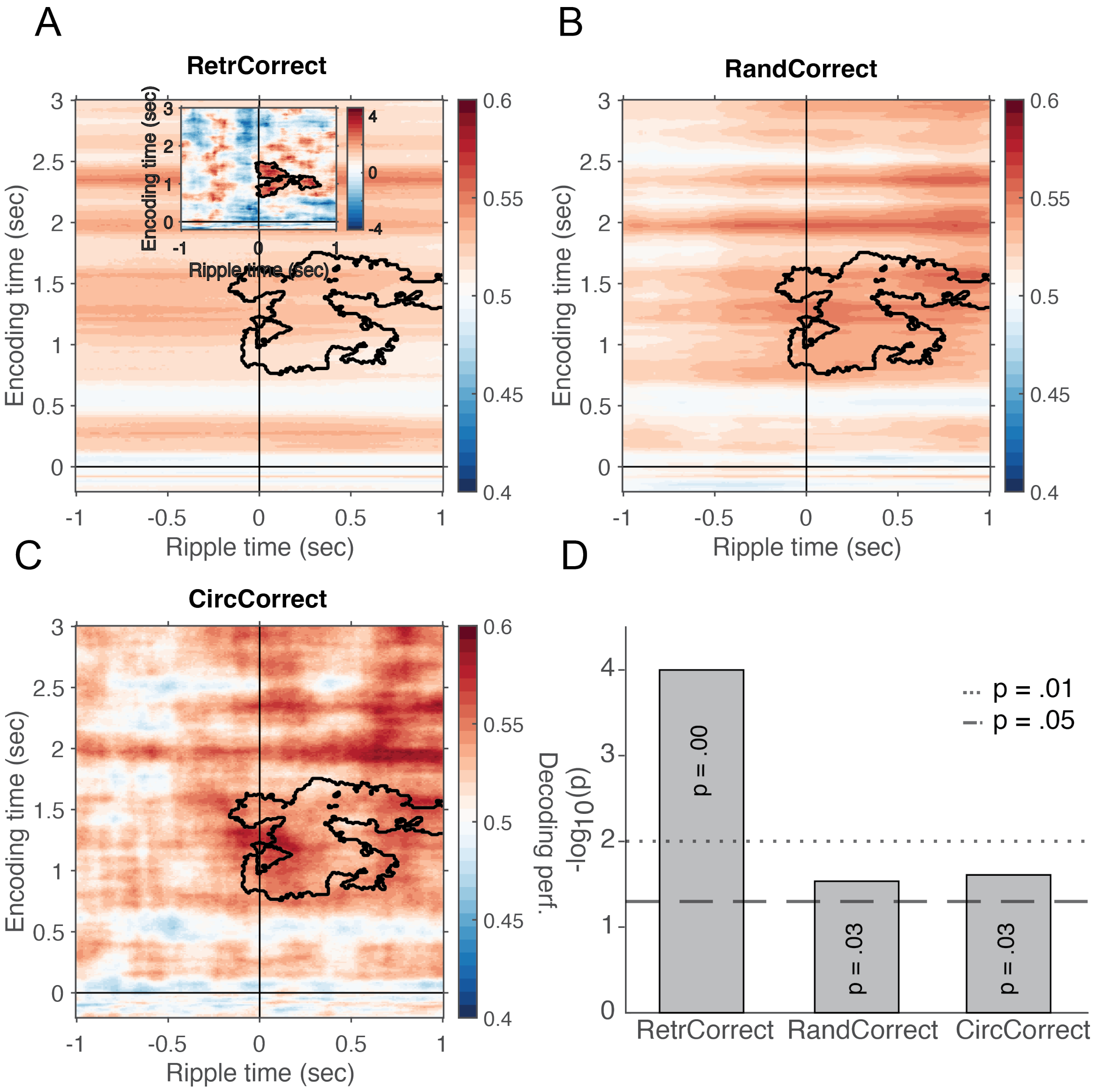

***Control analyses confirming ripple-triggered reinstatement specificity*. (A)** From 1-5 seconds after cue onset, we picked 60 time points and treated them as ripple events and performed the same analysis as in 3A. The time generalisation matrix shows the decoding accuracy for these events with the significant cluster identified in Figure 3A highlighted in black. Inset shows a cluster-based permutation between the permuted baseline and empirical data, with a significant cluster in the same time window as in Figure 3A. **(B)** Decoding accuracy when AM+ trials were shuffled 1000 times. **(C)** A similar analysis was performed using a stricter control in which ripple times were swapped with those of adjacent trials (i.e., neighbouring trial shuffling). This revealed significantly higher decoding in the empirical AM+ condition compared to the adjacent-trial control. **(D)** P-values when comparing empirical data with the different controls.

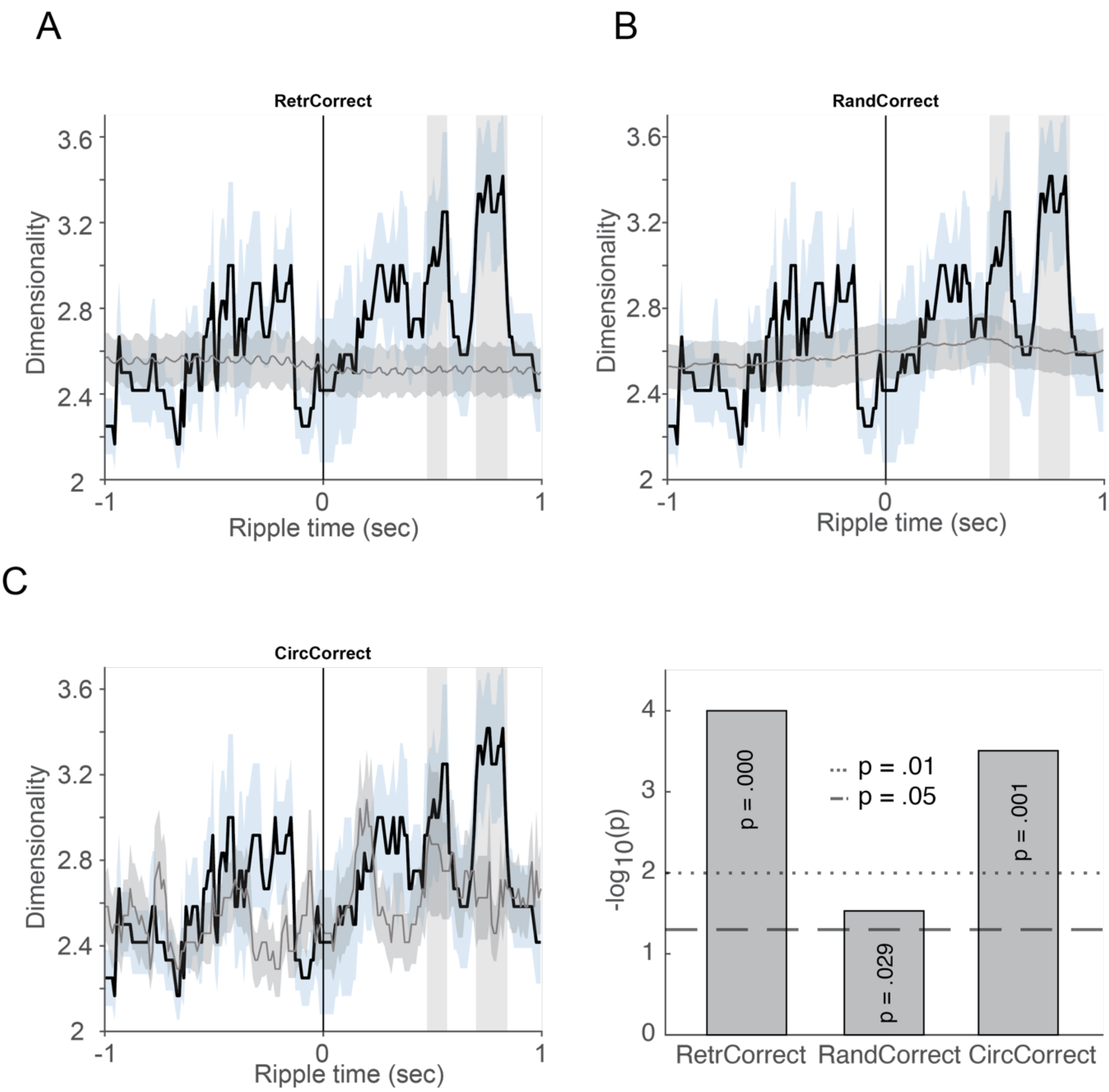

***Control analyses confirming specificity of dimensionality expansion* (A)** From 1-5 seconds after cue onset, we picked 60 time points for each participant and treated them as ripple events and performed the same analysis as in 3B. Grey is permuted baseline, blue is empirical data. **(B)** Shuffling AM+ trials 1000 times. Grey is permuted baseline; blue is empirical data. **(C)** Empirical AM+ (blue) and adjacent trials (i.e. temporally shifted controls, grey). **(D)** P-values when comparing empirical data with the different controls.

Minor
-Key papers are neglected:
Axmacher et al 2008 Brain, Dickey et al 2022 PNAS, Sakon& Kahana 2022 PNAS,
Kunz et al 2024 Nat Neurosci; and others
These and other related papers should be discussed in the light of the present findings.

We thank the reviewer for pointing us to these papers and apologise for the oversight. These references are now included in the revised manuscript.

- The authors should compare ripple density between unsuccessful vs. successful recognized items.

As requested, we compared ripple density between successfully recognised items and unrecognised items (i.e., misses). Recognition success was defined based on the old/new judgment, independent of associative recall (collapsing AM+ and AM- trials). Ripple density did not significantly differ between recognised and unrecognised trials (t(11) = 2.08, p = .061). This aligns with our primary finding that ripples are selectively increased for successful associative retrieval (AM+), rather than for recognition success per se.

-50% correct associative retrieval may indicate guessing behavior, given two alternatives. How do the authors control for this in their data and how would that affect their EEG analyses?

We thank the reviewer for raising the important issue of potential guessing in the associative memory component. While the overall proportion of AM+ trials among hits is indeed close to 50%, this does not reflect 50% correct performance on a two-alternative forced-choice task. Crucially, participants were not forced to choose between the two associations on every "old" trial: they could explicitly indicate that they recognised the word but did not remember the associated colour/scene by pressing "don't know" ("?"). As a result, AM- trials are dominated by "don't know" responses (33.77% of hits), whereas truly incorrect associative choices constitute only 16.28% of hits.

To directly quantify guessing, we computed associative accuracy conditional on trials where participants actually chose one of the two associations (i.e., excluding "don't know" responses). On these choice trials, associative accuracy was markedly above the 50% guessing level (M = 76%, SE = 2%, range = 64%-86%; paired-samples t-test against chance: t(11) = 12.76, p > .01), demonstrating that participants did not simply guess between the two options when they committed to an associative decision. We thank the reviewer for pointing out this important clarification and have included the results on P. 4, l. 6-10, and reads as following:

On trials where they either picked the correct or incorrect association (AM+ and incorrect), associative accuracy was markedly above the 50% guessing level (M = 76%, SE = 2%, range = 64% - 86%; paired-samples t-test against chance: t(11) = 12.76, p > .01), demonstrating that participants did not simply guess between the two options when they committed to an associative decision.

- the authors interpretation that the supplementary analyses "confirms" that "reinstatement was specific to the ripple latency of the current trial" is not correct. The non-significant difference in Fig S2B confirms - if anything - that there is no (significant) difference, but offers no insight on the timing.

We thank the reviewer for pointing out that the non-significant contrast in Supplementary Fig. 2B cannot be taken as evidence that reinstatement is specific to ripple timing. We fully agree that a non-significant difference does not provide positive evidence about temporal specificity.

In the original manuscript, Supplementary Fig. 2 was intended only to demonstrate that the AM+ / AM- reinstatement difference is not an artefact of global reinstatement effect

occurring throughout retrieval. However, as the reviewer correctly notes, this does not determine whether ripple onset is the uniquely informative alignment point.

In response to this comment, we performed a new analysis designed to directly test temporal specificity. We computed reinstatement time-locked not only to the true ripple peaks but also to 60 pseudo-event onsets spanning 1-5 s after cue onset. Each pseudo-event provided an alternative alignment point, and the data were epoched (±1 s) and analysed identically to the real ripple events. This approach explicitly tests whether ripple onset is statistically privileged relative to all other moments in the retrieval period.

We found that the empirical data was significantly higher than chance in the same time window as the AM+ AM- contrast. This demonstrates that reinstatement is indeed temporally specific to the moment of ripple occurrence, and not merely a general property of retrieval-related activity. We have now added this analysis to the revised manuscript (Supplementary Fig. 5A and also included below).

In addition to this, we have also updated how we statistically assessed significance between empirical and permuted data, following a validated method by Stelzer et al (2013). Empirical values were first averaged within the time-frequency cluster identified in the main analysis, yielding a single cluster-mean value per participant. A group-level null distribution was then constructed by repeatedly sampling one surrogate estimate per participant from the corresponding control distribution and averaging these values across participants over 10 000 iterations. The empirical group-mean cluster value was compared against this null distribution to obtain a one-sided p-value. For the first control (RetrCorrect), empirical values were significantly greater than the group-level null distribution ($z = 6.05$, $p < .01$). For the second control (RandCorrect), empirical values again exceeded the null distribution ($z = 2.19$, $p = .029$). As no comparable group-level surrogate distribution was available for the CircCorrect control, empirical values within the significant cluster were compared directly with the circularly varied data using a paired-samples t-test. This analysis showed significantly stronger reinstatement for the empirical data relative to CircCorrect ($t(11) = 2.05$, $p = .03$).

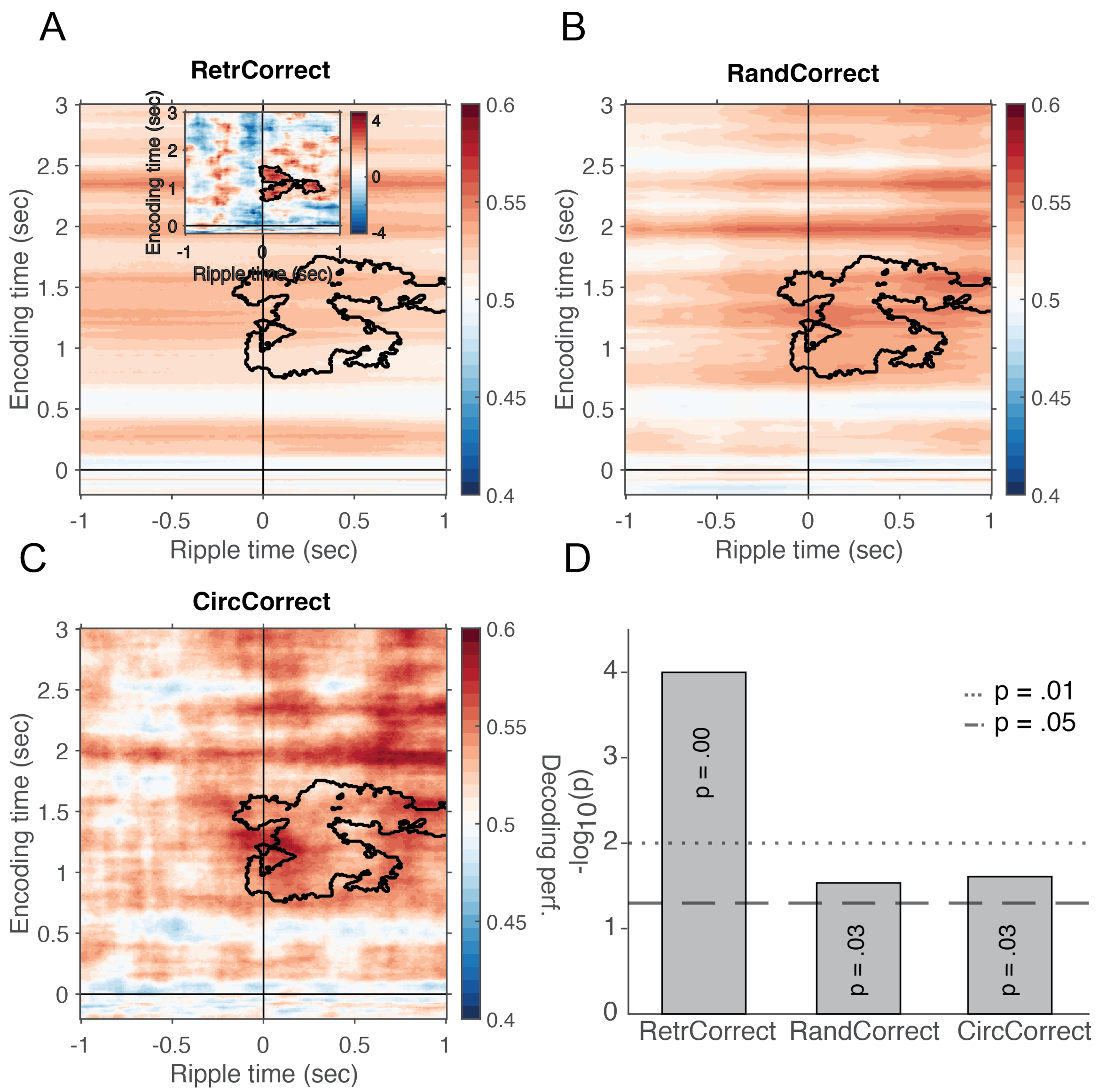

***Control analyses confirming ripple-triggered reinstatement specificity*.** **(A)** From 1-5 seconds after cue onset, we picked 60 time points and treated them as ripple events and performed the same analysis as in 3A. The time generalisation matrix shows the decoding accuracy for these events with the significant cluster identified in Figure 3A highlighted in black. Inset shows a cluster-based permutation between the permuted baseline and empirical data, with a significant cluster in the same time window as in Figure 3A. **(B)** Decoding accuracy when AM+ trials were shuffled 1000 times. **(C)** A similar analysis was performed using a stricter control in which ripple times were swapped with those of adjacent trials (i.e., neighbouring trial shuffling). This revealed significantly higher decoding in the empirical AM+ condition compared to the adjacent-trial control. **(D)** P-values when comparing empirical data with the different controls.

- Why employ a linear mixed-effects model for pre-post ripple comparisons, when previous contrasts relied on simple tests?

We thank the reviewer for this question. The reason we used a linear mixed-effects model for the pre-post comparison is that, for this analysis, the data structure is more complex than in the earlier contrasts that were tested with simple tests.

In the dimensionality time-course analysis, each participant contributes multiple blocks (3-6 per participant), and within each block we obtain repeated measures of dimensionality for each memory condition (AM+/AM-). This leads to a hierarchical, unbalanced dataset where observations are nested within blocks, blocks are nested within participants, and the number of blocks varies across participants.

Under these circumstances, simple tests on block-averaged values would either (i) ignore this nesting and treat non-independent observations as independent, or (ii) require collapsing across blocks, throwing away within-participant variability and reducing statistical power.

A linear mixed-effects model is therefore appropriate because it 1) accounts for non-independence of observations within the same participant and block (via random intercepts for participant and block); 2) handles the unequal number of blocks per participant without requiring ad-hoc averaging or listwise deletion; and 3) allows us to test interactions between condition (AM+/AM-) and time (pre/post) while correctly modelling the repeated-measures structure.

-Why is the amplitude in Fig 1a increasing before ripple onset?

We thank the reviewer for this comment. The gradual increase in voltage before the ripple peak likely reflects variability in the recording location within the hippocampus, as our clinical electrodes can sample different layers and depths along the longitudinal axis. Because sharp waves and ripples have different spatial profiles across CA1, averaging across channels could produce a smooth pre-ripple deflection. This pattern is commonly observed in human data (e.g., Kunz et al., 2024) and probably does not reflect anticipatory neural activity.

-Figure S1 shows that bilateral hippocampus electrodes for patient 12, but the authors state that only one hemisphere would be included in the analyse. In case this is not an error in the figure, the authors need to repeat all group level analyses.

We thank the reviewer for this comment and want to highlight that we in the Method section P.19, l. 26-27, stated "for one participant, both hemispheres were considered healthy"

-Page 5 Line 29: the authors do not show replay

We thank the reviewer for this comment. To avoid any potential confusion, we have revised the manuscript to use the term reinstatement throughout and removed any wording that could be interpreted as implying sequential replay.

-Selective interpretation of how the authors' own findings align:
Authors don't say that cortical reactivation was immediately following ripples, without delay, which is not in line with Michelmann et al. A less opaque discussion would be more interesting.

We thank the reviewer for this helpful comment and agree that the Discussion should more explicitly clarify how our temporal findings relate to prior work such as Michelmann et al. In

that study, analyses were anchored to cortical reinstatement events in sensory regions, and hippocampal activity was shown to precede these cortical reinstatement events by several hundred milliseconds, reflecting a consistent hippocampal–cortical lag during memory retrieval.

In contrast, our analyses are explicitly ripple-locked, aligning cortical activity to internally generated hippocampal output events themselves. When time-locked to hippocampal ripples, we observe a rapid onset of reinstatement immediately following the ripple, followed by a broader cortical dimensionality expansion that peaks approximately 400–800 ms later. These findings are fully compatible with prior results: anchoring analyses to cortical reinstatement emphasises the hippocampal lead relative to reinstatement, whereas anchoring analyses to hippocampal ripples isolates the initiating hippocampal event and reveals both the immediate triggering of reinstatement and its subsequent cortical propagation.

To make this distinction explicit, we have revised the Discussion to clarify that our results do not imply an absence of hippocampal–cortical delays, but rather reflect the use of different temporal reference points. Within this framework, the later phase of dimensionality expansion we observe aligns well with the timescale of cortical reinstatement reported previously, while the ripple-locked analysis captures the initiating hippocampal output that precedes it.

This is how it reads in the discussion now:

Recent studies have examined the temporal relationship between hippocampal activity and cortical reinstatement. Michelmann and colleagues [73,74] showed that when analyses are anchored to cortical reinstatement events in sensory regions, hippocampal activity reliably precedes cortical reinstatement by approximately 500–740 ms during memory retrieval. Importantly, these studies do not align reinstatement to internally generated hippocampal output events themselves. In contrast, our analysis is explicitly ripple-locked. When aligned to hippocampal ripple peaks, we observe reinstatement emerging immediately following the ripple, followed by a broader increase in cortical dimensionality that peaks approximately 400–800 ms later. Thus, our findings complement those of Michelmann et al. by showing that reinstatement begins rapidly following hippocampal ripples, while large-scale cortical reorganisation unfolds over several hundred milliseconds, consistent with multi-synaptic propagation across cortical networks [75,76].

-Include a reference from animal literature about replay compressions
Included a paragraph on replay compression in the Discussion P.14 l. 37 - P.15 l.4 and reads as following:

We identified hippocampal ripples as triggers of this cortical expansion. Ripple events were more frequent on successful trials and reliably preceded both reinstatement and increased dimensionality. This finding supports a novel account of ripple events: rather than merely reactivating previously stored patterns, ripples appear to initiate a transformation that reconstructs compressed memory representations into complex, high-dimensional cortical states. Such expansion may reflect the recruitment of a broader set of cortical areas and the unfolding of more differentiated neural activity patterns, enabling flexible decoding of specific mnemonic content. This interpretation is consistent with extensive rodent literature showing that hippocampal ripples carry time-compressed replay of recent experience [71–73]. Such

compressed ripple content provides an efficient substrate for broadcasting mnemonic information to cortex, where it can be expanded into high-dimensional representations that support detailed reconstruction.

-Figure 1a, legend: What about unsuccessful recognition?

Not completely sure what the reviewer is referring to.

-Figure S4B: plotting problem?

We thank the reviewer for this comment and want to clarify that this is not a plotting problem, but dimensionality plotted for hippocampal channels. Due to the low channel count, this estimate will inevitably be a very sluggish measure.

REVIEWER COMMENTS

Reviewer #1 (Remarks to the Author):

The authors have done a very rigorous job following up on my many questions. I generally believe the manuscript is now a detailed, valuable resource for readers despite some minor remaining concerns.

We thank the reviewer for their thoughtful comments and positive evaluation, which we greatly appreciate.

1. The authors addressed my comment by splitting their cortical contacts into anterior vs. posterior temporal cortex and re-running the primary analyses from Figure 3A-B. The authors seem to want to elide the fact that the posterior contacts might drive their significant observations, but I don't think this weakens the overall conclusions and the additional anatomical information is perhaps interesting to include and discuss for the reader as a supplementary figure. Particularly in relation to PMID 39970226 which is such a complementary work to this.

We thank the reviewer for this comment and agree that this is indeed a very interesting result, especially in the light of the reference the reviewer provided. In the revised manuscript, we have added a Supplementary Fig 5 and are also discussing this in Discussion:

… and this effect was stronger in posterior as compared to anterior channels (Supplementary Fig. 5d-e), in line with recent work on transformation of memory traces during episodic memory retrieval (Rau et al., 2025).

2. The authors do a great job of identifying putative interictal spikes and demonstrating that spike-aligned analyses do not replicate their ripple-aligned results. But I still think it is important to address a couple of remaining questions:

a. Why are the degrees of freedom for the t-test between AM+ and AM- spike counts only 11 (subject level) when there are 72 hippocampal contacts and hundreds of temporal contacts? Both regions are well known to show higher IED counts during poor memory encoding (hence my original concern).

We thank the reviewer for this thoughtful comment and the opportunity to clarify the degrees of freedom used in this analysis. Throughout the manuscript, all statistical inferences are performed at the subject level, rather than the channel level, to avoid inflating degrees of freedom due to non-independence of channels within participants. We therefore adopted the same approach for the spike-triggered analyses, resulting in 12 participants (df = 11). While we acknowledge that this approach reduces statistical power, we believe it provides a more conservative and robust estimate of effects at the group level. We do see numerically more spikes for AM- (M = 174, SE = 69) as compared to AM+ trials (M = 117, SE = 30), but no difference in number of spikes ($t(11) = -1.08$, $p = .30$) on a group level.

Regarding the reviewer's point that interictal epileptiform discharges (IEDs) are often elevated during unsuccessful encoding, we agree that this is an important finding. However, our study focuses on the retrieval phase, and we did not analyse IEDs during encoding. Within the retrieval period, we do observe a numerical, but not significance difference in spike counts between AM+ and AM- conditions.

Additionally, to further assess the influence on IEDs on our results, we repeated the main analyses after excluding data within 1 second of detected spike events. Importantly, this exclusion was applied across all channels, such that ripple events were removed irrespective of whether they occurred on the same channel as the detected spike. The results remained qualitatively unchanged, indicating that our findings are not driven by epileptiform transients (see below).

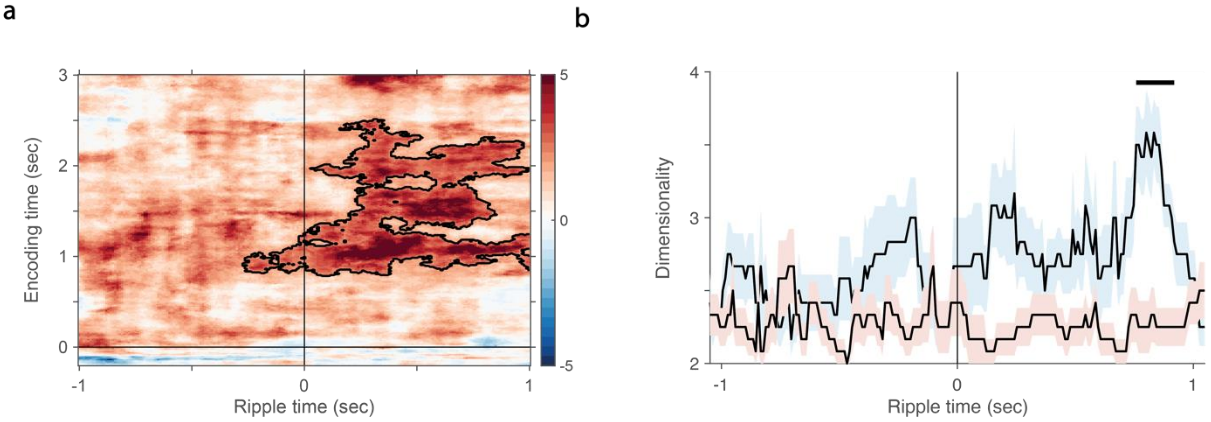

Spike-corrected reinstatement and dimensionality. ***a*** Reinstatement analysis excluding ripples occurring within ±500 ms of spikes in hippocampal channels revealed a similar significant cluster as in Fig. 3a. **b** The spike-corrected time-resolved dimensionality analysis showed a comparable significant cluster around 700–800 ms post-ripple, consistent with Fig. 3b.

b. Even if spike counts are the same across AM+ and AM-, it is odd to me that the dimensionality doesn't seem to change at all in both conditions then around the time of interictal spikes in the new supplementary plot. How do the authors interpret this? I would expect the variance to collapse to 1-2 dimensions around interictal activity to reflect the presumably synchronized activity across multiple electrodes.

We thank the reviewer for this insightful comment. Importantly, in our analysis pipeline, putative interictal spikes were detected in hippocampal channels, whereas dimensionality was estimated from non-hippocampal channels. As such, the expectation of a collapse in dimensionality implicitly assumes that interictal spikes induce widespread, synchronous activity across this broader, anatomically distributed set of electrodes.

However, interictal spikes are typically spatially heterogeneous and locally expressed phenomena, and do not necessarily produce globally synchronous activity across regions outside the hippocampus (Keller et al., 2010, Brain). Consequently, even if local synchrony is present near the spike source, this would not be expected to manifest as a population-wide collapse in dimensionality in non-hippocampal recordings.

We analysed the spatial extent of detected interictal spikes by quantifying the number of hippocampal channels involved in each spike event. The majority of spikes were observed on one channel (M = 81%), with only a small proportion occurring simultaneously across two (M = 15%) or three (M = 3%) contacts (see figure below). This indicates that interictal activity in our dataset is predominantly spatially restricted rather than broadly distributed across electrodes. If we expanded the window to 50ms, we found that 69% of the spikes were isolated to one channel.

Taken together, these findings further argue against the notion that interictal spikes induce widespread, globally synchronous activity across the recorded population. Instead, they are consistent with the view that interictal events reflect locally expressed dynamics, which would not be expected to produce a collapse in dimensionality across anatomically distributed, non-hippocampal channels.

We therefore interpret the absence of a dimensionality reduction around interictal spikes as consistent with the spatially restricted nature of these events, rather than evidence against their presence or detection.

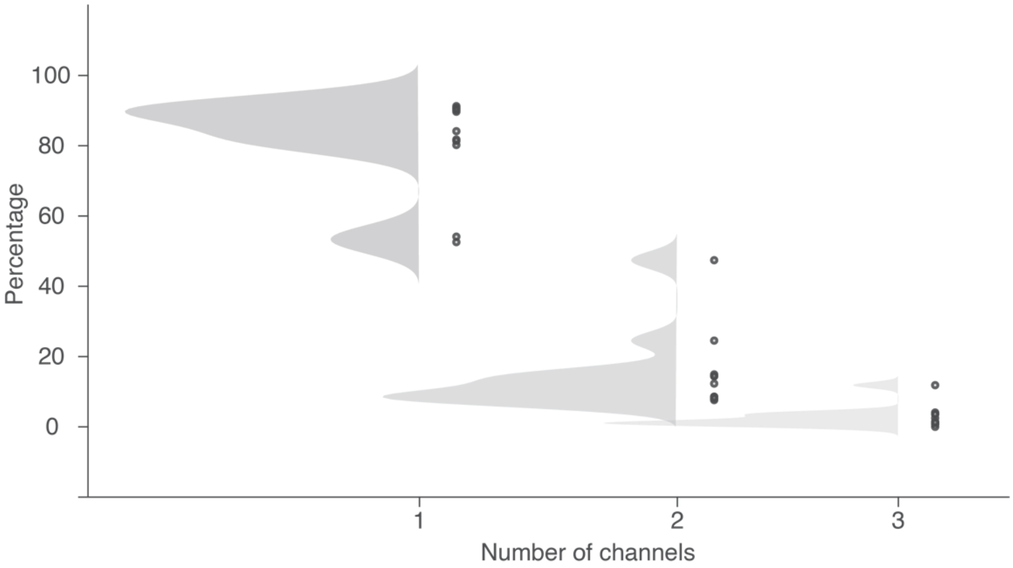

Spatial extent of interictal spikes across hippocampal channels. Distribution of the percentage of spike events involving one, two, or three channels. Each point represents a participant, and the violin plots illustrate the across-participant distribution. The majority of spikes were confined to a single channel, with progressively fewer events involving two or three channels, indicating that interictal activity was predominantly spatially local rather than broadly distributed across electrodes.

3. I appreciate the authors' answer re: bipolar referencing not making sense for such an analysis but I still don't have a firm answer for why a trimmed common-average reference is appropriate. It is one thing to remove outlier channels entirely because you suspect they are artifactual or noisy – that is fine. But that doesn't seem like the logic applied to this trimmed common average and it would be nice to see firmer justification or some assessment of the impact it has on the results.

We thank the reviewer for this insightful comment and for prompting us to clarify our rationale. Our use of a trimmed common-average reference was motivated by the desire to

reduce the influence of transient, high-amplitude noise or artifactual channels on the reference signal. In contrast to excluding channels entirely, trimming allows us to retain all channels while preventing a small number of extreme values from disproportionately biasing the common average at any given time point.

Importantly, our intention was not to selectively remove biologically meaningful signals, but rather to ensure that the reference was not dominated by outliers that are unlikely to reflect coherent neural activity.

To directly address the reviewer's concern, we repeated the key analyses using a standard (untrimmed) common-average reference. The results of both the decoding and dimensionality analyses were qualitatively unchanged, indicating that our findings are not dependent on the use of a trimmed reference. In particular, the decoding analysis provided a significant cluster ($p < .05$) in the same time window as in our Fig. 3a, and the critical Condition × Half interaction remained significant ($\beta = -.053$, SE = .019, $t(20380) = -2.72$, $p = .007$), indicating that the key effect is not dependent on the trimming procedure.

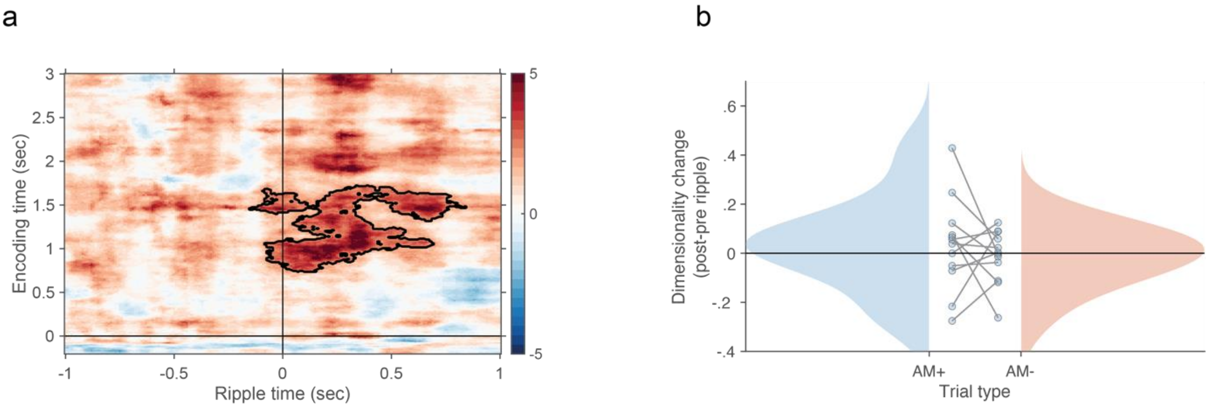

Decoding and dimensionality analyses using common average. (A) Similar significant cluster as in Fig 3a, when using common average. **(B)** Similar and significant condition x half interaction when using common average.

Reviewer #2 (Remarks to the Author):

I thank the authors for carefully addressing my comments. I only have one comment regarding the code (see below). Other than that, the paper is suited for publication IMO.

We sincerely thank the reviewer for their constructive comments and careful reading, which have significantly improved the manuscript.

Reviewer #2 (Remarks on code availability):

In most script, path to data and other packages is hard coded, this makes it tedious for users to adapt the code. Specifically, a line like
settings.base_path_castle =
'/Users/kerrenadmin/Desktop/Other_projects/Dimensionality_ripples_Casper_and_Bernhard/';

should be:
settings.base_path_castle = [path_to_packages filesep path_to_CasperBernhard];

with path_to_packagee, path_to_CasperBernhard and other data and third party software properly defined in a proper configuration file.

We thank the reviewer for this suggestion. We have now removed hard-coded paths from the analysis scripts and instead implemented a dedicated configuration file (config_paths.m) in which all user-specific paths (e.g., data directories and third-party toolboxes) are defined using fullfile for cross-platform compatibility. This allows users to adapt the code by modifying a single configuration file without changing the analysis scripts themselves.

Reviewer #3 (Remarks to the Author):

The authors carefully addressed my comments. I have no further remarks, and I believe that the article is suitable for publication.

We are grateful for the reviewer's helpful feedback and engagement with the work, which has led to substantial improvements in the manuscript.

Reviewer #4 (Remarks to the Author):

The authors have addressed all points and provided additional information on their approaches; however, critical issues still persist. Unfortunately, these issues are likely to substantially affect the main results; therefore, they need to be resolved before the remainder of the results can be meaningfully evaluated.

We thank the reviewer for their thoughtful and critical comments, which have guided further improvements to the manuscript.

Major Concern A:
The employed ripple detection algorithm is problematic:
-1.5 standard deviations as threshold for ripple detection is unusually low, below common practice. With such a low threshold, the likelihood of false positive events will be increased (see Liu et al., 2022, consensus statement);
Given the current debate in the field, adapting a more conservative approach to ripple detection is highly recommended. The authors should repeat their analyses with a detection threshold of mean plus 3 standard deviations.

We thank the reviewer for this important comment and agree that the choice of detection threshold is a critical issue, particularly in light of ongoing discussions in the field (e.g., Liu et al. 2022 consensus statement on hippocampal ripples; van Schalkwijk & Helfrisch, 2026).

We would like to clarify that the ripple detection procedure used in our study differs from those evaluated in van Schalkwijk & Helfrisch (2026) and incorporates additional criteria designed to reduce false positives, as described in the manuscript.

To address the reviewer's concern, we repeated the key analyses using a more conservative detection threshold of mean + 3 standard deviations. As expected, this resulted in a reduced ripple density (AM+: M = .15Hz, SE = .02Hz; AM-: M = .12Hz, SE = .01Hz; before, AM+: M = .24Hz, SE = .03Hz, AM-: M= .19Hz, SE = .02Hz), still with a significant difference between conditions (t(1,11) = 2.95, p = .01), and did not alter any of the main results or conclusions. The key effects remained qualitatively and statistically unchanged (see below).

Specifically, we found an almost identical cluster as in Figure 3a when contrasting decoding AM+ and AM-, and a similar (and even statistically stronger) dimensionality difference pre vs post ripple as in Figure 3c for using a linear mixed-effects modelling (Estimate = -.13, SE = .02, t(19,988) = -6.16, p < .001).

Given the near-identical pattern of results across detection thresholds, we conclude that our findings are robust to the choice of threshold and are unlikely to be driven by false-positive ripple detections.

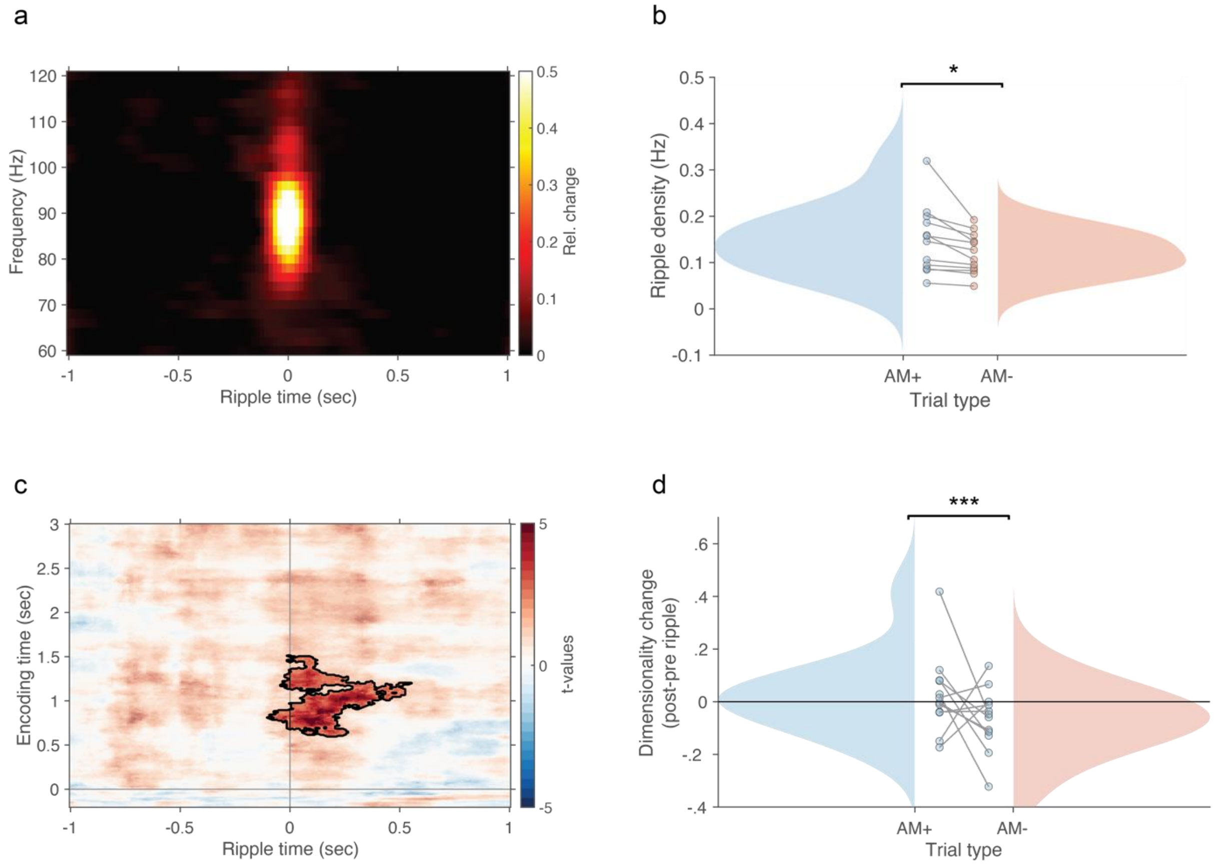

***Control analyses using standard deviation of 3*. a** Grand average hippocampal ripple for all participants, aligned to ripple peak at time 0, **b** Ripple density significantly differed between AM+ and AM-. Circles represent participants. **c** Stimulus-locked encoding x ripple-locked retrieval target decoding t-values. Results show significant increase of cortical target decodability for successful vs. unsuccessful retrieval following hippocampal ripple events (*p* < .05, corrected for multiple comparisons). **d** Linear mixed-effects modelling (Epoch Half: pre and post ripple, Trial Type: AM+ and AM-) of cortical dimensionality revealed a significant interaction, indicating a greater increase in dimensionality post-ripple for AM+ as compared to AM-. Asterisks denote significance for the interaction of pre and post ripple time-window and AM+ and AM-.

Ai) the authors might have misunderstood this point:
"present non-time-resolved power spectra based on the retrieval data. Specifically, they should compute a simple Fourier transform over 0-3 s and display the group average in Figure 2, as well as individual patient spectra in Figure S1."

The request was to show a power spectrum not centered on ripples, but instead over the whole retrieval period from 0 to 3 sec, during which ripples are assumed to occur. If there is a substantial amount of genuine ripple oscillations in the data, this should show as a peak in the power spectrum, independent of prior ripple detection.

Instead, the authors chose to center their data on detected ripple events, then compute a power spectrum; this approach was already used to create ripple centered TFRs. However, the outcome of this approach might be biased by centering data around transient sharp increases that might have passed the detection threshold. The resulting ripple centered TFR might look like a narrow band increase in power at ripple frequency, but cannot be distinguished from a smoothed, averaged broad band increase induced by transients.

We thank the reviewer for this comment and apologise for the earlier misunderstanding. We now appreciate that the request was to compute a power spectrum over the full retrieval period (0-3 secs), independent of ripple detection, rather than using ripple-centred data.

To address this, we computed a Fourier power spectrum across the entire retrieval window for each participant and present both the group-average spectrum and individual spectra (below), as requested. As anticipated given the transient and sparse nature of ripple events, the raw power spectra do not exhibit a prominent narrowband peak in the ripple frequency range. This is expected, as brief events with a low duty cycle are unlikely to dominate the time-averaged spectrum.

However, because neural power spectra follow a characteristic 1/f power-law distribution, narrowband oscillatory components can be masked by this broadband structure (Donoghue et al., 2020/2022). To account for this, we estimated the 1/f background by fitting a linear function in log-log space and divided the observed spectra by this fit. This 1/f-corrected representation revealed a clear peak in the ripple frequency range, consistent with the presence of genuine ripple oscillations in the data. In the revised manuscript, we have changed the inset in Figure 2a to show the mean and SE for this cue-locked analysis instead of the previous ripple-locked.

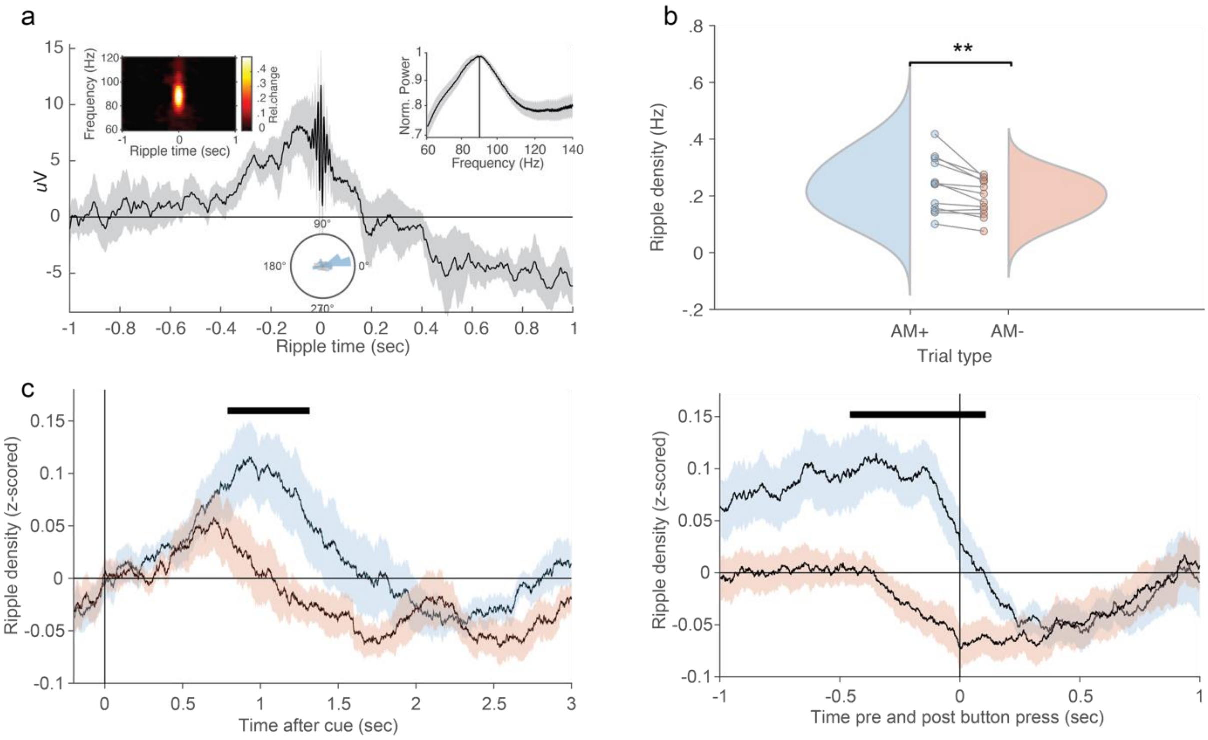

Updated Figure 2. Inset in Figure 2a is now showing 1/f-corrected FFT with mean and SE. Peak at 87Hz.

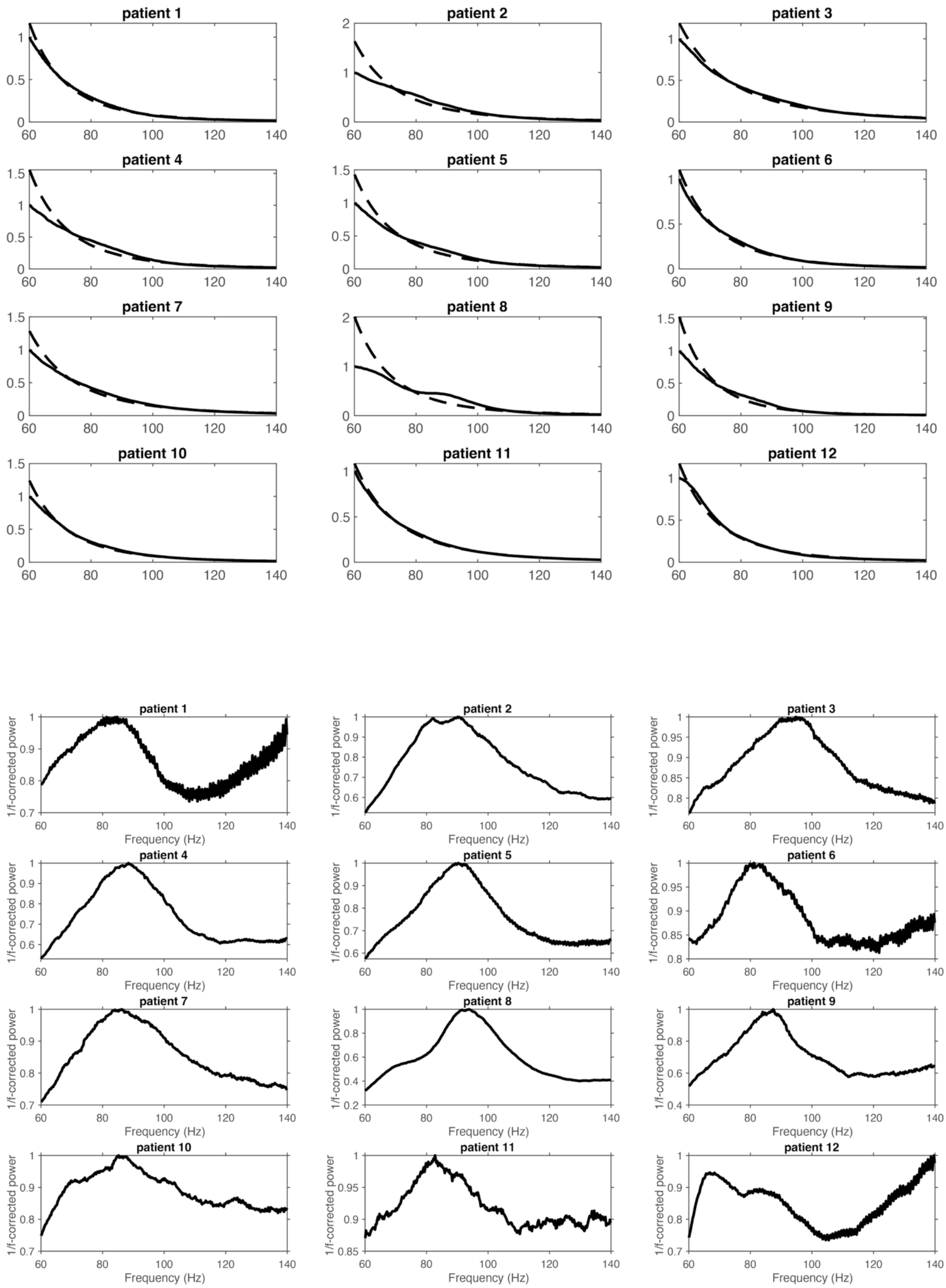

Fourier transformation of stimulus-locked data. Top. FFT of data (solid black lines) with 1/f power law fitted (dashed) for each patient. Bottom. Data divided by fitted 1/f power law shows clear peak in ripple range for most patients.

B)

-the proposed handling of potential epileptic activity is not ideal: instead of interpolating the spikes which can create problems in the data, a more conservative approach would be to exclude epochs around epileptic activity (+/- 1 second) and repeat the analyses.

We thank the reviewer for this important suggestion. To address this concern, we repeated the main analyses after excluding data within 1 second of detected spike events. Importantly, this exclusion was applied across all channels, such that ripple events were removed irrespective of whether they occurred on the same channel as the detected spike. The results remained qualitatively unchanged, indicating that our findings are not driven by epileptiform transients (see below).

We note that interictal spikes are brief, transient events, typically lasting on the order of tens to hundreds of milliseconds (e.g., Niedermeyer and da Silva 2011, *Electroencephalography: Basic Principles, Clinical Applications, and Related Fields*), with propagation occurring on similarly short timescales (e.g., Alarcon et al. 1994, *Intracerebral propagation of interictal activity in partial epilepsy: implications for source localisation*). As such, excluding 1 second already provides a conservative window that substantially exceeds the expected temporal extent of spike-related activity. Increasing the exclusion window further would result in a substantial loss of data without a clear physiological justification. Given that our results are robust to the exclusion within 1 secondn, we are confident that they are not driven by epileptiform activity. We have now included these control analyses as Supplementary Fig. 6 and thank the reviewer for this valuable suggestion.

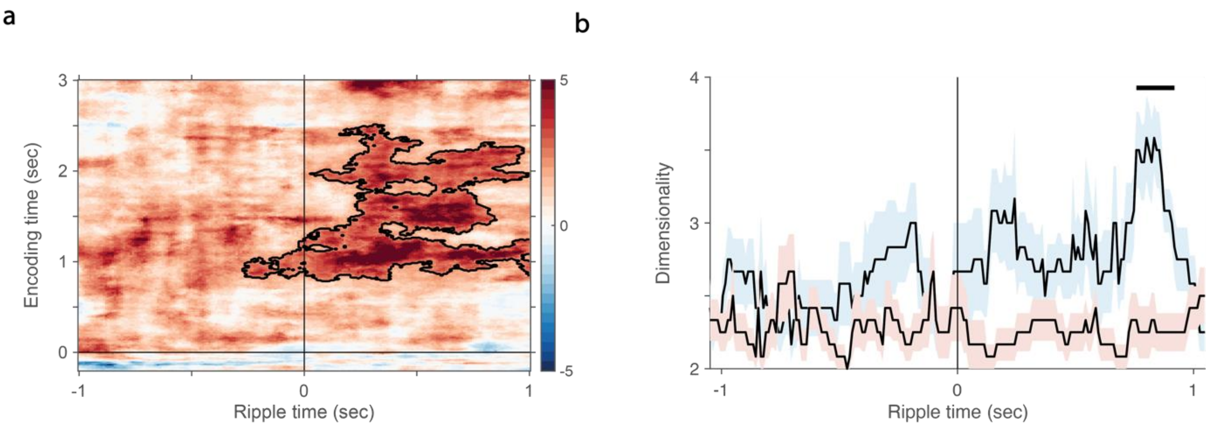

Spike-corrected reinstatement and dimensionality. ***a*** Reinstatement analysis excluding ripples occurring within 1 second of spikes in hippocampal channels revealed a similar significant cluster as in Fig. 3a. **b** The spike-corrected time-resolved dimensionality analysis showed a comparable significant cluster around 700–800 ms post-ripple, consistent with Fig. 3b.

-The depicted spike triggered averages do not resemble what is usually found when interictal epileptic activity is averaged: a spike-wave complex, which usually (but not always) shows in the erp. It seems the authors averaged across all detected spikes on all channels, without adjusting for polarity, which might explain the difference. Increasing the time around the spike might also help here.

We thank the reviewer for this comment and acknowledge that, in our original analysis, polarity was effectively handled at the channel level, which can obscure the characteristic spike-wave morphology when averaging across events. In the revised analysis, we instead

align polarity at the level of individual spikes prior to averaging. This results in a more representative spike-triggered waveform that better captures the expected morphology.

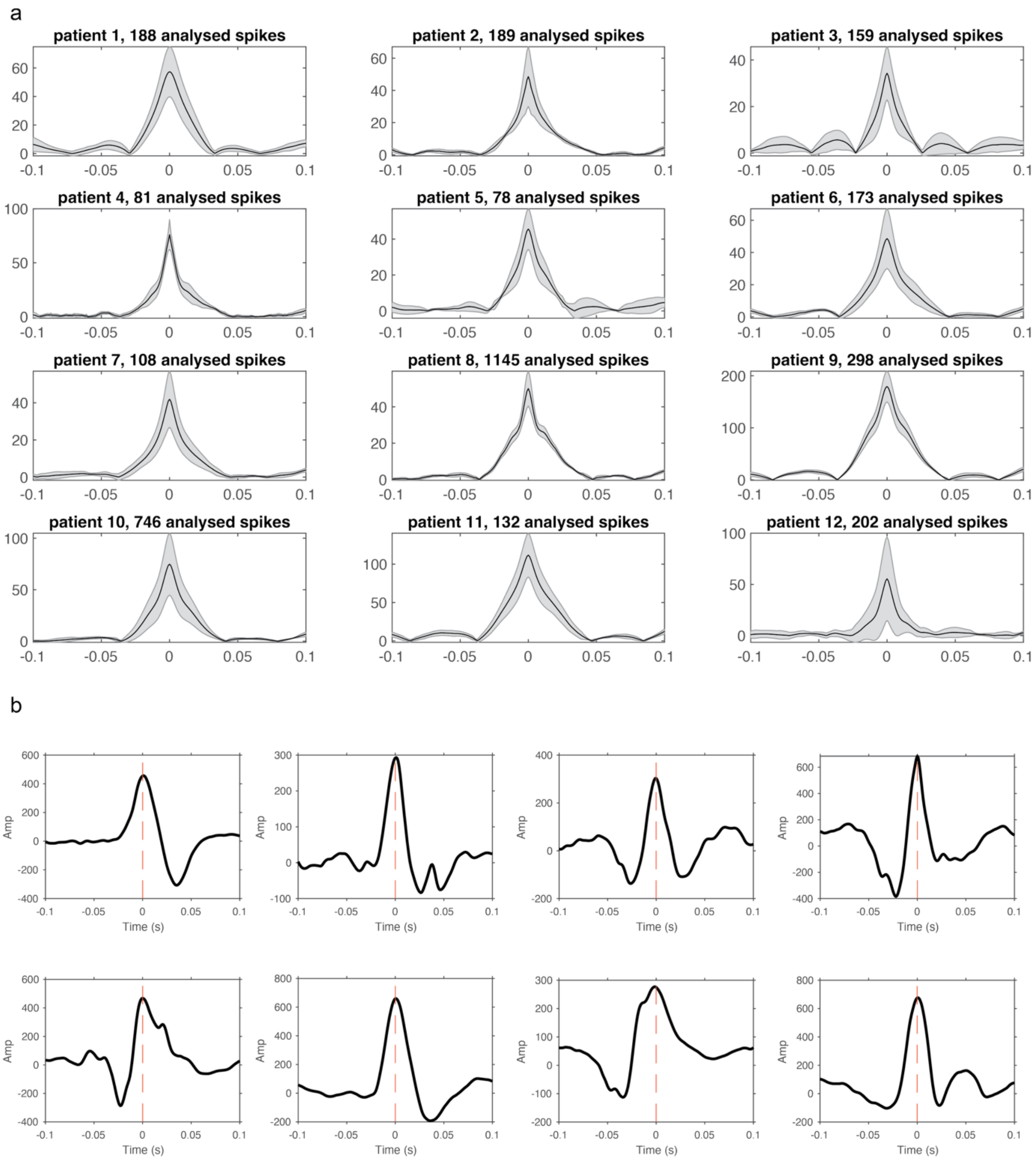

Supplementary Figure 14 | Spike-triggered averages. a. For each participant, we extracted and averaged spikes, with the number of spikes above each spike-triggered average. **b.** Example of extracted spikes.

A central concern for this manuscript is that IEDs themselves can drive hippocampus-to-cortex coupling, as demonstrated by Gelinas et al., 2016, Nature Medicine. This raises the possibility that the reported coupling effects may, at least in part, reflect IED-related dynamics rather than genuine ripple-driven interactions. The liberal ripple detection criteria (see above), together with uncertainties regarding IED detection and control, substantially weaken confidence in the present approach.

Gelinas et al 2016 and its implication for the findings presented here should also be thoroughly discussed in the manuscript.

We thank the reviewer for this comment and agree that this is an important concern to highlight and discuss in the manuscript. We have added the following to the Discussion:

A potential concern is that interictal epileptiform discharges (IEDs) themselves can drive hippocampus-cortex coupling, as demonstrated by Gelinas et al. (2016). This raises the possibility that ripple-related effects could, in principle, reflect IED-related dynamics rather than physiological ripple activity. However, several aspects of our data argue against this interpretation. First, ripple detection was restricted to hippocampal channels, whereas cortical dimensionality and reinstatement were assessed in anatomically distinct, non-hippocampal regions, reducing the likelihood that local epileptiform activity directly drives the observed cortical effects. Second, we explicitly controlled for interictal activity by excluding 1 second of data surrounding detected spikes across all channels, and the results remained unchanged. Third, interictal spikes are typically brief, spatially heterogeneous events that do not induce sustained, globally synchronous activity across distributed cortical regions. In contrast, the ripple-related effects we observe unfold over several hundred milliseconds and are associated with structured, condition-specific changes in cortical representational geometry. Together, these considerations suggest that the reported hippocampal-cortical interactions are unlikely to be explained by IED-related coupling and instead reflect physiological ripple-mediated communication.

Question on sufficient trial numbers:
multiple reviewers asked about don't know and incorrect responses in AM-. Since trial numbers in AM- can be as low as 19 (table S1), how many trials are left for truly incorrect reponses, which make only 32.5% of AM- trials? And is that number (6 trials in patient 11?) sufficient to have stable estimates in each of the patients? When contrasting, are trial numbers adjusted across conditions to account for more variance in low trial number conditions?

We thank the reviewer for this important comment. We agree that when AM- trials are further subdivided into incorrect and "don't know" responses, the number of trials per condition becomes relatively small in some participants. However, this subdivision was not part of our main analysis. For all primary analyses, we therefore collapsed these categories into a single AM- condition, ensuring sufficient trial numbers. At the group level, there was no systematic imbalance in trial counts between conditions (AM+: 58.67 ± 32.05, mean ± SD; AM-: 57 ± 31.87; $t(11) = 0.11$, $p = .91$). Importantly, the same set of channels was used across conditions, ensuring that differences cannot be attributed to variations in spatial sampling.

We have added a clarifying statement in the Methods section noting that when AM- trials are subdivided into incorrect and "don't know" responses for Supplementary Figure 4, the number of incorrect trials is limited for some participants, and these results should therefore be interpreted with caution.
